# Supplementary material for: Rapid diagnostic tests, laboratory-based immunoassay and nucleic acid testing strategies for long-acting injectable pre-exposure prophylaxis: A systematic review and meta-analysis
Source: PLoS Med. 2026 Apr 16;23(4):e1005030. doi: 10.1371/journal.pmed.1005030 (PMC13102303; doi:10.1371/journal.pmed.1005030)
Supplement: S1 Appendix — (DOCX) [file pmed.1005030.s001.docx]

# S1 Appendix. Search strategy protocol

**HIV testing in the context of long-acting pre-exposure prophylaxis (LAI-PrEP):**

**Systematic review**

**Background**

This systematic review will inform World Health Organization (WHO) guidelines on how best to implement HIV testing services within the context of long-acting pre-exposure prophylaxis (LA-PrEP). This may include testing prior to initiation and start of LA-PrEP, testing as monitoring while on LA-PrEP (which may include use that is either on-schedule or off-schedule dosing) and testing related to if/when an individual discontinues LA-PrEP. The following protocol provides a summary of the scope, definitions, and procedures that will be used in this review.

For the purposes of this review LA-PrEP is defined as a prevention option which does not require daily dosing and can be inserted, ingested, injected, infused, or implanted in a person’s body. This may range from monthly to annual dosing regimens which then provide sustained protection from acquiring HIV in those at substantial risk. Several delivery options are under investigation including intravaginal rings, injectable drugs, implants, and antibodies. Within these options, there are also several long-acting agents and formulations that may be in use, are under investigation (past or current) or are in the broader pipeline. These include long-acting injectable cabotegravir which is currently approved and recommended by WHO^1^, as well as lenacapavir and many others that may have been, or are still being investigated, in the context of research. Thus, we will aim to develop a common approach for reviewing LA-PrEP agents while also considering differences that may appear throughout the review.

In our primary review, we will include comparative studies which report on diagnostic performance and accuracy (e.g. sensitivity, specificity, positive-predictive value, negative-predictive value, concordance measures (i.e. kappa statistic), invalid/error rates of individual HIV tests and diagnostic algorithms) in the context of LA-PrEP delivery. While we will include and prioritize randomized trials and non-randomised comparative studies, we will not limit the review and will also include relevant observational and implementation studies.

Additional information on clinical utility will also be reviewed, such as uptake and effective use of LA-PrEP and potential benefits and harms (e.g. same day diagnosis, turnaround time, lost-to-follow-up, delayed injections, LEVI). Values related to outcomes and preferences of users and providers, and the costs and cost-effectiveness of different diagnostics and testing algorithms for LA-PrEP delivery will be summarized descriptively. Implementation considerations and findings from studies, which may vary by different product, programmatic implementation and testing time points and frequency of testing, will also be detailed and reported.

**Search methodology**

We will construct the searches as follows:

1) Long-acting

2) Preexposure prophylaxis

3) HIV

**1) AND 2) AND 3 OR** 4) Each long-acting PrEP on the market, such as cabotegravir or lenecapavir

The search strategy process is composed of identifying keywords, selecting databases, and developing the search strategies. The search strategy includes: 1) controlled vocabulary terms (where available), 2) relevant terms or synonyms, and 3) strings of terms. We will search OvidSP Medline, OvidSP Embase, Cochrane Central Register of Controlled Trials, CINALH databases. If the number of studies retrieved is insufficient, we will also search the Emcare database. We may search ClinicalTrials.gov for ongoing trials. No restrictions will be placed. Our search strategy is developed in the OvidSP Medline database with the assistance of an expert in systematic review (Lorena Romero). This search strategy is refined with Warittha Tieosapjaroen, Penny Presta (Monash librarian), Lucia Day (Alfred librarian) and Jason Ong until the results retrieved reflect the scope of the project.

The following databases will be searched

1. OvidSP MEDLINE® and Epub Ahead of Print, In-Process, In-Data-Review & Other Non-Indexed Citations, Daily and Version 1946 to Current
2. OvidSP Embase Classic+Embase 1947 to Current
3. Ovid Cochrane Central Register of Controlled Trials
4. EBSCOhost CINALH Complete
5. Clinicaltrials.gov

All citations identified will be imported into Endnote 21, where duplicates will be automatically removed.

Table A. Date of last search and number of publications identified from each database

| **Database** | **Publications identified** |
| --- | --- |
| Ovid Medline | 2006 |
| Ovid Embase | 4507 |
| CINAHL | 546 |
| Cochrane | 609 |

Date of last search: 18 October 2024

Table B. Number of publications imported into Covidence

| **Database** | **Publications imported** | **Duplicate** | **Total number of publications included** |
| --- | --- | --- | --- |
| Embase | 4507 | Duplicates 111 | 4396 |
| Medline | 2006 | Duplicates 1767 | 4635 |
| CINAHL | 546 | Duplicates 496 | 4685 |
| Cochrane | 609 | Duplicates 427 | 4867 |

**Search strategies**

Keywords

- 1. Injectable. long-acting, intravaginal ring, implant, antibody
  2. PrEP, pre-exposure prophylaxis, preexposure prophylaxis
  3. HIV
  4. Cabotegravir, CAB-LA, Lenacapavir, apretude, sunlenca, dapivirine ring
  5. (a AND b AND c) OR d

The gold set

1.Bekker 2024 - Twice-Yearly Lenacapavir or Daily FTAF

2. Koss 2024 - First Case of HIV Seroconversion With Integrase Resistance

3. Gonzalez 2023 - Development of a home-based pre-exposure prophylaxis care delivery system

4. Smith 2023- Predicted effects of the introduction of long-acting injectable cabotegravir

5. Fogel 2024 - Evaluation of Xpert point-of-care assays for detection of HIV infection

**Search strategies and results of each database**

1. OvidSP MEDLINE

**Database:**
Ovid MEDLINE(R) and Epub Ahead of Print, In-Process, In-Data-Review & Other Non-Indexed Citations, Daily and Versions <1946 to October 18, 2024>

**Link:**
[Click to run search](https://aus01.safelinks.protection.outlook.com/?url=https%3A%2F%2Furldefense.com%2Fv3%2F__https%3A%2F%2Faccess.ovid.com%2Fcustom%2Fredirector%2Findex.html%3Fdest%3Dhttps%3A**Ago.openathens.net*redirector*www.monash.edu*url%3Dhttp%3A**Aovidsp.ovid.com*ovidweb.cgi*T%3DJS%26NEWS%3DN%26PAGE%3Dmain%26SHAREDSEARCHID%3D5owPTHW13h403RPL1gBUsJI3sWxE3oFTdwusIEChttxB5hMAxA6A131sjukabNGEn__%3BLy8vLz8vLy8_!!NDYExDT0u85SdT4!oMX1xUhyf5_J1bkRwsH5DQ-rW-cDMqUruusPrgkk5mpJqw8EB9n-TRNCde9T_ioDSzMk1D1t9f9zkJp_FhMqV55AhQ%24&data=05%7C02%7C%7C2473e5e986fa477c1f7608dcef049d51%7Ce21c8db98aa843019657077a0415bdf1%7C0%7C0%7C638648050338311983%7CUnknown%7CTWFpbGZsb3d8eyJWIjoiMC4wLjAwMDAiLCJQIjoiV2luMzIiLCJBTiI6Ik1haWwiLCJXVCI6Mn0%3D%7C0%7C%7C%7C&sdata=UPjBDtBQcPrF8OJj9%2FKsB1fH2ZM6UfcpUIzgFG2Qj3Q%3D&reserved=0)
The above Jumpstart will only work for users who have access to this specific database.

**Database:**
Ovid MEDLINE(R) and Epub Ahead of Print, In-Process, In-Data-Review & Other Non-Indexed Citations, Daily and Versions <1946 to October 18, 2024>

Table C. Keywords and publications identified via OvidSP MEDLINE

| **#** | **Query** | **Results on 18 Oct 2024** |
| --- | --- | --- |
| 1 | "long acting".mp. [mp=title, book title, abstract, original title, name of substance word, subject heading word, floating sub-heading word, keyword heading word, organism supplementary concept word, protocol supplementary concept word, rare disease supplementary concept word, unique identifier, synonyms, population supplementary concept word, anatomy supplementary concept word] | 34,884 |
| 2 | inject*.mp. | 1,028,117 |
| 3 | Administration, Intravaginal/ | 5,423 |
| 4 | intravaginal.mp. | 9,891 |
| 5 | implant.mp. | 168,048 |
| 6 | exp immunoglobulins/ | 1,012,285 |
| 7 | Antibodies, Viral/ or Antibodies, Monoclonal/ or Antibodies, Monoclonal, Humanized/ or Antibodies, Neutralizing/ | 334,953 |
| 8 | antibod*.mp. | 1,329,839 |
| 9 | "sustain* release?".mp. [mp=title, book title, abstract, original title, name of substance word, subject heading word, floating sub-heading word, keyword heading word, organism supplementary concept word, protocol supplementary concept word, rare disease supplementary concept word, unique identifier, synonyms, population supplementary concept word, anatomy supplementary concept word] | 24,824 |
| 10 | "extend* release?".mp. [mp=title, book title, abstract, original title, name of substance word, subject heading word, floating sub-heading word, keyword heading word, organism supplementary concept word, protocol supplementary concept word, rare disease supplementary concept word, unique identifier, synonyms, population supplementary concept word, anatomy supplementary concept word] | 8,028 |
| 11 | "control?ed release?".mp. [mp=title, book title, abstract, original title, name of substance word, subject heading word, floating sub-heading word, keyword heading word, organism supplementary concept word, protocol supplementary concept word, rare disease supplementary concept word, unique identifier, synonyms, population supplementary concept word, anatomy supplementary concept word] | 23,950 |
| 12 | "slow release?".mp. [mp=title, book title, abstract, original title, name of substance word, subject heading word, floating sub-heading word, keyword heading word, organism supplementary concept word, protocol supplementary concept word, rare disease supplementary concept word, unique identifier, synonyms, population supplementary concept word, anatomy supplementary concept word] | 9,037 |
| 13 | "time release?".mp. [mp=title, book title, abstract, original title, name of substance word, subject heading word, floating sub-heading word, keyword heading word, organism supplementary concept word, protocol supplementary concept word, rare disease supplementary concept word, unique identifier, synonyms, population supplementary concept word, anatomy supplementary concept word] | 620 |
| 14 | "prolong* release?".mp. [mp=title, book title, abstract, original title, name of substance word, subject heading word, floating sub-heading word, keyword heading word, organism supplementary concept word, protocol supplementary concept word, rare disease supplementary concept word, unique identifier, synonyms, population supplementary concept word, anatomy supplementary concept word] | 2,874 |
| 15 | delayed-action preparations/ or drug implants/ | 50,731 |
| 16 | HIV Integrase Inhibitors/ | 2,437 |
| 17 | Pre-Exposure Prophylaxis/ | 5,617 |
| 18 | "prep".mp. [mp=title, book title, abstract, original title, name of substance word, subject heading word, floating sub-heading word, keyword heading word, organism supplementary concept word, protocol supplementary concept word, rare disease supplementary concept word, unique identifier, synonyms, population supplementary concept word, anatomy supplementary concept word] | 9,722 |
| 19 | "pre-exposure prophylaxis".mp. [mp=title, book title, abstract, original title, name of substance word, subject heading word, floating sub-heading word, keyword heading word, organism supplementary concept word, protocol supplementary concept word, rare disease supplementary concept word, unique identifier, synonyms, population supplementary concept word, anatomy supplementary concept word] | 8,215 |
| 20 | "preexposure prophylaxis".mp. | 1,491 |
| 21 | cabotegravir.mp. | 591 |
| 22 | "cab-la".mp. [mp=title, book title, abstract, original title, name of substance word, subject heading word, floating sub-heading word, keyword heading word, organism supplementary concept word, protocol supplementary concept word, rare disease supplementary concept word, unique identifier, synonyms, population supplementary concept word, anatomy supplementary concept word] | 68 |
| 23 | Lenacapavir.mp. [mp=title, book title, abstract, original title, name of substance word, subject heading word, floating sub-heading word, keyword heading word, organism supplementary concept word, protocol supplementary concept word, rare disease supplementary concept word, unique identifier, synonyms, population supplementary concept word, anatomy supplementary concept word] | 133 |
| 24 | (dapivirine and (vaginal or ring)).mp. [mp=title, book title, abstract, original title, name of substance word, subject heading word, floating sub-heading word, keyword heading word, organism supplementary concept word, protocol supplementary concept word, rare disease supplementary concept word, unique identifier, synonyms, population supplementary concept word, anatomy supplementary concept word] | 250 |
| 25 | apretude.mp. [mp=title, book title, abstract, original title, name of substance word, subject heading word, floating sub-heading word, keyword heading word, organism supplementary concept word, protocol supplementary concept word, rare disease supplementary concept word, unique identifier, synonyms, population supplementary concept word, anatomy supplementary concept word] | 8 |
| 26 | Sunlenca.mp. [mp=title, book title, abstract, original title, name of substance word, subject heading word, floating sub-heading word, keyword heading word, organism supplementary concept word, protocol supplementary concept word, rare disease supplementary concept word, unique identifier, synonyms, population supplementary concept word, anatomy supplementary concept word] | 9 |
| 27 | hiv.mp. [mp=title, book title, abstract, original title, name of substance word, subject heading word, floating sub-heading word, keyword heading word, organism supplementary concept word, protocol supplementary concept word, rare disease supplementary concept word, unique identifier, synonyms, population supplementary concept word, anatomy supplementary concept word] | 418,841 |
| 28 | HIV-1/ or HIV Infections/ or HIV/ or HIV Testing/ or HIV Antibodies/ or HIV-2/ | 285,174 |
| 29 | 27 or 28 | 418,841 |
| 30 | 1 or 2 or 3 or 4 or 5 or 6 or 7 or 8 or 9 or 10 or 11 or 12 or 13 or 14 or 15 or 16 | 2,789,439 |
| 31 | 17 or 18 or 19 or 20 | 12,563 |
| 32 | 29 and 30 and 31 | 1,316 |
| 33 | 21 or 22 or 23 or 24 or 25 or 26 or 32 | 2,006 |
|  | Check the search results against the gold set |  |
| 34 | (Twice-Yearly Lenacapavir or Daily FTAF for HIV Prevention in Cisgender Women).m_titl. | 4 |
| 35 | 33 and 34 | 4 |
| 36 | First Case of HIV Seroconversion With Integrase Resistance Mutations on Long-Acting Cabotegravir for Prevention in Routine Care.m_titl. | 1 |
| 37 | 33 and 36 | 1 |
| 38 | Development of a home-based pre-exposure prophylaxis care delivery system for long-acting injectable cabotegravir: a formative exploration of patient preferences.m_titl. | 1 |
| 39 | 33 and 38 | 1 |
| 40 | Predicted effects of the introduction of long-acting injectable cabotegravir pre-exposure prophylaxis in sub-Saharan Africa: a modelling study.m_titl. | 1 |
| 41 | 33 and 40 | 1 |
| 42 | Predicted effects of the introduction of long-acting injectable cabotegravir pre-exposure prophylaxis in sub-Saharan Africa: a modelling study.m_titl. | 1 |
| 43 | 33 and 42 | 1 |
| 44 | Evaluation of Xpert point-of-care assays for detection of HIV infection in persons using long-acting cabotegravir for pre-exposure prophylaxis.m_titl. | 1 |
| 45 | 33 and 44 | 1 |

1. OVID EMBASE

**Link:**
[Click to run search](https://aus01.safelinks.protection.outlook.com/?url=https%3A%2F%2Furldefense.com%2Fv3%2F__https%3A%2F%2Faccess.ovid.com%2Fcustom%2Fredirector%2Findex.html%3Fdest%3Dhttps%3A**Ago.openathens.net*redirector*www.monash.edu*url%3Dhttp%3A**Aovidsp.ovid.com*ovidweb.cgi*T%3DJS%26NEWS%3DN%26PAGE%3Dmain%26SHAREDSEARCHID%3D1JhACfA6o3yhICiunVfdV77NVqH0Hg3KUvDQXrJCkdtUwSQNgpwa2e9E7bJlYWwhM__%3BLy8vLz8vLy8_!!NDYExDT0u85SdT4!pZIfHh_uQNWzCcSwHKSYvntyfBJYk9b5JF6a_uuvz7uwL3A7jzfLt33RlaRdQDNpomc5OFxjICpaqVgfpcHEfVvChA%24&data=05%7C02%7C%7Ce058eb67511a490eebeb08dcef180fde%7Ce21c8db98aa843019657077a0415bdf1%7C0%7C0%7C638648133852422648%7CUnknown%7CTWFpbGZsb3d8eyJWIjoiMC4wLjAwMDAiLCJQIjoiV2luMzIiLCJBTiI6Ik1haWwiLCJXVCI6Mn0%3D%7C0%7C%7C%7C&sdata=sVoET0I24cDQqGgvLZWhw0PKNSowy799ieDgCqHeOro%3D&reserved=0)
The above Jumpstart will only work for users who have access to this specific database.


**Database:**
Embase Classic+Embase <1947 to 2024 October 16>

Table D. Keywords and publications identified via Ovid EMBASE

| **#** | **Query** | **Results fron 18 Oct 2024** |
| --- | --- | --- |
| 1 | "long acting".mp. [mp=title, abstract, heading word, drug trade name, original title, device manufacturer, drug manufacturer, device trade name, keyword heading word, floating subheading word, candidate term word] | 57,398 |
| 2 | inject*.mp. | 1,375,467 |
| 3 | intravaginal drug administration/ | 6,860 |
| 4 | intravaginal.mp. | 22,435 |
| 5 | vaginal ring/ | 2,723 |
| 6 | implant.mp. | 306,611 |
| 7 | antibody/ or exp autoantibody/ or exp drug antibody/ | 587,784 |
| 8 | antibod*.mp. | 1,872,715 |
| 9 | long acting drug/ | 8,266 |
| 10 | "sustain* release?".mp. | 46,218 |
| 11 | sustained drug release/ or controlled drug release/ | 37,998 |
| 12 | "extend* release?".mp. | 13,504 |
| 13 | "control?ed release?".mp. | 35,356 |
| 14 | controlled drug release/ or delayed drug release/ or pulsatile drug release/ or slow drug release/ or timed drug release/ | 19,113 |
| 15 | "slow release?".mp. | 12,401 |
| 16 | slow release formulation/ | 1,763 |
| 17 | "time release?".mp. | 874 |
| 18 | "prolong* release?".mp. | 4,486 |
| 19 | integrase inhibitor/ | 4,921 |
| 20 | pre-exposure prophylaxis/ | 11,321 |
| 21 | "prep".mp. | 20,138 |
| 22 | "pre-exposure prophylaxis".mp. | 12,951 |
| 23 | "preexposure prophylaxis".mp. | 2,010 |
| 24 | cabotegravir.mp. | 1,467 |
| 25 | cabotegravir/ or cabotegravir plus rilpivirine/ | 1,351 |
| 26 | "cab-la".mp. | 154 |
| 27 | Lenacapavir.mp. | 337 |
| 28 | lenacapavir/ | 314 |
| 29 | (dapivirine and (vaginal or ring)).mp. | 602 |
| 30 | dapivirine/ | 880 |
| 31 | apretude.mp. | 22 |
| 32 | Sunlenca.mp. | 28 |
| 33 | hiv.mp. | 489,820 |
| 34 | exp Human immunodeficiency virus/ | 227,446 |
| 35 | exp HIV test/ | 17,213 |
| 36 | exp Human immunodeficiency virus infection/ | 837,876 |
| 37 | exp acquired immune deficiency syndrome/ | 585,205 |
| 38 | aids.mp. | 227,074 |
| 39 | acquire? immun* deficiency syndrome.mp. | 146,601 |
| 40 | 20 or 21 or 22 or 23 | 24,903 |
| 41 | 33 or 34 or 35 or 36 or 37 or 38 or 39 | 1,097,722 |
| 42 | 1 or 2 or 3 or 4 or 5 or 6 or 7 or 8 or 9 or 10 or 11 or 12 or 13 or 14 or 15 or 16 or 17 or 18 or 19 | 3,626,120 |
| 43 | 40 and 41 and 42 | 2,539 |
| 44 | 24 or 25 or 26 or 27 or 28 or 29 or 30 or 31 or 32 or 43 | 4,507 |

1. OVID Cochrane Central Registry of Controlled Trials

**Link:**
[Click to run search](https://aus01.safelinks.protection.outlook.com/?url=https%3A%2F%2Furldefense.com%2Fv3%2F__https%3A%2F%2Faccess.ovid.com%2Fcustom%2Fredirector%2Findex.html%3Fdest%3Dhttps%3A**Ago.openathens.net*redirector*www.monash.edu*url%3Dhttp%3A**Aovidsp.ovid.com*ovidweb.cgi*T%3DJS%26NEWS%3DN%26PAGE%3Dmain%26SHAREDSEARCHID%3D1Jn6BFSClXCKMAvC1NDRls0uvXQdZnB6DYrzNXCbRvDej3aEcEhKIrswHZboo4lg8__%3BLy8vLz8vLy8_!!NDYExDT0u85SdT4!uFNrYZL3kUbu2jmsd5TUgzxKRKEFARq4rhj4Y6_-7tzAi6uxRYUKsUDx_mFlTe_GT419zH4NA6PqTo2dyz6Qq1LomA%24&data=05%7C02%7C%7Cd99c24860c8c4e61be7e08dcef1c2217%7Ce21c8db98aa843019657077a0415bdf1%7C0%7C0%7C638648151345646624%7CUnknown%7CTWFpbGZsb3d8eyJWIjoiMC4wLjAwMDAiLCJQIjoiV2luMzIiLCJBTiI6Ik1haWwiLCJXVCI6Mn0%3D%7C0%7C%7C%7C&sdata=FR172tJvUVA%2FvQMq93FPhXZgqc1Ic1W5X6HJzQ6YKmc%3D&reserved=0)
The above Jumpstart will only work for users who have access to this specific database.


**Database:**
EBM Reviews - Cochrane Central Register of Controlled Trials <September 2024>

Table E. Keywords and publications identified via Ovid Cochrane Central Registry of Controlled Trials

| **#** | **Query** | **Results from 18 Oct 2024** |
| --- | --- | --- |
| 1 | "long acting".mp. [mp=title, original title, abstract, floating sub-heading word, mesh headings, heading words, keyword] | 11,311 |
| 2 | inject*.mp. | 134,313 |
| 3 | Administration, Intravaginal/ | 1,829 |
| 4 | intravaginal.mp. | 3,705 |
| 5 | implant.mp. | 17,299 |
| 6 | exp immunoglobulins/ | 39,627 |
| 7 | Antibodies, Viral/ or Antibodies, Monoclonal/ or Antibodies, Monoclonal, Humanized/ or Antibodies, Neutralizing/ | 16,942 |
| 8 | antibod*.mp. | 58,670 |
| 9 | "sustain* release?".mp. [mp=title, original title, abstract, floating sub-heading word, mesh headings, heading words, keyword] | 4,807 |
| 10 | "extend* release?".mp. [mp=title, original title, abstract, floating sub-heading word, mesh headings, heading words, keyword] | 5,844 |
| 11 | "control?ed release?".mp. [mp=title, original title, abstract, floating sub-heading word, mesh headings, heading words, keyword] | 2,204 |
| 12 | "slow release?".mp. [mp=title, original title, abstract, floating sub-heading word, mesh headings, heading words, keyword] | 2,128 |
| 13 | "time release?".mp. [mp=title, original title, abstract, floating sub-heading word, mesh headings, heading words, keyword] | 59 |
| 14 | "prolong* release?".mp. [mp=title, original title, abstract, floating sub-heading word, mesh headings, heading words, keyword] | 1,198 |
| 15 | delayed-action preparations/ or drug implants/ | 7,690 |
| 16 | HIV Integrase Inhibitors/ | 158 |
| 17 | Pre-Exposure Prophylaxis/ | 488 |
| 18 | "prep".mp. [mp=title, original title, abstract, floating sub-heading word, mesh headings, heading words, keyword] | 2,253 |
| 19 | "pre-exposure prophylaxis".mp. [mp=title, original title, abstract, floating sub-heading word, mesh headings, heading words, keyword] | 1,396 |
| 20 | "preexposure prophylaxis".mp. | 184 |
| 21 | cabotegravir.mp. | 182 |
| 22 | "cab-la".mp. [mp=title, original title, abstract, floating sub-heading word, mesh headings, heading words, keyword] | 69 |
| 23 | Lenacapavir.mp. [mp=title, original title, abstract, floating sub-heading word, mesh headings, heading words, keyword] | 48 |
| 24 | (dapivirine and (vaginal or ring)).mp. [mp=title, original title, abstract, floating sub-heading word, mesh headings, heading words, keyword] | 167 |
| 25 | apretude.mp. [mp=title, original title, abstract, floating sub-heading word, mesh headings, heading words, keyword] | 2 |
| 26 | Sunlenca.mp. [mp=title, original title, abstract, floating sub-heading word, mesh headings, heading words, keyword] | 1 |
| 27 | hiv.mp. [mp=title, original title, abstract, floating sub-heading word, mesh headings, heading words, keyword] | 32,558 |
| 28 | HIV-1/ or HIV Infections/ or HIV/ or HIV Testing/ or HIV Antibodies/ or HIV-2/ | 16,216 |
| 29 | 27 or 28 | 32,558 |
| 30 | 1 or 2 or 3 or 4 or 5 or 6 or 7 or 8 or 9 or 10 or 11 or 12 or 13 or 14 or 15 or 16 | 235,470 |
| 31 | 17 or 18 or 19 or 20 | 2,624 |
| 32 | 29 and 30 and 31 | 281 |
| 33 | 21 or 22 or 23 or 24 or 25 or 26 or 32 | 609 |

1. CINAHL

(

("long acting" or injectable or injection# or intravaginal or implant or antibod* or "sustained release#" or "extend* release#" or "control#ed release#" or "slow release#" or "time release#" or "prolong* release#" or “integrase inhibitor”)

AND

("prep" or "pre-exposure prophylaxis" or "preexposure prophylaxis")

AND “HIV”

)

OR

cabotegravir OR “CAB LA” OR “lenacapavir” OR “dapivirine ring” OR “dapivirine vaginal ring#”OR “apretude” OR “Sunlenca”


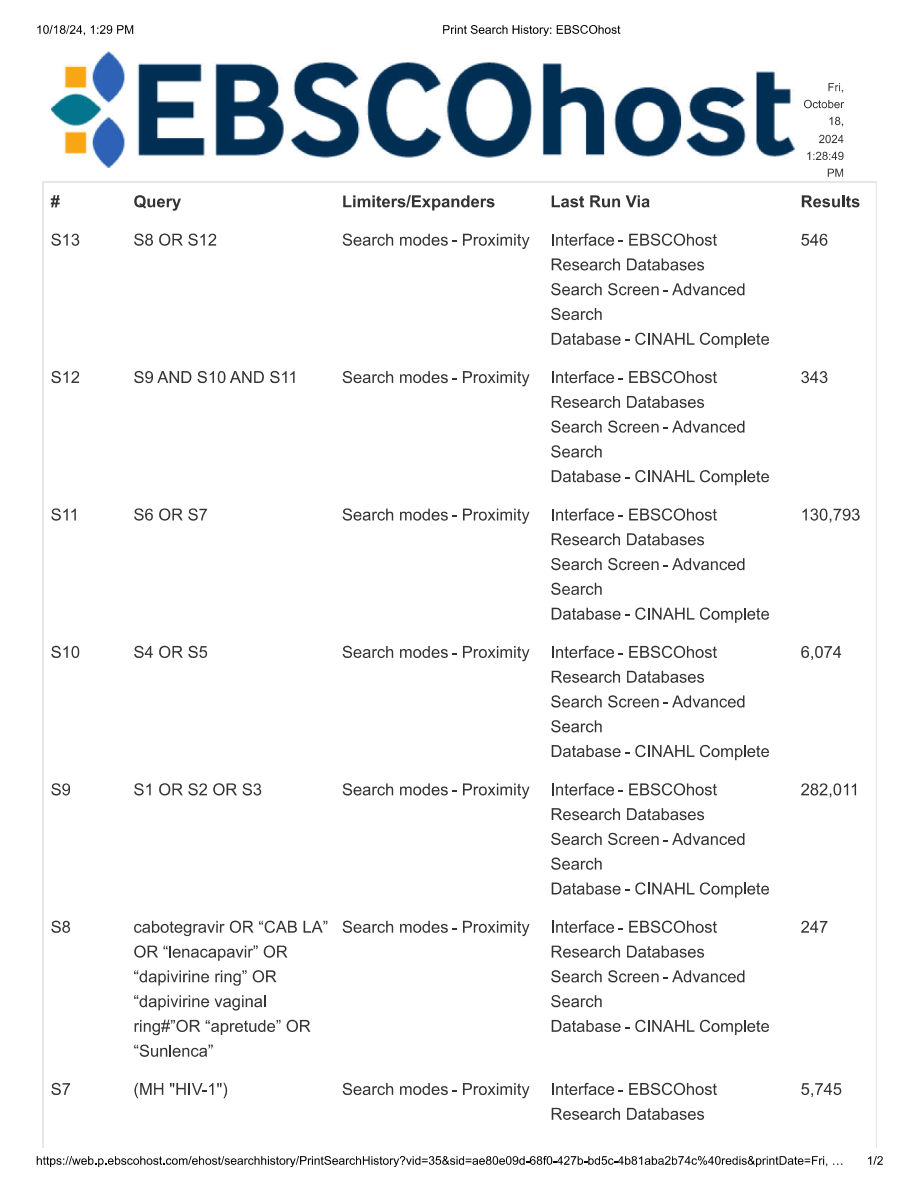


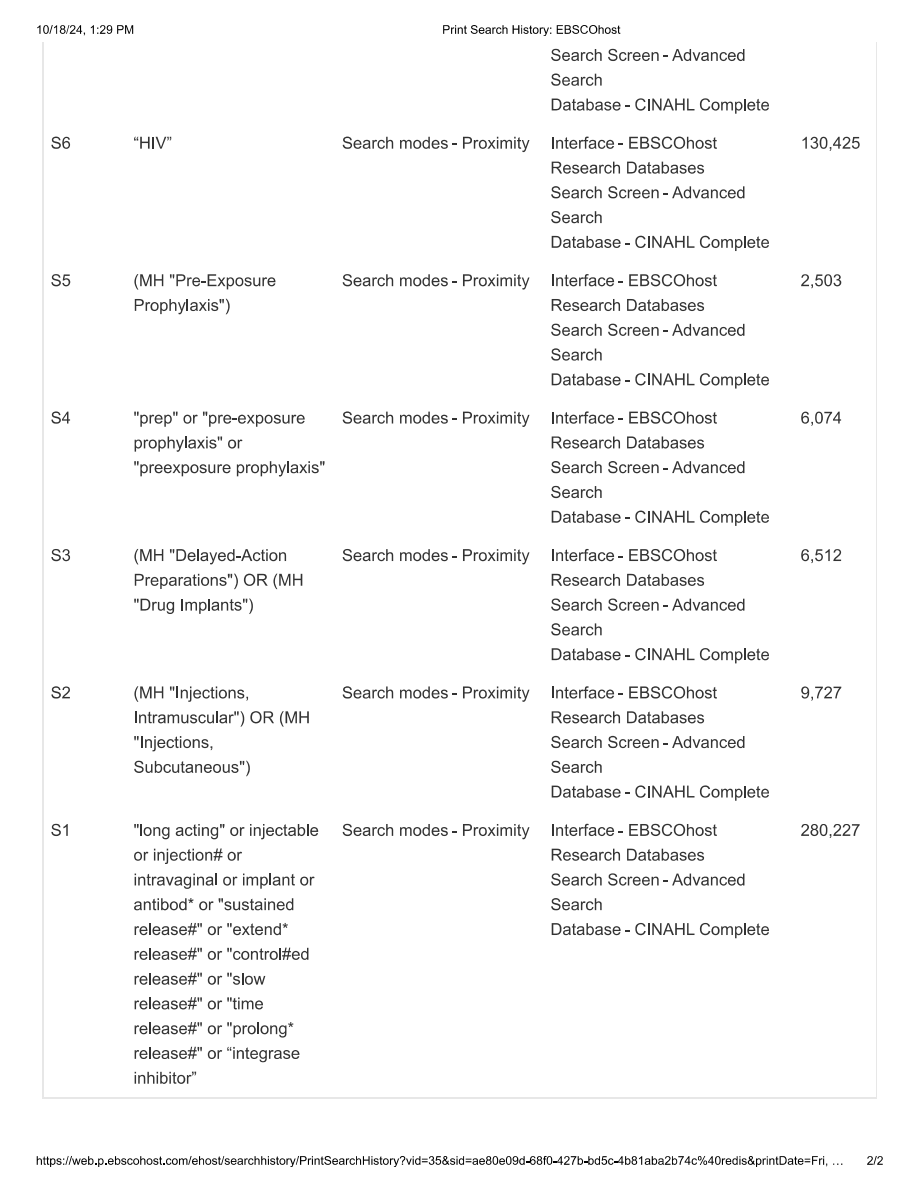


Figure A. Keywords and publications identified via CINAHL

1. Clinicaltrial.gov

**Disease:** HIV

**Other terms:** HIV infections

**Interventions:**

(("long acting" or injectable or injection? or intravaginal or implant or antibod* or "sustained release?" or "extend* release?" or "control?ed release?" or "slow release?" or "time release?" or "prolong* release?" or “integrase inhibitor”) AND ("prep" or "pre-exposure prophylaxis" or "preexposure prophylaxis")) OR cabotegravir OR “CAB LA” OR “lenacapavir” OR “dapivirine ring” OR “dapivirine vaginal ring?”OR “apretude” OR “Sunlenca”
