# Supplementary material for: Rapid diagnostic tests, laboratory-based immunoassay and nucleic acid testing strategies for long-acting injectable pre-exposure prophylaxis: A systematic review and meta-analysis
Source: PLoS Med. 2026 Apr 16;23(4):e1005030. doi: 10.1371/journal.pmed.1005030 (PMC13102303; doi:10.1371/journal.pmed.1005030)
Supplement: S2 Appendix — (DOCX) [file pmed.1005030.s002.docx]

# S2 Appendix. Definition of outcomes

## Clinical outcomes

1. Time to linkage and ART initiation from date of acquisition of sample found to first be confirmed of HIV infection (e.g. median number of days between diagnosis and ART start and/or virologic outcome at 6 mos/12mos after initiation of ART (by type of ART/PrEP regimen)
2. PrEP holds or discontinuations triggered from testing found to be false positive (e.g. number and proportion of people with delayed or discontinued PrEP use out of all those tested in RDT/ST algorithms vs RNA-based algorithms)
3. Testing frequency (e.g. measured as the number of time points an individual is tested for HIV, may include additional testing services for STIs as well)
4. Sexual risk behaviours of those with misdiagnosis (e.g., measured as report of condomless sex, sexual transmitted infections, or number of sexual partners).
5. HIV positivity (e.g., HIV-positive diagnosis among all participants initiating or restarting LA-PrEP)
6. Clinical/social harms: number and proportion of testers who experienced social harm/adverse events (e.g., misdiagnosis)

## Diagnostic outcomes

1. Diagnostic accuracy and performance of rapid tests and diagnostic testing strategies and algorithms that only using HIV rapid tests and/or self-tests among participants starting, continuing or discontinuing LA-PrEP (e.g.sensitivity, specificity, positive-predictive value, negative-predictive value, number false positive, number false negative, concordance measures (i.e. kappa statistic), error rate/invalid rates) compared to NAT testing and NAT-based testing algorithms
2. Time of detection: Early HIV or acute HIV infection (AHI) detection (e.g. number of early or acute infections detected and/or missed in RDT/ST algorithms vs RNA-based algorithms) If data are available, timing of test reactivity compared to first available evidence of infection.
3. Resistance-associated mutations detected at first evidence of HIV infection (e.g. number and proportion of mutations reported out of all those tested in RDT/ST algorithms vs RNA-based algorithms)
4. Median turnaround time of test results among participants starting, continuing or discontinuing LA-PrEP with RDT/ST strategies and algorithms vs RNA-based testing strategies and algorithms (e.g. time from initial testing to return of results and LA-PrEP use)
