# Supplementary material for: Rapid diagnostic tests, laboratory-based immunoassay and nucleic acid testing strategies for long-acting injectable pre-exposure prophylaxis: A systematic review and meta-analysis
Source: PLoS Med. 2026 Apr 16;23(4):e1005030. doi: 10.1371/journal.pmed.1005030 (PMC13102303; doi:10.1371/journal.pmed.1005030)
Supplement: S3 Appendix — (DOCX) [file pmed.1005030.s003.docx]

# S3 Appendix. Risk of bias assessments

Among non-randomised comparator studies, three had a low risk of bias, and five had a critical risk of bias. Among diagnostic accuracy studies, one had a low risk of bias, five had a high risk of bias, and two were unclear. The critical and high risk of bias was due to study designs that relied on RDT or laboratory-based immunoassay results prior to NAT testing, introducing the possibility that false-negative RDT or laboratory-based immunoassay results may have led to missed HIV infections, as these participants did not proceed to NAT testing.

- Table A. Risk of bias assessment using ROBINS-I V2

|  | CATALYST | FASTPrEP | HPTN083 | HPTN084 | PURPOSE 1 | PURPOSE 2 | SEARCH SAPPHIRE |
| --- | --- | --- | --- | --- | --- | --- | --- |
| 1. Bias due to confounding | NA | NA | NA | NA | NA | NA | NA |
| 2. Bias in classification of interventions | Low | Low | Critical | Critical | Critical | Critical | Low |
| 3. Bias in selection of participants into the study (or into the analysis) | Low | Low | Low | Low | Low | Low | Low |
| 4. Bias due to deviations from intended interventions | Low | Low | Low | Low | Low | Low | Low |
| 5. Bias due to missing data | NA | NA | Low | Low | Low | Low | Low |
| 6. Bias in measurement of the outcome | Low | Low | Serious | Serious | Serious | Serious | Low |
| 7. Bias in selection of the reported result | Low | Low | Low | Low | Low | Low | Low |
| **Overall risk of bias** (Low/Moderate/Serious/Critical) | Low | Low | Critical | Critical | Critical | Critical | Low |

- Table B. Risk of bias assessment using QUADAS 2

|  | CATALYST | FASTPrEP | HPTN083 | HPTN084 | PURPOSE 1 | PURPOSE 2 | SEARCH SAPPHIRE |
| --- | --- | --- | --- | --- | --- | --- | --- |
| **Domain 1:** PATIENT SELECTION |  |  |  |  |  |  |  |
| A. Risk of Bias | Low | Low | Low | Low | Low | Low | Low |
| B. Concerns regarding applicability | Low | Low | Low | Low | Low | Low | Low |
| **Domain 2:** INDEX TEST(S) |  |  |  |  |  |  |  |
| A. Risk of Bias | Low | Low | Low | Low | Low | Low | Low |
| B. Concerns regarding applicability | Low | Low | Low | Low | Low | Low | Low |
| **Domain 3:** REFERENCE STANDARD |  |  |  |  |  |  |  |
| A. Risk of Bias | Low | Low | High | High | High | High | Low |
| B. Concerns regarding applicability | Low | Low | Low | Low | Low | Low | Low |
| **Domain 4:** FLOW AND TIMING |  |  |  |  |  |  |  |
| A. Risk of Bias | Unclear | Unclear | High | High | High | High | Low |
