# Supplementary material for: Rapid diagnostic tests, laboratory-based immunoassay and nucleic acid testing strategies for long-acting injectable pre-exposure prophylaxis: A systematic review and meta-analysis
Source: PLoS Med. 2026 Apr 16;23(4):e1005030. doi: 10.1371/journal.pmed.1005030 (PMC13102303; doi:10.1371/journal.pmed.1005030)
Supplement: S4 Appendix — (DOCX) [file pmed.1005030.s004.docx]

# S4 Appendix. Certainty of evidence (GRADE)

**Question:** Should HIV testing services with rapid diagnostic tests and/or self-tests be used as part of long-acting PrEP (LA-PrEP) services (including initiation, continuation/monitoring and/or discontinuation)?

**Setting:** Global

**Notes:**

- Data arising from RCTs (e.g. HPTN 083, HPTN 084, PURPOSE-1, PURPOSE-2) have been classified as non-randomised comparator studies in this GRADE table because their original study design was not for evaluating the type of testing algorithm (thus the point of randomisation was for the type of PrEP, not the HIV test algorithm). We compared outcomes from RDT vs. NAT-based algorithms when data was available for NATs retrospectively conducted on blood samples from each study time point. We constructed a hypothetical scenario from which a NAT-based algorithm was used instead (e.g. if RDT detected HIV at month 6, but a retrospective analysis of blood samples using NAT detected HIV using the month 4 blood samples, we would infer a delay of 2 months for detection of HIV when using RDT-based vs. NAT-based algorithm.
- Outcomes data on HIVST was only available for testing frequency. Indirect evidence demonstrates that HIVST may be comparable to RDTs in terms of time to linkage and ART initiation(Zhang et al., 2024) and diagnostic accuracy.(Figueroa et al., 2018)

Table A. Certainty of evidence assessment (GRADE)

| **Certainty assessment** | | | | | | | **№ of patients** | | **Effect** | | **Certainty** | **Importance** |
| --- | --- | --- | --- | --- | --- | --- | --- | --- | --- | --- | --- | --- |
| **№ of studies** | **Study design** | **Risk of bias** | **Inconsistency** | **Indirectness** | **Imprecision** | **Other considerations** | **Rapid Diagnostic Tests** | **NAT** | **Relative (95% CI)** | **Absolute (95% CI)** |  |  |
| 1) Time to linkage and ART initiation from date of acquisition of sample found to first be confirmed of HIV infection (e.g. median number of days between diagnosis and ART start and/or virologic outcome at 6 mos/12mos after initiation of ART (by type of ART/PrEP regimen) | | | | | | | | | | | | |
| 4^1,2,3,4^ | Observational cohorts / case report/series | serious^a^ | not serious | serious^b^ | serious^c^ | none | Two studies from the US (SeroPrEP and Case Report) used NAT (and lab-based Ag/Ab test) as their testing algorithm and reported that two individuals diagnosed with HIV took six and nine days to initiate ART post-diagnosis, respectively. One study (Zimbabwe observational cohort)4 used 3rd Gen RDT as their testing algorithm one individual diagnosed with HIV took one day to initiate ART post-diagnosis. One study (CATALYST) used 3rd/4th Gen RDT + NAT reported time to linkage but no ART initiation data yet: one case returned 8 days post injection and one case returned 21 days post injection. | | | | ⨁◯◯◯ Very low | CRITICAL |

| 2a) PrEP holds or discontinuations triggered from testing found to be false positive (CAB) (e.g. number and proportion of people with delayed or discontinued PrEP use out of all those tested in RDT/ST algorithms vs RNA-based algorithms) | | | | | | | | | |
| --- | --- | --- | --- | --- | --- | --- | --- | --- | --- |
| 1^5^ | non-randomised studies | serious^d^ | Cannot be judged | serious^b^ | serious^c^ | none | This was based on data from the open-label extension of the HPTN 083 trial, where 22/2483 (0.9%) individuals received a false positive RNA test, resulting in four who delayed CAB-LA initiation, two who discontinued CAB-LA and one who had delayed oral CAB; none of these seven individuals had evidence of HIV. In total, 20/2483 (0.8%) of individuals received a false positive RDT (unspecified) and/or lab-based Ag/Ab test, but no further data was available if this resulted in PrEP holds or discontinuation. Similar tests are used to confirm or resolve a reactive test, so it is presumed that the impact on PrEP holds/discontinuation from false positives from either HIV testing algorithm will be similar. | ⨁◯◯◯ Very low | CRITICAL |
| 2b) PrEP holds or discontinuations triggered from testing found to be false positive (CAB) (e.g. number and proportion of people with delayed or discontinued PrEP use out of all those tested in RDT/ST algorithms vs RNA-based algorithms) | | | | | | | | | |
| 1^4^ | Observational cohort | serious^d^ | Cannot be judged | serious^b^ | serious^c^ | none | CATALYST study reported 2/1010 (0.2%) of individuals with a false positive RNA test, however, both decided to continue with CAB LA | ⨁◯◯◯ Very low | CRITICAL |

| 3a) HIV Positivity (Continuation, CAB + LEN) (e.g., HIV-positive diagnosis among all participants initiating or restarting LA-PrEP) | | | | | | | | | | | | |
| --- | --- | --- | --- | --- | --- | --- | --- | --- | --- | --- | --- | --- |
| 4^6^ | non-randomised studies | serious^e^ | not serious | serious^b^ | not serious^f^ | none | 9/8171 (0.1%) | 14/8171 (0.2%) | **OR 0.66*** (0.29 to 1.50) | **1 fewer per 1,000** (from 1 fewer to 1 more) | ⨁⨁◯◯ Low | CRITICAL |
| * There was no statistically significant difference in HIV positivity using RDT vs. NAT (p-value 0.40), nor RDT vs. Lab-based Ag/Ab (p-value 0.52), using Chi-square with Yates correction.  3b) HIV Positivity (Continuation, CAB) (e.g., HIV-positive diagnosis among all participants initiating or restarting LA-PrEP) | | | | | | | | | | | | |
| 2^7^ | non-randomised studies | serious^g^ | not serious | serious^b^ | not serious^f^ | none | 8/3858 (0.2%) | 12/3858 (0.3%) | **OR 0.68*** (0.28 to 1.65) | **1 fewer per 1,000** (from 2 fewer to 2 more) | ⨁⨁◯◯ Low | CRITICAL |
| * There was no statistically significant difference in HIV positivity using RDT vs. NAT (p-value 0.66), nor RDT vs. Lab-based Ag/Ab (p-value 0.82), using Chi-square with Yates correction.  3c) HIV Positivity (Continuation, LEN) (e.g., HIV-positive diagnosis among all participants initiating or restarting LA-PrEP) | | | | | | | | | | | | |
| 2^8^ | non-randomised studies | serious^h^ | not serious | serious^b^ | not serious^f^ | none | 1/4313 (0.0%) | 2/4313 (0.0%) | **OR 0.50*** (0.05 to 5.52) | **0 fewer per 1,000** (from 0 fewer to 2 more) | ⨁⨁◯◯ Low | CRITICAL |
| * There was no statistically significant difference in HIV positivity using RDT vs. NAT (p-value 0.56), nor RDT vs. Lab-based Ag/Ab (p-value 0.56), using Chi-square with Yates correction. 4a) Diagnostic accuracy and performance of rapid tests and diagnostic testing strategies and algorithms that only using HIV rapid tests and/or self-tests among participants starting, continuing or discontinuing LA-PrEP (at initiation, CAB + LEN) | | | | | | | | | | | | |
| 4^6^ | non-randomised studies | serious^i^ | not serious | serious^b^ | not serious^f^ | none | Using an RDT-based algorithm (compared to NAT) has a negative predictive value (NPV) of 99.8% (95% CI: 99.8-99.9%). The number of true negatives was 8204, and the false negatives was 13. Using a lab-based Ag/Ab strategy (compared to NAT) has an NPV of 99.9% (99.9-100%). The number of true negatives was 8204, and the false negatives was 7.  ^j^ | | | | ⨁⨁◯◯ Low | CRITICAL |
| 4b) Diagnostic accuracy and performance of rapid tests and diagnostic testing strategies and algorithms that only using HIV rapid tests and/or self-tests among participants starting, continuing or discontinuing LA-PrEP (at initiation, CAB) | | | | | | | | | | | | |
| 2^7^ | non-randomised studies | serious^g^ | not serious | serious^b^ | not serious^f^ | none | Using an RDT-based algorithm (compared to NAT) has a negative predictive value (NPV) of 99.9% (95% CI: 99.7-100%). The number of true negatives was 3891, and the false negatives was 5. Using a lab-based Ag/Ab test algorithm (compared to NAT) has an NPV of 99.9% (99.7-100%). The number of true negatives was 3891 and false negatives was 4.  ^j^ | | | | ⨁⨁◯◯ Low | CRITICAL |
| 4c) Diagnostic accuracy and performance of rapid tests and diagnostic testing strategies and algorithms that only using HIV rapid tests and/or self-tests among participants starting, continuing or discontinuing LA-PrEP (at initiation, LEN) | | | | | | | | | | | | |
| 2^8^ | non-randomised studies | serious^h^ | not serious | serious^b^ | not serious^f^ | none | Using an RDT-based algorithm (4th Gen RDT) (compared to NAT) has a negative predictive value (NPV) of 99.8% (95% CI: 99.6-99.5%). The number of true negatives was 4313, and the false negatives was 8. Using a lab-based Ag/Ab test strategy (compared to NAT) has an NPV of 99.9% (99.8-100%). The number of true negatives was 4313, and the false negatives was 3.  ^j^ | | | | ⨁⨁◯◯ Low | CRITICAL |
| 4d) Diagnostic accuracy and performance of rapid tests and diagnostic testing strategies and algorithms that only using HIV rapid tests and/or self-tests among participants starting, continuing or discontinuing LA-PrEP (with continuation, CAB) | | | | | | | | | | | | |
| 1^9^ | non-randomised studies | serious^k^ | Cannot be judged | serious^b^ | not serious^f^ | none | Using data from HPTN 083 Open-label extension, RDT & Lab-based Ag/Ab has a sensitivity of 82.8% [95% CI: 65.5-92.4] and positive predictive value (PPV) of 56.9% (43.3-69.5%). The number of true positives was 24, false positives was 20, true negatives was 26477 and false negatives was 5. RNA NAT had a sensitivity of 100% [88.3-100%] and PPV of 54.6% [40.1-68.3%]. The number of true positives was 29, false positives was 22, true negatives was 26475 and false negatives was 0. If the CAB injection was within 6 months, NAT sensitivity was 87.5% [46.7-99.3] and PPV was 9.1% [1.6-30.6]. However, if the CAB injection was after 6 months, NAT sensitivity was 100% [80-100] and PPV was 60% [17-92.7%].  ^j^ | | | | ⨁⨁◯◯ Low | CRITICAL |
| 5a) Delayed detection of HIV (e.g. number of early or acute infections detected and/or missed in RDT/ST algorithms vs RNA-based algorithms) (Initiation, CAB + LEN) | | | | | | | | | | | | |
| 5^10^ | non-randomised studies | serious^l^ | not serious | serious^b^ | not serious^f^ | none | 16/9033 (0.2%) | 0/9033 (0.0%) | **OR 7.08** (1.87 to 26.87) | **0 fewer per 1,000** (from 0 fewer to 0 fewer) | ⨁⨁◯◯ Low | CRITICAL |
| 5b) Delayed detection of HIV (e.g. number of early or acute infections detected and/or missed in RDT/ST algorithms vs RNA-based algorithms) (Initiation, CAB) | | | | | | | | | | | | |
| 3^7,11^ | non-randomised studies | serious^l^ | not serious | serious^b^ | not serious^f^ | none | 8/4720 (0.2%) | 0/4720 (0.0%) | **OR 5.96** (1.04 to 34.15) | **0 fewer per 1,000** (from 0 fewer to 0 fewer) | ⨁⨁◯◯ Low | CRITICAL |
| 5c) Delayed detection of HIV (e.g. number of early or acute infections detected and/or missed in RDT/ST algorithms vs RNA-based algorithms) (Initiation, LEN) | | | | | | | | | | | | |
| 2^8^ | non-randomised studies | very serious^l,m^ | not serious | serious^b^ | not serious^f^ | none | 8/4313 (0.2%) | 0/4313 (0.0%) | **OR 9.02** (1.14 to 71.20) | **0 fewer per 1,000** (from 0 fewer to 0 fewer) | ⨁◯◯◯ Very low | CRITICAL |
| 5d) Delayed detection of HIV (e.g. number of early or acute infections detected and/or missed in RDT/ST algorithms vs RNA-based algorithms) (Continuation, CAB) | | | | | | | | | | | | |
| 2^7^ | non-randomised studies | serious^l^ | not serious | serious^b^ | not serious^f^ | none | 11/3858 (0.3%) | 0/3858 (0.0%) | **OR 23.11** (1.36 to 392.46) | **0 fewer per 1,000** (from 0 fewer to 0 fewer) | ⨁⨁◯◯ Low | CRITICAL |
| 6a) Resistance-associated mutations detected at first evidence of HIV (e.g. number and proportion of mutations reported out of all those tested in RDT/ST algorithms vs RNA-based algorithms) (CAB + LEN) | | | | | | | | | | | | |
| 4^6^ | non-randomised studies | serious^n^ | not serious | serious^b^ | not serious^f^ | none | Major INSTI RAMs were detected in 10/3858 (0.3%) compared with TDF/FTC 0/3858 (0%, p-value 0.002). Accessory INSTI RAMs were detected in 15/3858 (0.4%) compared with TDF/FTC 28/3858 (0.7%, p-value 0.07). From HPTN 083, for 8/10 cases, RNA testing would have detected the HIV infection before INSTI RAMs emerged. Data from PURPOSE 1 and PURPOSE 2 reported Capsid RAM in 2/4313 (0.05%) for LEN users cf. 0/2154 (0%) for TDF/FTC users and 0/2136 (0%) for TAF/FTC users. | | | | ⨁⨁◯◯ Low | CRITICAL |
| 6b) Resistance-associated mutations detected at first evidence of HIV infection (e.g. number and proportion of mutations reported out of all those tested in RDT/ST algorithms vs RNA-based algorithms) (CAB) | | | | | | | | | | | | |
| 2^7^ | non-randomised studies | serious^n^ | not serious | serious^b^ | not serious^f^ | none | Major INSTI RAMs were detected in 10/3858 (0.3%) of CAB users compared with 0/3858 of TDF/FTC users (p-value 0.002). Accessory INSTI RAMs were detected in 15/3858 (0.4%) of CAB users compared with 28/3858 (0.7%) of TDF/FTC users (p-value 0.07). Denominator is the number of individuals using CAB in HPTN 083 and HPTN 084. No direct data available on differences or ability to prevent resistance based on test type. | | | | ⨁⨁◯◯ Low | CRITICAL |
| 6c) Resistance-associated mutations detected at first evidence of HIV (e.g. number and proportion of mutations reported out of all those tested in RDT/ST algorithms vs RNA-based algorithms) (LEN) | | | | | | | | | | | | |
| 2^8^ | non-randomised studies | serious^n^ | not serious | serious^b^ | serious^o^ | none | No direct data available on differences or ability to prevent resistance based on test type. Capsid RAM was detected in 2/4313 (0.05%) of LEN users compared with 0/2154 (0%) of TDF/FTC users and 0/2136 (0%) of TAF/FTC users (p-value 0.50). Denominator is the number of individuals using LEN in PURPOSE-1 and PURPOSE-2. | | | | ⨁◯◯◯ Very low | CRITICAL |
| 7) Turnaround time of test results among participants starting, continuing or discontinuing LA-PrEP with RDT/ST strategies and algorithms vs RNA-based testing strategies and algorithms | | | | | | | | | | | | |
|  | | | | | | | | | | | | |
| 6^12,13^ | Personal communication / systematic review | serious^p^ | not serious | serious^q^ | not serious^r^ | none | Compared to same day consultation results with rapid test or 1-5 days for laboratory-based Ag/Ab tests, qualitative RNA tests can take 1 week. Indirect evidence from a systematic review of HIV testing for infants reported the median time between sample collection and result delivery to caregivers was 35 days [interquartile range: 35-37] for lab-based testing, compared to 0 days [0-1] for RDT. | | | | ⨁◯◯◯ Very low | CRITICAL |
| 8a) Testing Frequency (e.g. measured as the number of time points an individual is tested for HIV, may include additional testing services for STIs as well) (CAB + LEN) | | | | | | | | | | | | |
| 5^14^ | non-randomised studies | not serious | serious^s^ | serious^b^ | not serious | none | Based on 4 non-randomised comparator studies (HPTN 083, HPTN 084, PURPOSE-1, PURPOSE-2), the frequency of HIV testing would not change whether using an RDT-based strategy or NAT strategy. Testing is every 8 weeks for CAB users and every 13 weeks with LEN users in the studies. One study (SEARCH Dynamic Choice) used 3rd Gen RDT at weeks 0, 4, 8, 16, 24, 32, 40, 48, 56, 64, 72, 80, 88 and 96; and NAT at weeks 0, 24, 48, 72 and 96. | | | | ⨁⨁◯◯ Low | IMPORTANT |
| 8b) Testing Frequency (e.g. measured as the number of time points an individual is tested for HIV, may include additional testing services for STIs as well) (CAB + LEN) | | | | | | | | | | | | |
| 17^15,16,17,18^ | Observational cohorts /  case series/report | not serious | not serious | serious^b^ | not serious | none | For studies conducting RDT/Lab-based Ag/Ab test and a sample taken for NAT at a later point (ImPrEP CAB Brasil, Primo Malawi, Tshireletso, PrEP15-19 Choices, Project PrEP, CAB-PK), there was no difference in testing frequency of RDT + NAT. For studies conducting RDT/Lab-based Ag/Ab test and RNA test concurrently (SeroPrEP, US Case Report, CATALYST, FASTPrEP and Zambia program), there was no difference in testing frequency of RDT + NAT, except for SEARCH Dynamic Choice where 3rd Gen RDT was used at weeks 0, 4, 8, 16, 24, 32, 40, 48, 56, 64, 72, 80, 88 and 96; and NAT at weeks 0, 24, 48, 72 and 96. For studies only using RDT, the frequency of testing was every 2 months for MOBILE MEN and Malawi Path to Scale, every 2/3 months for AXIS, and every month for Zimbabwe observational cohort. | | | | ⨁◯◯◯ Very low | IMPORTANT |
| 8c) Testing Frequency (e.g. measured as the number of time points an individual is tested for HIV, may include additional testing services for STIs as well (CAB) | | | | | | | | | | | | |
| 4^19^ | Observational cohorts | not serious | serious^t^ | serious^u^ | not serious | none | There was no difference in testing frequency for two studies (Brazil PrEP 15-19: HIVST offered at each visit to evaluate test performance and blood-based HIVST offered to 50 participants for preference study; ImPrEP CAB Brasil: Zambia: HIVST temporarily used due to stockouts of RDT). There was increased testing time points for ImPrEP CAB Brasil where HIVST was recommended 24 hours before CAB injections and MOBILE MEN where HIVST was offered 2 weeks following CAB LA initiation. | | | | ⨁◯◯◯ Very low | IMPORTANT |
| 9) Sexual Risk Behaviour (e.g., measured as report of condomless sex, sexual transmitted infections, or number of sexual partners) | | | | | | | | | | | | |
| 0 |  |  |  |  |  |  | No data on sexual behaviours of those newly diagnosed, specifically if any delays caused by the HIV testing strategies resulted in any onward transmission. | | | | - | IMPORTANT |
| 10a) Clinical/social harm: number and proportion of testers who experienced social harm/adverse events (e.g., misdiagnosis) | | | | | | | | | | | | |
| 5^14^ | non-randomised studies | serious^v^ | not serious | serious^b^ | not serious | none | Five studies reported no clinical sequelae from using RDTs, lab-based Ag/Ab tests or NAT (i.e., consequences from misdiagnosis, including anxiety or domestic violence from false positives) in the testing procedure. | | | | ⨁⨁◯◯ Low | IMPORTANT |
| 10b) Clinical/social harm: number and proportion of testers who experienced social harm/adverse events (e.g., misdiagnosis) | | | | | | | | | | | | |
| 2^2,20^ | Case series/ report | serious^v^ | not serious | serious^b^ | not serious | none | One case series and one case report reported no clinical sequelae (i.e., consequences from misdiagnosis, including anxiety or domestic violence from false positives) in the testing procedure. | | | | ⨁◯◯◯ Very low | IMPORTANT |

Ag/Ab: Antigen/Antibody; CAB-LA: Long-acting Cabotegravir; CI: confidence interval; INSTI RAM: Integrase inhibitor resistance-associated mutation; LEN: Lenacapavir: NAT: Nucleic acid testing; NPV: Negative predictive value; OR: odds ratio; PPV: Positive predictive value; RDT: Rapid diagnostic test; ST: self-testing; TDF/FTC: Tenofovir disoproxil fumarate/emtricitabine; US: United States

#### Explanations

a. Risk of bias: Selection bias is possible from the case report, observational cohorts, and SeroPrEP program which is based on voluntary enrolment from CAB-LA users in the USA. No data is available from those who tested positive at pre-enrolment screening (227 positive screening in HPTN 083; 208 positive screening in HPTN 084; 511 positive screening in PURPOSE-1; and 348 positive screening in PURPOSE-2).

b. Indirectness: Study is not designed to directly compare the HIV testing strategies of RDT and NAT.

c. Imprecision: The number of events are too small (<50) to draw firm conclusions.

d. Risk of bias: No data is available from those using RDT strategy.

e. Risk of bias: Data not complete due to selection bias associated with pre-enrolment screening (227 positive screening in HPTN 083; 208 positive screening in HPTN 084; 511 positive screening in PURPOSE-1; and 348 positive screening in PURPOSE-2)

f. Imprecision: not marked down because of large denominators despite small number of events.

g. Risk of bias: Data not complete due to selection bias associated with pre-enrolment screening (227 positive screening in HPTN 083; and 208 positive screening in HPTN 084).

h. Risk of bias: Data not complete due to selection bias associated with pre-enrolment screening (511 positive screening in PURPOSE-1; and 348 positive screening in PURPOSE-2).

i. Risk of bias: Data not complete due to selection bias associated with pre-enrolment screening (227 positive screening in HPTN 083; 208 positive screening in HPTN 084; 511 positive screening in PURPOSE-1; and 348 positive screening in PURPOSE-2)

j. We calculated 95% confidence intervals for negative predictive value (NPV) using the Wilson score method for binomial proportions. This method is widely used for proportions as it provides accurate and reliable interval estimates, particularly in cases with moderate to high proportions, and it addresses limitations of simpler methods, such as the Wald method, which can yield inaccurate intervals for small sample sizes or extreme proportions

k. Risk of bias: Diagnostic performance only available for combined strategy of RDT + Lab-based Ag/Ab test. No data specifically for RDT available.

l. Risk of bias: Only those who tested positive with RDT had an RNA test conducted.

m. Risk of bias: Missing data of delayed time from 9 of 10 cases for lenacapavir.

n. Risk of bias: there is a risk of reporting bias as the ability to detect resistance-associated mutations will depend on the viral load at the first evidence of HIV.

o. Imprecision: only two individuals acquired HIV (PURPOSE-2)

p. Based on personal communication of likely turnaround time of test results (not actual patient data).

q. Indirectness: Indirect evidence from systematic review of HIV testing in infants. (Luo. Lancet 2022;400(10356):887-895)

r. Imprecision was mitigated by the use of indirect evidence from a systematic review of 164 studies (Luo et al. Lancet 2022;400(10356):887-895

s. Inconsistency: One study (SAPPHIRE Dynamic Choice) had less frequent NAT compared to RDT.

t. There was no difference in testing frequency for two studies (Brazil PrEP 15-19: HIVST offered at each visit to evaluate test performance and blood-based HIVST offered to 50 participants for preference study; ImPrEP CAB Brasil: Zambia: HIVST temporarily used due to stockouts of RDT). There was increased testing time points for ImPrEP CAB Brasil where HIVST was recommended 24 hours before CAB injections and MOBILE MEN where HIVST was offered 2 weeks following CAB LA initiation.

u. Indirectness: Study not designed to compare HIVST test frequency with RDT or NAT.

v. Risk of bias: There was no standardised way to measure social harms, specifically related to the type of testing strategies.

#### **References**

1 . Data from Zimbabwe observational cohort.

2. Koss, C. A., Gandhi, M., Halvas, E. K., Okochi, H., Chu, C., Glidden, D. V., Gomez, L. G., Heaps, A. L., Conroy, A. A., Tran, M., Shetler, C., Hoeth, D., Kuncze, K., Louie, A., Garza, H. R., Mugoma, E. W., Penrose, K. J., Chohan, B. H., Ayieko, J. O., Mills, A., Patel, R. R., Mellors, J. W., Parikh, U. M.. First Case of HIV Seroconversion With Integrase Resistance Mutations on Long-Acting Cabotegravir for Prevention in Routine Care. Open Forum Infectious Diseases; 2024.

3. Hazra, A., Landovitz, R., Marzinke, M, Quinby, C., Creticos, C. Breakthrough HIV-1 infection in setting of cabotegravir for HIV pre-exposure prophylaxis. AIDS 2023.

4. Data from CATALYST (WHO Survey).

5. Personal communication - Raphael Landovitz. Unpublished data from HPTN 083 Open Label Extension.

6. Data from HPTN 083, HPTN 084, PURPOSE-1 and PURPOSE-2.

7. Data from HPTN 083 and HPTN 084.

8. Data from PURPOSE-1 and PURPOSE-2.

9. Open Label Extension of HPTN 083 (AIDS 2024 presentation by Landovitz Abstract 12267).

10. Data from HPTN 083, HPTN 084, PURPOSE 1, PURPOSE 2 and FASTPrEP.

11. Data from HPTN 083, HPTN 084 and FASTPrEP.

12. Personal communication with Moupali Das for PURPOSE-1/2. Data from WHO survey (CATALYST, CAB-PK, Zambia observational cohort)

13. Luo, R., Fong, Y., Boeras, D., Jani, I., Vojnov, L. The clinical effect of point-of-care HIV diagnosis in infants: a systematic review and meta-analysis.Lancet; Sep 17 2022.

14. Data from HPTN 083, HPTN 084, PURPOSE 1, PURPOSE 2 and SEARCH dynamic choice.

15. Data from WHO survey: AXIS – Private pharmacies, CAB-PK, CATALYST, FASTPrEP, ImPrEP CAB Brasil, Malawi Path to Scale, MOBILE MEN, PrEP15-19 Choices, Project PrEP, Primo Malawi, SEARCH Dynamic Choice, Tshireletso, Zambia program and Zimbabwe observational cohort.

16. Zhu W, Huang Y-LA, Delaney KP, Patel R, Kourtis A, Hoover KW. Few Discordant HIV Ag/Ab and RNA Test Results Among Persons in a National Cohort of PrEP Users. CROI 2024.

17. Mayer KH, Frick AJ, Brown C, et al. Real-world use of cabotegravir long-acting for pre-exposure prophylaxis. Trio Health Cohort. CROI 2024.

18. Macdonald P. Use of HIV rapid detection tests when initiating long-acting cabotegravir for HIV prevention, within an implementation science project. HIVR4P 2024.

19. Data from Brazil PrEP 15-19, ImPrEP CAB Brasil, Zambia and MOBILE MEN.

20. Parikh UM, et al. Early Virologic Success on ART following Breakthrough Infection on CAB-LA PrEP. HIVR4P 2024 (Lima, Peru).
