# Supplementary material for: Rapid diagnostic tests, laboratory-based immunoassay and nucleic acid testing strategies for long-acting injectable pre-exposure prophylaxis: A systematic review and meta-analysis
Source: PLoS Med. 2026 Apr 16;23(4):e1005030. doi: 10.1371/journal.pmed.1005030 (PMC13102303; doi:10.1371/journal.pmed.1005030)
Supplement: S5 Appendix — (DOCX) [file pmed.1005030.s005.docx]

# S5 Appendix. Characteristics and testing strategies of each program

- **Table A. Characteristics and testing strategies for CAB-LA studies**

| **Country** | **Populations served** | **Studies** | **Testing strategy at the initiation** | | **Testing strategy at the continuation** | | **Testing strategy after resumption from hiatus** | |
| --- | --- | --- | --- | --- | --- | --- | --- | --- |
|  |  |  | **Non-RNA** | **RNA** | **Non-RNA** | **RNA** | **Non-RNA** | **RNA** |
| Brazil | Men who have sex with men, trans and gender diverse people (age 18-30 years) | ImPrEP CAB Brasil^1^   - Observational cohort | 3^rd^ and 4^th^ Gen RDT | Y | 3^rd^ and 4^th^ Gen RDT  (Blood-based HIVST 24 hrs before initiation encouraged) | If discordant result for RDT 1 and 2 | NI | NI |
|  | Adolescent men who have sex with men and trans gender women (age 15-19 years) | PrEP 15-19^1^   - Observational cohort | 4^th^ Gen RDTs and Oral HIVST | Y | 4^th^ Gen RDTs and HIVST | N | 4^th^ Gen RDTs and oral HIVST | Y |
| USA | Men who have sex with men, trans and gender diverse people | US (Case report)^2^ | Laboratory-based Ag/Ab | Y | Laboratory-based Ag/Ab | Y | NI | NI |
|  |  | SeroPrEP (Case series)^3,4^ | RDT (not specified) or laboratory-based Ag/Ab | Y | RDT (not specified) or laboratory-based Ag/Ab | Y | RDT (not specified) | N |
|  | Adolescent girls and young women, female sex workers, trans and gender diverse people | HealthVerity^5^   - Observational cohort | Laboratory-based Ag/Ab | Y | Laboratory-based Ag/Ab | Y | NI | NI |
|  | Not specified | Trio Health^6^   - Observational cohort | Laboratory-based Ag/Ab | Y | Laboratory-based Ag/Ab | Y | NI | NI |
| Countries in Sub-Saharan Africa | Females | HPTN 084^7-9^   - Non-randomised comparative study | RDT (not specified) and Laboratory-based Ag/Ab at enrolment | At eligibility screening and within 14 days prior to the enrolment | RDT (not specified) and Laboratory-based Ag/Ab | On stored samples if tested positive | RDT (not specified) and Laboratory-based Ag/Ab | On stored samples if tested positive |
|  | High risk young women and men: HIV neg people age 18-35 (women) and more than 18 (men) wanting PrEP | AXIS - Private pharmacies^1^   - Observational cohort | 3^rd^ Gen RDT | N | 3^rd^ Gen RDT | N | 3^rd^ Gen RDT | N |
|  | Males who travelled for work in the past 6 months | MOBILE MEN^1^   - Observational cohort | RDT (not specified) [HIVST at week 2] | N | RDT (not specified) | N | NI | NI |
|  | Men who have sex with men, female sex workers, serodiscordant couples, adolescent girls and young women | Zimbabwe (real-world)^1^   - Observational cohort | 3^rd^ Gen RDT | N | 3^rd^ Gen RDT | N | NI | NI |
|  | Men who have sex with men, trans and gender diverse people | SEARCH Dynamic choice^10^   - Non-randomised comparative study | HIVST or RDT (not specified) | Y | HIVST or RDT (not specified) | Y (week 24-48 weeks) | NI | NI |
|  | Pregnant and post-partum people | CAB-PK^1^   - Observational cohort | 3^rd^ Gen RDT | Y | 3^rd^ Gen RDT | N | NI | NI |
|  |  | Tshireletso^1^   - Observational cohort | 4^th^ Gen RDT | On stored sample if tested positive of if 4^th^ Gen RDT not available | 4^th^ Gen RDT | On stored sample if tested positive | NI | NI |
|  |  | Primo Malawi^1^   - Observational cohort | 3^rd^ Gen RDT | If tested positive | 3^rd^ Gen RDT | N | NI | NI |
|  | Young people (age 15-29) | FASTPrEP^1,11^   - Observational cohort | 3^rd^ and 4^th^ Gen RDTs | Y | 3^rd^ or 4^th^ RDT | Y according to the study schedule (weeks 0, 4, 28) or if tested positive | NI | NI |
|  | Not specified | CATALYST^1^   - Non-randomised comparative study | 3^rd^ or 4^th^ Gen RDTs | Y | 3^rd^ or 4^th^ RDT | Y (every 6 months on stored samples) | NI | NI |
|  |  | Malawi path to scale^1^   - Observational cohort | 3^rd^ Gen RDT | N | 3^rd^ Gen RDT | N | NI | NI |
|  |  | Project PrEP^1^   - Observational cohort | 3^rd^ Gen RDT | Y | 3^rd^ Gen RDT | If tested positive | NI | NI |
|  |  | Zambia Program^1^   - Observational cohort | 3^rd^ Gen RDT + HIVST | Y but results not necessary for initiation | 3^rd^ Gen RDT + HIVST | Y but results not necessary for continuation | NI | NI |
| Multiple countries globally | Men who have sex with men, trans and gender diverse people | HPTN 083^9,12-18^   - Non-randomised comparative study | RDT (not specified) and Laboratory –based Ag/Ab at enrolment | At eligibility screening and within 14 days prior to the enrolment | RDT (not specified) and Laboratory-based Ag/Ab | On stored sample if tested positive | RDT (not specified) and Laboratory-based Ag/Ab | On stored sample if tested positive |

- **Table B. Characteristics and testing strategies for Lenacapavir studies**

| **Country** | **Populations served** | **Studies** | **Testing strategy at the initiation** | | **Testing strategy at the continuation** | | **Testing strategy after resumption from hiatus** | |
| --- | --- | --- | --- | --- | --- | --- | --- | --- |
|  |  |  | **Non-RNA** | **RNA** | **Non-RNA** | **RNA** | **Non-RNA** | **RNA** |
| Countries in Sub-Saharan Africa | Adolescent girls and young women (age 16-25) | PURPOSE 1^19,20^   - Non-randomised comparative study | 4^th^ Gen RDT or Laboratory-based Ag/Ab | At eligibility screening and enrolment | 4^th^ Gen RDT and Laboratory-based Ag/Ab | On stored sample if tested positive | 4^th^ Gen RDT | If tested positive |
| Multiple countries globally | Men who have sex with men, Trans and gender diverse people | PURPOSE 2^21,22^   - Non-randomised comparative study | 4^th^ Gen RDT or Laboratory-based Ag/Ab | At eligibility screening and enrolment | 4^th^ Gen RDT and Laboratory-based Ag/Ab | On stored sample if tested positive | 4^th^ Gen RDT | If tested positive |

Ag/Ab=antigen/antibody; CAB-LA=Long-acting cabotegravir; HIVST=HIV self-testing; Gen=Generation; NI=No information available; N=no; PrEP=pre-exposure prophylaxis; RDT=rapid diagnostic test; WHO=World Health Organization; Y=Yes

Young people include adolescent girls and young women, pregnant and breastfeeding women, men who have sex with men and male partners

**Reference**

1. WHO open call. CAB-LA implementation program/study/trial.

2. Hazra A, Landovitz RJ, Marzinke MA, Quinby C, Creticos C. Breakthrough HIV-1 infection in setting of cabotegravir for HIV pre-exposure prophylaxis. *AIDS* 2023; **37**(11): 1711–4.

3. Koss CA, Gandhi M, Halvas EK, et al. First case of HIV seroconversion with integrase resistance mutations on long-acting cabotegravir for prevention in routine care. *Open Forum Infect Dis* 2024; **11**(9): ofae468.

4. Parikh UM, Altamirano J, Safa H, et al. Early Virologic Success on ART following Breakthrough Infection on CAB-LA PrEP. HIVR4P2024. Lima, Peru.

5. Zhu W, Huang Y-LA, Delaney KP, Patel R, Kourtis A, Hoover KW. Few Discordant HIV Ag/Ab and RNA Test Results Among Persons in a National Cohort of PrEP Users. CROI2024.

6. Mayer KH, Frick AJ, Brown C, et al. Real-world use of cabotegravir long-acting for pre-exposure prophylaxis. Trio Health Cohort. CROI2024; 2024.

7. Delany-Moretlwe S, Hughes JP, Bock P, et al. Cabotegravir for the prevention of HIV-1 in women: results from HPTN 084, a phase 3, randomised clinical trial. *Lancet* 2022; **399**(10337): 1779–89.

8. Eshleman SH, Fogel JM, Piwowar-Manning E, et al. Characterization of Human Immunodeficiency Virus (HIV) Infections in Women Who Received Injectable Cabotegravir or Tenofovir Disoproxil Fumarate/Emtricitabine for HIV Prevention: HPTN 084. *The Journal of infectious diseases* 2022; **225**(10): 1741–9.

9. Jamieson L, Johnson LF, Nichols BE, et al. Relative cost-effectiveness of long-acting injectable cabotegravir versus oral pre-exposure prophylaxis in South Africa based on the HPTN 083 and HPTN 084 trials: a modelled economic evaluation and threshold analysis. *Lancet HIV* 2022; **9**(12): e857–e67.

10. Kamya MR, Balzer LB, Ayieko J, et al. Dynamic choice HIV prevention with cabotegravir long-acting injectable in rural Uganda and Kenya: a randomised trial extension. *The lancet HIV* 2024.

11. Macdonald P. Use of HIV rapid detection tests when initiating long-acting cabotegravir for HIV prevention, within an implementation science project. HIVR4P2024.

12. Eshleman SH, Fogel JM, Halvas EK, et al. HIV RNA Screening Reduces Integrase Strand Transfer Inhibitor Resistance Risk in Persons Receiving Long-Acting Cabotegravir for HIV Prevention. *The Journal of infectious diseases* 2022; **226**(12): 2170–80.

13. Fogel JM, Piwowar-Manning E, Moser A, et al. Evaluation of Xpert point-of-care assays for detection of HIV infection in persons using long-acting cabotegravir for pre-exposure prophylaxis. *Microbiology spectrum* 2024: e0030724.

14. Landovitz RJ, Donnell D, Clement ME, et al. Cabotegravir for HIV Prevention in Cisgender Men and Transgender Women. *The New England journal of medicine* 2021; **385**(7): 595–608.

15. Landovitz RJ, Hanscom BS, Clement ME, et al. Efficacy and safety of long-acting cabotegravir compared with daily oral tenofovir disoproxil fumarate plus emtricitabine to prevent HIV infection in cisgender men and transgender women who have sex with men 1 year after study unblinding: a secondary analysis of the phase 2b and 3 HPTN 083 randomised controlled trial. *The Lancet HIV* 2023; **10**(12): e767–e78.

16. Marzinke MA, Fogel JM, Wang Z, et al. Extended Analysis of HIV Infection in Cisgender Men and Transgender Women Who Have Sex with Men Receiving Injectable Cabotegravir for HIV Prevention: HPTN 083. *Antimicrobial agents and chemotherapy* 2023; **67**(4): e0005323.

17. Marzinke MA, Grinsztejn B, Fogel JM, et al. Characterization of Human Immunodeficiency Virus (HIV) Infection in Cisgender Men and Transgender Women Who Have Sex With Men Receiving Injectable Cabotegravir for HIV Prevention: HPTN 083. *The Journal of infectious diseases* 2021; **224**(9): 1581–92.

18. Landovitz RJ, Gao F, Fogel JM, et al. Performance characteristics of HIV RNA screening with CAB-LA PrEP in HPTN 083. AIDS2024. Munich, Germany; 2024.

19. Bekker L-G, Das M, Abdool Karim Q, et al. Twice-Yearly Lenacapavir or Daily F/TAF for HIV Prevention in Cisgender Women. *The New England journal of medicine* 2024.

20. Bekker L-G, Kiwanuka N, Selepe P, et al. Annual Persistence in Use of Twice-Yearly Lenacapavir Versus Daily Oral PrEP in the PURPOSE 1 Phase 3 Trial. HIV Glasgow2024. Glasgow, United Kingdom.

21. Kelley CF, Acevedo-Quiñones M, Agwu AL, et al. Twice-Yearly Lenacapavir for HIV Prevention in Men and Gender-Diverse Persons. *N Engl J Med* 2024.

22. Ogbuagu O. Twice-Yearly Lenecapavir PrEP in Cisgender Gay, Bisexual, and Other Men, Transgender Women and Men, and Gender-Diverse People (PURPOSE 2). HIV Drug Therapy Glasgow 2024. Glasgow, United Kingdom.
