# Supplementary material for: Rapid diagnostic tests, laboratory-based immunoassay and nucleic acid testing strategies for long-acting injectable pre-exposure prophylaxis: A systematic review and meta-analysis
Source: PLoS Med. 2026 Apr 16;23(4):e1005030. doi: 10.1371/journal.pmed.1005030 (PMC13102303; doi:10.1371/journal.pmed.1005030)
Supplement: S6 Appendix — (DOCX) [file pmed.1005030.s006.docx]

# S6 Appendix. Time to linkage to care

**Table A. Time to linkage to care**

| **Studies** | **Testing strategy** | **Number of cases** | **Time to linkage or ART initiation** |
| --- | --- | --- | --- |
| Zimbabwe (Observational cohort) | 3rd Gen RDT  HIV-1/2 Determine (Oraquick)  HIV 1/2-STAT-PACK (Chembio)  HIV-1/2 antibody test (INSTI) | 1 CAB-LA user  9 Dapivirine ring users | 1 day to initiate ART |
| A case report from the US | Lab test  (4^th^ Gen Ag/Ab test - Quest Diagnostics) & RNA test | 1 CAB-LA user | 9 days to initiate ART |
| SeroPrEP (Observational cohort) | Lab test  (5^th^ Gen Ag/Ab test - Bioplex 2200 HIV Ag/Ab Multiplex Flow) & RNA (Hologic Aptima Quant Dx) | 1 CAB-LA user | 6 days to initiate ART |
| CATALYST | RDT (3rd or 4th Gen RDT with no brand mentioned) & RNA test | 2 people at CAB-initiation | Case 1 returned 8-days post-injection  Case 2 returned 21-days post-injection. No data on ART initiation available yet |

Ab=antibody, Ag=antigen, ART=antiretroviral therapy, CAB-LA= long-acting cabotegravir, Gen=Generation, LAI-PrEP = long-acting injectable PrEP, RDT=rapid diagnostic test
