# Supplementary material for: Rapid diagnostic tests, laboratory-based immunoassay and nucleic acid testing strategies for long-acting injectable pre-exposure prophylaxis: A systematic review and meta-analysis
Source: PLoS Med. 2026 Apr 16;23(4):e1005030. doi: 10.1371/journal.pmed.1005030 (PMC13102303; doi:10.1371/journal.pmed.1005030)
Supplement: S7 Appendix — (DOCX) [file pmed.1005030.s007.docx]

# S7 Appendix. PrEP holds or discontinuation from false positive

In **HPTN 083** Open Label Extension,

- 22/2483 (0.9%) received false positive RNA tests.
  - 4 delayed long-acting cabotegravir (CAB-LA) initiation (>70 days)
  - 2 discontinued CAB-LA
  - 1 oral CAB gap during the 5-week oral CAB lead-in phase (26-day gap)
- 20/2483 (0.8%) received false positive RDT + laboratory-based immunoassay tests.
  - No further data available
  - Extra tests to confirm HIV diagnosis would be similar to above

In **CATALYST**,

- 2/1010 (0.2%) received false positive RNA tests
  - Both decided to continue with their CAB-LA injection
