# Supplementary material for: Rapid diagnostic tests, laboratory-based immunoassay and nucleic acid testing strategies for long-acting injectable pre-exposure prophylaxis: A systematic review and meta-analysis
Source: PLoS Med. 2026 Apr 16;23(4):e1005030. doi: 10.1371/journal.pmed.1005030 (PMC13102303; doi:10.1371/journal.pmed.1005030)
Supplement: S8 Appendix — (DOCX) [file pmed.1005030.s008.docx]

# S8 Appendix. HIV positivity

- **Table A. HIV positivity among CAB-LA studies**

| **Program** | **Overall** | | | **Baseline** | | | | | **Continuation** | | | | |
| --- | --- | --- | --- | --- | --- | --- | --- | --- | --- | --- | --- | --- | --- |
|  | **People assigned to LAI-PrEP arm** | **All HIV positive in the LAI-PrEP arm** | **HIV positive while using LAI-PrEP** | **Test used** | **HIV positive detected by RNA tests** | **HIV positive detected by RDT tests** | **HIV positive detected by laboratory-based immunoassay** | **Tests used** | | **HIV positive detected by RNA tests** | **HIV positive detected by RDT tests** | **HIV positive detected by laboratory-based immunoassay** |  |
| HPTN 083 | 2244 | 34 | 11 | - RNA tests for eligibility screening - RDT (no further details) and laboratory-based immunoassay at the enrollment | 4/4  (1 didn't receive CAB-LA) | 0/4 | 1/4  (Ag/Ab lab test)  0/1  (Ab lab test) | - RDT (no further details) and Lab Ag/Ab test - If positive – RNA on the stored sample | | Total 11/11, of which   - 6 (on-time injection) - 3 (delayed CAB-injection >70days) - 2 (restart CAB LA) | 8/11  *This number does not include 1st HIV positives during the oral lead-in phase | 10/11  *This number not included 1st HIV positives during the oral lead-in phase |  |
| HPTN 084 | 1614 | 4 | 1 | - RNA tests for eligibility screening - RDT (no further details) and laboratory-based immunoassay at the enrollment | 1/1 | 0/1 | 1/1 | - RDT(no further details) and laboratory-based immunoassay - If positive - NAT on the stored sample | | 1/1 | 0/1 | 1/1 |  |
| SeroPrEP | Unclear | 4 | 4 | - RNA tests | NA | NA | NA | - [RDT (no further details) or laboratory-based immunoassay] AND RNA tests | | 4/4 | 0/3 | 2/4 |  |
| CATALYST | 1010 | 5 | Not available yet | - 3rd or 4th Gen RDT and RNA tests | 3/3 | 1/3 | NA | - 3rd or 4th Gen RDT and [RNA every 6 months] | | Not available yet | Not available yet | NA |  |
| Zimbabwe (observational cohort) | 1772 | 1 | 1 | - 3rd Gen RDT | NA | NA | NA | - 3rd Gen RDT | | NA | 1/1 | NA |  |

Ab=antibody, Ag=antigen, CAB-LA= long-acting cabotegravir, LAI-PrEP = long-acting injectable PrEP, NA=not available, RDT=rapid diagnostic test

- **Table B. HIV positivity among LEN studies**

| **Program** | **Overall** | | | **Baseline** | | | | **Continuation** | | | |
| --- | --- | --- | --- | --- | --- | --- | --- | --- | --- | --- | --- |
|  | **People assigned to LAI-PrEP arm** | **All HIV positive in the LAI-PrEP arm** | **HIV positive while using LAI-PrEP** | **Test used** | **People assigned to LAI-PrEP arm** | **All HIV positive in the LAI-PrEP arm** | **HIV positive while using LAI-PrEP** | **Test used** | **People assigned to LAI-PrEP arm** | **All HIV positive in the LAI-PrEP arm** | **HIV positive while using LAI-PrEP** |
| PURPOSE 1 | 2134 | 4 | 0 | - RNA tests for eligibility - [4th Gen RDT or laboratory-based immunoassay] AND RNA tests at the enrollment | 4/4 | 0/4 | 3/4 | - 4th Gen RDT and laboratory-based immunoassay - If positive - NAT on the stored sample | NA | NA | NA |
| PURPOSE 2 | 2179 | 6 | 2 | - RNA tests for eligibility - [4th Gen RDT or laboratory-based immunoassay] AND RNA tests at the enrollment | 4/4 | 0/4 | 2/4 | - 4th Gen RDT and laboratory-based immunoassay If positive - NAT on the stored sample | 2/2 | 1/2 | 2/2 |

LAI-PrEP=long-acting injectable PrEP, NAT=nucleic acid test, RDT=rapid diagnostic test, RNA=ribonucleic acid

**Table C. Studies with no HIV positivity**

| **Type of PrEP** | **Program** | **Type of test used** | **People assigned to LAI-PrEP arm** |  |
| --- | --- | --- | --- | --- |
|  |  |  |  |  |
| **CAB-LA** | AXIS | 3rd Gen RDT | 200 |  |
|  | ImPrEP CAB Brasil | 3rd Gen RDT and laboratory-based immunoassay [HIVST was available but not part of the testing algorithm] | 1200 |  |
|  | MOBILE MEN | RDT (No further details), HIVST (once and optional) | 400 |  |
|  | SEARCH Dynamic Choice | NAT test, 3rd Gen RDT, HIVST (optional) | 274 |  |

CAB-LA= long-acting cabotegravir, HIVST= HIV self-testing, LAI-PrEP = long-acting injectable pre-exposure prophylaxis, PrEP=pre-exposure prophylaxis, RDT=rapid diagnostic test

- **On-going studies with no HIV positivity data available yet**
  - CAB-PK
  - FASTPrEP
  - Heath Verity Cohort
  - Malawi Path to Scale
  - PrEP 15-19
  - Primo Malawi
  - Project PrEP
  - Trio Health Cohort
  - Tshieletso
  - Zambia Program
