# Supplementary material for: Rapid diagnostic tests, laboratory-based immunoassay and nucleic acid testing strategies for long-acting injectable pre-exposure prophylaxis: A systematic review and meta-analysis
Source: PLoS Med. 2026 Apr 16;23(4):e1005030. doi: 10.1371/journal.pmed.1005030 (PMC13102303; doi:10.1371/journal.pmed.1005030)
Supplement: S9 Appendix — (DOCX) [file pmed.1005030.s009.docx]

# S9 Appendix. Diagnostic accuracy and performance

- **Table A. Diagnostic accuracy (Initiation)**

| **Study programs** | **Diagnostic accuracy at initiation**  **(RDT+laboratory-based immunoassay algorithms compared to NAT)** |
| --- | --- |
| **Long-acting cabotegravir** | |
| HPTN 083 | **RCT Blinded phase initiation:** (negative NAT in 14 days prior to enrolment)   - **RDT**: 4/2282 false negative, 2278/2282 true negative, 0 false positive, 0 true positive (NPV 99.8%, 99.6-99.9%) - **Laboratory-based immunoassay**:3/2282 false negative, 2278/2282 true negative, 0 false positive, 1 true positive (NPV 99.9%, 99.6-100%)   - 4 enrolment cases diagnosed by laboratory-based immunoassay at first positive site visit, RDT (no Gen reported) negative   ***Data regarding positives not complete as 227 reactive at screening HIV test at enrolment;*** |
| HPTN 084 | **RCT Blinded phase initiation:** (negative NAT in 14 days prior to enrolment)   - **RDT**: 1/1614 false negative, 1613/1614 true negative, 0 false positive, 0 true negative (NPV 99.9%, 99.7-100%) - **Laboratory-based immunoassay**: 1/1614 false negative, 1613/1614 true negative, 0 true positive, 0 false positive (NPV 99.9%, 99.7-100%)   - 1 enrolment case diagnosed by laboratory-based immunoassay at first positive site visit, RDT (No Gen reported) negative   ***Data regarding positives not complete as 208 reactive screening HIV test at enrolment*** |
| FASTPrEP | **Programmatic data initiation tests:** 1254 tests performed in 862 CAB-LA initiators (196 4^th^ gen RDT, 274 3^rd^ gen RDT, 784 both RDTs)   - **3^rd^ gen RDT**: 2/666 false negative, 664/666 true negative (NPV 99.7%, 98.9-100%) - **4^th^ gen RDT**: 1/588 false negative, 587/588 true negative (NPV 99.8%, 99.0-100%)   **Data regarding positive results not available, so unable to calculate Sn, PPV** |
| CATALYST | **Pre-publication results from WHO survey - retrospective RNA testing at initiation:**   - **3rd or 4th Gen RDT**: 2/899 false negative, 897/899 true negative, (NPV 99.8%, 99.2-100%)   ***Positive data not available so the number of cases diagnosed using RDT not described*** |
| FASTPrEP | **Pre-publication results from WHO survey - retrospective RNA testing at initiation:**   - **3rd Gen RDT**: 2/862 false negative, 398/862 true negative, (NPV 99.5%, 98.2-99.9%)   ***Positive data not available so the number of cases diagnosed using RDT not described*** |
| **Lenecapavir** | |
| PURPOSE 1 | **RCT Blinded Phase Initiation**   - **4th Gen RDT**: 4/2138 false negative, 2134/2138 true negative, 0 false positive, 0 true positive (NPV 99.8%; 99.5-99.9%) - **Laboratory-based immunoassay**: 1/2138 false negative, 2134/2138 true negative, 0 false positive, 3 true positive (NPV 100%, 99.7-100%)   ***Data regarding positives not complete as 511 had positive screening HIV test pre-enrolment*** |
| PURPOSE 2 | **RCT Blinded Phase Initiation**   - **4th Gen RDT**: 4/2183 false negative, 2179/2183 true negative, 0 false positive, 0 true positives (NPV 99.8; 99.5-99.9%) - **Laboratory-based immunoassay**: 2/2183 false negative, 2179/2183 true negative, 0 false positive, 2 true positives (NPV 99.9%; 99.7-100%)   ***Data regarding positives not complete as 348 positive screening HIV test pre-enrolment*** |

CAB-LA= long-acting cabotegravir, Gen=Generation, NAT= nucleic acid test, NPV= negative predictive value, RCT= randomized controlled trial, RDT= rapid diagnostic test

- **Table B. Diagnostic accuracy (Continuation)**

Data from HPTN 083 Open-label extension

| **Test** | **True positive** | **False positive** | **True Negative** | **False Negative** | **Sensitivity** | **Specificity** | **Positive predictive value** | **Negative predictive value** |
| --- | --- | --- | --- | --- | --- | --- | --- | --- |
| **RDT (not specified) + Laboratory-based immunoassay** | 24 | 20 | 26477 | 5 | 82.8%  (65.5-92.4%) | 99.9%  (99.9-100%) | 54.6%  (40.1-68.3%) | 100% (100-100%) |
| **RNA Nucleic acid test** | 29 | 22 | 26475 | 0 | 100% (88.3-100%) | 99.9  (99.9-100%) | 56.9% (43.3-69.5%) | 100%  (100-100%) |

RDT=Rapid diagnostic test, RNA=Ribonucleic acid
