# Supplementary material for: Rapid diagnostic tests, laboratory-based immunoassay and nucleic acid testing strategies for long-acting injectable pre-exposure prophylaxis: A systematic review and meta-analysis
Source: PLoS Med. 2026 Apr 16;23(4):e1005030. doi: 10.1371/journal.pmed.1005030 (PMC13102303; doi:10.1371/journal.pmed.1005030)
Supplement: S10 Appendix — (DOCX) [file pmed.1005030.s010.docx]

# S10 Appendix. Turnaround time for results

**Table A. Turnaround time for results**

|  | **Rapid diagnostic test** | **Lab-based Ag/Ab test** | **Nucleic acid test** |
| --- | --- | --- | --- |
| CATALYST | Same-day | Not done | 2-4 weeks |
| CAB-PK | Same-day | No data | 2 days |
| Zambia | Same-day | Not done | 7 days |
| PURPOSE 1 / 2 | Same-day | 3-5 days | 1 week |

Ab=Antibody, Ag=Antigen
