# Supplementary material for: Rapid diagnostic tests, laboratory-based immunoassay and nucleic acid testing strategies for long-acting injectable pre-exposure prophylaxis: A systematic review and meta-analysis
Source: PLoS Med. 2026 Apr 16;23(4):e1005030. doi: 10.1371/journal.pmed.1005030 (PMC13102303; doi:10.1371/journal.pmed.1005030)
Supplement: S11 Appendix — (DOCX) [file pmed.1005030.s011.docx]

# S11 Appendix. Resistance-associated mutations

- **Table A. Resistance-associated mutation in the CAB-LA studies**

| **Study programs** | **LAI-PrEP arm** | **Comparator arm** |
| --- | --- | --- |
| HPTN083 | **LAI-PrEP: CAB-LA**  Major INSTI RAMs detected in 10/32 cases (31%).  Major RAMs included E138E/K, G140A/G/S, Q148R, N155H, R263K.  Accessory INSTI RAMs detected in 15/32 (47%) cases in CAB-LA arm | **Comparator: TDF/FTC**  Major INSTI RAMs were not detected in the TDF/FTC arm, 0/76 cases (0%)  Accessory INSTI RAMs were detected in 28/76 (37%) cases in TDF/FTC arm |
| HPTN084 | **LAI-PrEP: CAB-LA**  Major INSTI RAMs not detected (0/4 cases)  INSTI polymorphism detected in 1/4 (25%) cases | **Comparator TDF/FTC**  Major INSTI RAMs not detected (0/33 cases)  INSTI polymorphisms detected in 10/33 (30.3%) cases |
| SeroPrEP  (case report + case series) | **LAI-PrEP: CAB-LA**  **Case report:** Major RAM Q148R (2/24 sequences) and accessory RAM A128T (1/24 sequences) identified on Integrase single genome sequencing  **Case series:** 0/3 INSTI RAMs by Sanger  Major INSTI RAMs by single genome sequencing in 2/2 available results (E138K, 1/56 sequences case B); Q148R, 10/33 sequences case C) and 1/2 accessory RAMs (N155K, 1/43 sequences case B) | **N/A** |

CAB-LA: Long-acting Cabotegravir; INSTI RAM: Integrase inhibitor resistance-associated mutation; LAI-PrEP: long-acting injectable pre-exposure prophylaxis; TDF/FTC: Tenofovir disoproxil fumarate/emtricitabine

- **Table B. Resistance-associated mutation in the Lenacapavir studies**

| **Study programs** | **LAI-PrEP arm** | **Comparator arm** |
| --- | --- | --- |
| PURPOSE-1 | **LAI-PrEP: Lenacapavir**  No on-treatment infections | **Comparator: TDF/FTC and TAF/FTC**  Capsid RAMs not reported (0/16 TDF/FTC and 0/39 TAF/FTC) |
| PURPOSE-2 | **LAI-PrEP: Lenacapavir**  Capsid RAM detected in 2/2 (100%) on-treatment infections;  Mutation N74D in 2/2 cases | **Comparator: TDF/FTC**  Capsid RAMs not reported (0/9 TDF/FTC) |

RAM: resistance-associated mutation; LAI-PrEP: long-acting injectable pre-exposure prophylaxis; TDF/FTC: Tenofovir disoproxil fumarate/emtricitabine
