# Supplementary material for: Rapid diagnostic tests, laboratory-based immunoassay and nucleic acid testing strategies for long-acting injectable pre-exposure prophylaxis: A systematic review and meta-analysis
Source: PLoS Med. 2026 Apr 16;23(4):e1005030. doi: 10.1371/journal.pmed.1005030 (PMC13102303; doi:10.1371/journal.pmed.1005030)
Supplement: S12 Appendix — (DOCX) [file pmed.1005030.s012.docx]

# S12 Appendix. Costs of HIV tests (USD 2024)*

Table A. Cost of HIV test

| **Country** | **NAT** | **Laboratory-based Immunoassay** | **RDT** | **HIVST** |
| --- | --- | --- | --- | --- |
| USA^1^ | 85.10 (90.47) | 24.08  (25.60) |  |  |
| South Africa^2^ |  | 4.12 (4.51) | 0.52 (0.57) |  |
| South Africa^3^ | 36.00 (46.38) | 1.20 (1.55) |  |  |
| Brazil^4^ | 8.80 |  | 0.17 |  |
| Kenya^5^ | 22 (24.07) | 1.21 (1.32) |  | 3 (3.28 Oral)  5 (5.47 Blood) |
| Sub-Saharan Africa^6^ | 22 (23.39) |  | 4 (4.25 Ab only) |  |

* Costs were inflated to USD 2024 using IMF Customer Price Index^7^

HIVST = HIV self-testing; NAT = nucleic acid test; RDT = rapid diagnostic test; USD = United States dollar

**Reference**

1. Brogan AJ, Davis AE, Mellott CE, Fraysse J, Metzner AA, Oglesby AK. Cost-effectiveness of cabotegravir long-acting for HIV pre-exposure prophylaxis in the United States. *PharmacoEconomics* 2024; **42**(4): 447–61.

2. Jamieson L, Johnson LF, Nichols BE, et al. Relative cost-effectiveness of long-acting injectable cabotegravir versus oral pre-exposure prophylaxis in South Africa based on the HPTN 083 and HPTN 084 trials: a modelled economic evaluation and threshold analysis. *Lancet HIV* 2022; **9**(12): e857–e67.

3. Walensky RP, Jacobsen MM, Bekker LG, et al. Potential clinical and economic value of long-acting preexposure prophylaxis for south african women at high-risk for HIV infection. *J Infect Dis* 2016; **213**(10): 1523–31.

4. Paranhos J, Castilho M, Klein HE, et al. Costs of implementing long-acting injectable cabotegravir as HIV prophylaxis in Brazil. *Physis* 2023; **33**: e33021.

5. Cox SN, Wu L, Wittenauer R, et al. Impact of HIV self-testing for oral pre-exposure prophylaxis scale-up on drug resistance and HIV outcomes in western Kenya: a modelling study. *Lancet HIV* 2024; **11**(3): e167–e75.

6. Smith J, Bansi-Matharu L, Cambiano V, et al. Predicted effects of the introduction of long-acting injectable cabotegravir pre-exposure prophylaxis in sub-Saharan Africa: a modelling study. *Lancet HIV* 2023; **10**(4): e254–e65.

7. International Monetary Fund (IMF). Consumer Price Index (CPI).
