# Supplementary material for: Rapid diagnostic tests, laboratory-based immunoassay and nucleic acid testing strategies for long-acting injectable pre-exposure prophylaxis: A systematic review and meta-analysis
Source: PLoS Med. 2026 Apr 16;23(4):e1005030. doi: 10.1371/journal.pmed.1005030 (PMC13102303; doi:10.1371/journal.pmed.1005030)
Supplement: Attachment — Submitted filename: Response to Reviewers v3 25Dec11.docx [file pmed.1005030.s016.docx]

Re: "Balancing Accuracy, Accessibility, and Cost: HIV Testing Strategies for Long-Acting Injectable Pre-Exposure Prophylaxis: A Systematic Review of Rapid, Laboratory-Based Immunoassay and Nucleic Acid Testing Strategies" (PMEDICINE-D-25-02056R1)

December 11^th^, 2025

Dear Dr Farrell,

Thank you for the opportunity to revise and improve our manuscript. We have carefully addressed the editors' and reviewers’ comments point-by-point. Please find our responses and corresponding changes below. Additionally, we formatted the manuscript according to the journal's guidelines for authors. Do let us know if any further modifications are required.

Best regards,

Prof. Jason and Warittha, on behalf of the co-authors

**Comments from the academic editor:**

**1. The authors present the use of RDTs in an overly positive light without adequately acknowledging the substantial limitations of the available data. In this context, I am not convinced that the studies included in the review can fully answer the question. Modelling studies may provide more informative insights, particularly regarding the potential impact on INSTI resistance. The risk of resistance is what motivated the review, yet this crucial outcome is not considered.**

**Response:**

We thank the Academic Editor for this important comment. We agree that the current evidence base for HIV testing strategies in the context of LAI-PrEP remains limited, and we have revised the manuscript to reflect these constraints more clearly. Although our review includes data from the major global LAI-PrEP trials, such as HPTN 083, HPTN 084, PURPOSE 1, and PURPOSE 2, these studies were not designed to prospectively evaluate HIV testing algorithms, limiting the ability to draw firm conclusions about delayed diagnosis, diagnostic performance, or INSTI resistance. As a result, the available empirical data could be strengthened to more fully answer the question of the optimal HIV testing strategy for LAI-PrEP.

That said, the call for modelling data on the impact of resistance has been considered. We refer to Smith et al, which indicated RDTs were sufficient for resource-limited settings as part of the CAB-LA introduction, and NAT added high cost to the programme while providing a small benefit in reducing resistance, and did not improve survival or overall treatment success (<https://www.thelancet.com/journals/lanhiv/article/PIIS2352-3018(22)00365-4/fulltext>). Further, modelling evidence from van de Vijver 2025 (<https://www.abstract-archive.org/Abstract/Share/91175>) using a high incidence setting among men who have sex with men in Thailand also indicated similar findings that the concerns about resistance are on a more individual level and not a public health, population-level issue. We have referenced these as well to address the concern about the absence of modelling evidence.

**Despite the limited evidence, there is an urgent need to provide safe, feasible, and effective HIV testing strategies** as LAI-PrEP delivery expands rapidly. The availability of twice-yearly lenacapavir (LEN), in particular, has generated strong interest among programmes and policymakers, accelerating the demand for clear guidance on testing approaches that balance accuracy with scalability. Given the pace at which LAI-PrEP is being introduced, the absence of robust empirical data should not delay the development of provisional, pragmatic testing recommendations. Instead, programmes will need to rely on the best available evidence, while recognising its limitations, along with ongoing surveillance, until more definitive prospective data become available. Modelling data that the editor has pointed out as important also indicates that implementation of rapid tests, and even self-tests, may be sufficient without showing the need for NAT and further lab-based testing.

Regarding the risk of resistance, we discussed this in the Discussion. But we have added further discussion to strengthen the advantage of NAT in detecting early infection, which may have prevented INSTI RAMs.

Here is the original paragraph discussing about the risk of resistance in the Discussion. We have added information on the modelling related to resistance to Line 503.

*“[Line 512] Another concern regarding the use of RDTs compared to NAT-based testing strategies for LAI-PrEP is the potential for delayed diagnosis to contribute to the development of major RAMs,[1] particularly those with cross-resistance to current first-line INSTI therapies. However, it is important to note the limitations of these data. First, there is no direct evidence that NAT-based testing would prevent the development of resistance. Second, due to low viral loads associated with early infection, several of the cases with major INSTI RAMs, were detected using a single-genome sequencing INSTI drug resistance assay.[2, 3] This research methodology is usually not available for use in clinical diagnostic laboratories and has not been validated in the clinical diagnostic setting. Third, from indirect evidence, the use of HIVST compared with NAT or laboratory-based immunoassays had no significant impact on HIV deaths averted, despite a potential rise in resistance.[4] Further, there have been no well-described ART failures due to LAI-PrEP-associated INSTI resistance. Scaling-up LAI-PrEP requires not only simplified HIV testing but also an investment into monitoring for LAI-PrEP-associated resistance within established HIV surveillance programs. This should include monitoring for both LAI-PrEP-associated major RAMs and LAI-PrEP-associated treatment failures.”*

**Changes:**

[Line 69] NAT might have detected HIV before resistance emerged, though no prospective or modelling evidence showed clinical benefit at a population level.

[Line 395] A modelling study of CAB-LA in sub-Saharan Africa reported that NAT commodities are much more expensive ($22 United States Dollars (USD)) compared with 3^rd^ generation RDT ($4 USD), with a small benefit in reducing resistance but no improvement in survival or overall treatment success. [5].

[Line 461] In many settings, the use of an RDT as the initial screening test supports same-day PrEP initiation, consistent with WHO recommendations aimed at improving uptake and adherence among key populations. Conversely, whether confirmatory laboratory-based testing should be required prior to LAI-PrEP initiation warrants careful consideration. Confirmatory testing has clear diagnostic value, particularly for detecting early infection, but making it a mandatory prerequisite may inadvertently delay or restrict access in resource-constrained settings. This issue is evident in the US. Despite CDC recommendations, there were only 35% tested by HIV RNA within ± 14 days at CAB-LA initiation [55]. Although missed early diagnoses may have broader economic implications—including the cost of PrEP provision, delayed diagnosis, potential resistance, and onward transmission—the included studies did not provide sufficient prospective data to quantify these downstream effects. A modelling study shows that NAT adds significant cost to CAB-LA rollout programs in LMICs, but provides a small benefit in reducing resistance, and does not improve survival or overall treatment success [26]. Even high-income countries will benefit from RDT-based testing strategies, as evidence shows substantial feasibility constraints of using NAT for routine programmatic implementation [51]. This does not imply that NAT has no role in LAI-PrEP delivery. There may be settings and situations where it could be considered based on available resources.

[Line 499] Our findings showed that NAT could theoretically detect early or rare breakthrough infections during LAI-PrEP use. However, because NAT was used retrospectively on stored samples in HPTN 083 and 084—not prospectively as part of real-time clinical decision-making—there is no prospective evidence that earlier NAT detection would have prevented the development of INSTI resistance-associated mutations. In the real world, high-frequency follow-up testing, as done in studies, will likely be infeasible, and turnaround times in resource-limited settings may be lengthy. As a result, the impact of a slightly earlier diagnosis may be limited and ultimately not affect the small number of cases with resistance. Based on the data from real-world implementation, despite lower sensitivity of RDTs, there is little to no absolute difference in delayed HIV detection per 1,000 tests when using RDTs among CAB-LA or LEN users at initiation and for CAB-LA users at continuation. Further, using data from HPTN 083 open-label extension [7], detecting one additional case using NAT—missed by RDT at an earlier time point—would require testing 5,305 people using NAT. This suggests that a lot of resources are needed for minimal gain, using a public health lens.

[Line 527] Further, additional modelling suggests that the total number of resistance cases remains small even in very high incidence groups, such as MSM in Thailand.[66] While more sensitive tests could reduce resistance, the impact is minimal.[26, 66] For example, at 100% uptake of CAB-LA with RDTs, 6,200 new infections could be averted, and the difference in the number of INSTI-resistant and transmitted resistance cases remains very low (INSTI resistance: RDT: 94 vs NAT: 36, transmitted resistance: RDT: 15 vs NAT: 6). Thus, while more sensitive HIV testing will prevent some INSTI resistance, given the low INSTI resistance incidence, more sensitive testing is not critical for CAB-LA rollout.[26, 66] The benefits of a programme that is able to scale and get more people on PrEP, and at a fraction of the cost and more access-driven approaches, appear beneficial. Scaling-up LAI-PrEP requires not only simplified HIV testing but also an investment in monitoring for LAI-PrEP-associated resistance within established HIV surveillance programs. This should include monitoring for both LAI-PrEP-associated major RAMs and LAI-PrEP-associated treatment failures.

[Line 587] Due to these limitations, our findings and conclusions about the comparative performance of different testing strategies for LAI-PrEP should be interpreted cautiously.

**2. Regarding test accuracy, the authors limited their search to the LAI-PrEP context, but there is a substantial existing literature, including numerous studies and systematic reviews/meta-analyses, evaluating the accuracy of various RDTs compared with NAT or laboratory-based immunoassays. This evidence should be discussed in greater depth. As reviewer 2 notes, accuracy varies considerably across different RDTs.**

**Response:**

We thank the editor for this comment and acknowledge that a clear explanation of the expected and observed performance of various HIV detection methods is critical to the overall interpretation of this data. A description of the performance of HIV diagnostic tests has now been added to the discussion.

We also revised the Introduction and Discussion to highlight that this review extends beyond comparing the analytical accuracy of HIV tests and evaluates how different testing approaches affect clinical outcomes and programme delivery for LAI-PrEP. Diagnostic accuracy is important, but the clinical and public health value of LAI-PrEP depends equally on whether testing strategies are feasible, scalable, and accessible.

**Changes:**

[Line 38] **Background:** There is strong interest among communities and policymakers for long-acting injectable pre-exposure prophylaxis (LAI-PrEP) scale-up, accelerating the demand for clear guidance on testing approaches that balance accuracy with scalability. Unlike oral pre-exposure prophylaxis, LAI-PrEP results in prolonged subtherapeutic drug levels after discontinuation, which can increase the risk of drug resistance among those who have an undetected HIV infection. This systematic review extends beyond comparing the analytical accuracy of HIV tests, and evaluates how different HIV testing strategies, including rapid diagnostic tests (RDTs), laboratory-based immunoassays and nucleic acid testing (NAT), affect clinical utility and programme delivery.

[Line 158] There is a strong interest among communities and policymakers for LAI PrEP scale-up, accelerating the demand for clear guidance on testing approaches that balance accuracy with scalability [9]. Given the pace at which LAI-PrEP is being introduced, robust data is needed for the development of provisional, pragmatic testing recommendations. This review extends beyond comparing the analytical accuracy of HIV tests and evaluates how different testing approaches, including RDTs, HIVST, laboratory-based immunoassays and NAT techniques, affect clinical outcomes and programme delivery for LAI-PrEP. Diagnostic accuracy is important, but the clinical and public health value of LAI-PrEP depends equally on whether testing strategies are feasible, scalable, and accessible. Our systematic review examined clinical and diagnostic outcomes across LAI-PrEP initiation, re-initiation and continuation to inform the WHO guidelines.

[Line 422] **Discussion**

This systematic review synthesises evidence on different LAI-PrEP testing strategies, focusing on RDTs versus NAT or laboratory-based immunoassays. A key contribution of this work is its focus on the clinical utility and program delivery implications of testing strategies, rather than diagnostic accuracy in isolation.

[Line 486] RDTs have reduced sensitivity in detecting HIV in its early stages [55], but our analysis suggests this has minimal impact on absolute clinical outcomes at the population level. Evidence from HIV-1 seroconversion panels shows the median detection window of 5.9 days for NAT and antigen/antibody (Ag/Ab) laboratory immunoassays, 7.4 days for 4^th^-generation (Ag/Ab) RDTs and 14.9 days for 3^rd^-generation (IgM-sensitive) RDTs. Given the expected window periods of various HIV detection methods and known attenuation of serological responses in the setting of PrEP, it may be pertinent for programmes to consider repeat testing at 2-4 weeks after LAI-PrEP initiation if 3^rd^ generation RDTs are being used and for individuals with recent high-risk exposures. Historically, delayed diagnosis has been a concern due to the high viral loads observed in early infection in PrEP-naive individuals [60, 61]. Given the lower viral loads observed in early LAI-PrEP infections, the transmission risk associated with these infections is likely to be lower than for PrEP-naive individuals [62]. Our findings showed that NAT could theoretically detect early or rare breakthrough infections during LAI-PrEP use. However, because NAT was used retrospectively on stored samples in HPTN 083 and 084—not prospectively as part of real-time clinical decision-making—there is no prospective evidence that earlier NAT detection would have prevented the development of INSTI resistance-associated mutations. In the real world, high-frequency follow-up testing, as done in studies, will likely be infeasible, and turnaround times in resource-limited settings may be lengthy. As a result, the benefit of a slightly earlier diagnosis may be limited and ultimately not affect the small number of cases with resistance [26]. Based on the data from real-world implementation, despite lower sensitivity of RDTs, there is little to no absolute difference in delayed HIV detection per 1,000 tests when using RDTs among CAB-LA or LEN users at initiation and for CAB-LA users at continuation. Further, using data from HPTN 083 open-label extension [48], detecting one additional case using NAT—missed by RDT at an earlier time point—would require testing 5,305 people using NAT. This suggests that a lot of resources are needed for minimal gain, using a public health lens.

**Reviewer #1: This is a well-written manuscript on an important topic -- what type of HIV testing might best be used in assessing HIV infection status in persons on long-acting injectable pre-exposure prophylaxis (PrEP). The authors have undertaken a systematic review of rapid, laboratory-based immunoassay and nucleic acid testing strategies. For the most part, the paper is balanced, the authors acknowledge the severe limitations of the current data available. However, in some spots, it seems they under-emphasize some of the advantages of laboratory and/or NAT testing. They also speak to the advantages of self-testing, which is not at all addressed by the current set of studies. While I agree with their premise that overall the benefits of RDT outweigh the downsides, the authors could be more balanced in their treatment of the data.**

**Response:** We thank the Reviewer for your acknowledgement of our work and insightful suggestions. We have attempted to address your comments by providing detailed, point-by-point responses below.

**Major comments:**

**1. The authors state the benefits of the speed of results with RDTs compared to NAT and lab-based assays. However, this is generally a matter of several days difference, whereas the more sensitive NAT testing may pick up infections months earlier. In terms of speed of getting information on HIV status for the rare breakthrough infections, it seems that NAT would be the winner.**

**Response:**

We thank the Reviewer for this important comment. We agree that NAT has higher analytical sensitivity and can theoretically detect HIV earlier in the infection course, including potential early or rare breakthrough infections. However, our review extends beyond comparing the analytical accuracy of HIV tests and evaluates how different testing approaches affect the clinical utility and programme delivery for LAI-PrEP. Diagnostic accuracy is important, but the clinical and public health value of LAI-PrEP depends equally on whether testing strategies are feasible, scalable, and accessible.

Clinical decision-making, such as LAI-PrEP initiation, linkage to care, and avoiding unnecessary PrEP holds, depends on the turnaround time of returned results rather than theoretical diagnostic sensitivity. In practice, NAT results often require several days to a week to return, whereas RDTs enable same-day clinical action. The WHO Guidelines Development Group also considered evidence from a systematic review of early infant diagnosis, which found that turnaround times from sample collection to result delivery to the caregiver could take a median of 35 days[5] as well as another review indicating a median of around 60 days.[14] Further, it would require testing 5,305 people using NAT to detect one missed case by RDTs. This suggests that a lot of resources are needed for minimal gain, from a public health lens.

Additionally, although our findings showed that NAT could theoretically detect early or rare breakthrough infections during LAI-PrEP use, it was used retrospectively on stored samples in HPTN 083 and 084—not prospectively as part of real-time clinical decision-making. Thus, there is no prospective evidence that earlier NAT detection would have prevented the development of INSTI resistance-associated mutations. In the real world, high-frequency follow-up testing, as done in studies, will likely be infeasible, and turnaround times in resource-limited settings may be lengthy. A modelling study of CAB-LA roll-out showed that NAT added high cost to the programme while providing a small benefit in reducing resistance, and did not improve survival or overall treatment success [26].

We agree with the Reviewer that we need to acknowledge NAT’s diagnostic advantages and have added text to clarify this point in the Discussion.

**Changes:**

[Line 399] A modelling study of CAB-LA in sub-Saharan Africa reported that NAT commodities are much more expensive ($22 United States Dollars (USD)) compared with 3^rd^ generation RDT ($4 USD), with a small benefit in reducing resistance but no improvement in survival or overall treatment success. [26]. Other studies confirm that the cost of NAT is significantly more than that of RDT or laboratory-based immunoassays (Appendix 12) [21-25]. We calculated, based on the reported data, that to detect one additional HIV case, missed by a rapid test, using NAT requires testing 5,305 people with estimated costs of $46,684 - $451,456 [48].

[Line 442] While laboratory-based test results are usually returned within a week, programme-level turnaround times can vary substantially across settings. For example, in early infant diagnosis programmes, the median time from sample collection to result delivery to the caregiver has been reported as long as 35 days and 60 days, compared with same-day results for RDTs [26, 52]. This difference could be from operational and communication delays rather than laboratory processing time, and it highlights that RDTs are more likely to consistently provide substantially faster results, linkage to care and ART initiation across diverse service settings—a pattern likely to be similar when implementing RDTs within LAI-PrEP delivery

[Line 465] Confirmatory testing has clear diagnostic value, particularly for detecting early infection, but making it a mandatory prerequisite may inadvertently delay or restrict access in resource-constrained settings. This issue is evident in the US. Despite CDC recommendations, there were only 35% tested by HIV RNA within ± 14 days at CAB-LA initiation [55]. Although missed early diagnoses may have broader economic implications—including the cost of PrEP provision, delayed diagnosis, potential resistance, and onward transmission—the included studies did not provide sufficient prospective data to quantify these downstream effects. A modelling study shows that NAT adds significant cost to CAB-LA rollout programs in LMICs, but provides a small benefit in reducing resistance, and does not improve survival or overall treatment success [26]. Even high-income countries will benefit from RDT-based testing strategies, as evidence shows substantial feasibility constraints of using NAT for routine programmatic implementation [51]. This does not imply that NAT has no role in LAI-PrEP delivery. There may be settings and situations where it could be considered based on available resources.

[Line 499] Our findings showed that NAT could theoretically detect early or rare breakthrough infections during LAI-PrEP use. However, because NAT was used retrospectively on stored samples in HPTN 083 and 084—not prospectively as part of real-time clinical decision-making—there is no prospective evidence that earlier NAT detection would have prevented the development of INSTI resistance-associated mutations. In the real world, high-frequency follow-up testing, as done in studies, will likely be infeasible, and turnaround times in resource-limited settings may be lengthy. As a result, the benefit of a slightly earlier diagnosis may be limited and ultimately not affect the small number of cases with resistance [26]. Based on the data from real-world implementation, despite lower sensitivity of RDTs, there is little to no absolute difference in delayed HIV detection per 1,000 tests when using RDTs among CAB-LA or LEN users at initiation and for CAB-LA users at continuation. Further, using data from HPTN 083 open-label extension [48], detecting one additional case using NAT—missed by RDT at an earlier time point—would require testing 5,305 people using NAT. This suggests that a lot of resources are needed for minimal gain, using a public health lens.

[Line 527] Further, additional modelling suggests that the total number of resistance cases remains small even in very high incidence groups, such as MSM in Thailand.[66] While more sensitive tests could reduce resistance, the impact is minimal.[26, 66] For example, at 100% uptake of CAB-LA with RDTs, 6,200 new infections could be averted, and the difference in the number of INSTI-resistant and transmitted resistance cases remains very low (INSTI resistance: RDT: 94 vs NAT: 36, transmitted resistance: RDT: 15 vs NAT: 6). Thus, while more sensitive HIV testing will prevent some INSTI resistance, given the low INSTI resistance incidence, more sensitive testing is not critical for CAB-LA rollout.[26, 66] The benefits of a programme that is able to scale and get more people on PrEP, and at a fraction of the cost and more access-driven approaches, appear beneficial. Scaling-up LAI-PrEP requires not only simplified HIV testing but also an investment in monitoring for LAI-PrEP-associated resistance within established HIV surveillance programs. This should include monitoring for both LAI-PrEP-associated major RAMs and LAI-PrEP-associated treatment failures.

**2. The authors cite the proportion of delayed infections using the denominator of all persons in the study (line 278) rather than just of those found to be HIV positive. Should the denominator be the latter, rather than the former?**

**Response:**

We used the entire cohort as the denominator because our primary objective was to estimate the absolute risk of delayed HIV detection at the population/programmatic level, which aligns with public health decision-making for LAI-PrEP scale-up. This approach allows comparison across testing strategies in terms of absolute numbers of delayed diagnoses per 1,000 people tested, which is the relevant metric for implementation planning. We have added a clarification in the methods section.

**Changes:**

[Line 220] We reported the absolute risk of delayed HIV detection at the population/programmatic level to compare across testing strategies in terms of absolute numbers of delayed diagnoses per 1,000 people tested, which is the relevant metric for implementation planning.

**3. In citing differences in resistance-associated mutations, the authors state, "...there is no direct evidence that the testing strategy would affect the development of resistance-associated mutations" (lines 311-313). However, the data do suggest that 80% of the infections in HPTN 083 suggested that NAT would have picked up the resistance prior to emergence of INSTI RAMs.**

**Response:**

We thank the reviewer for this insightful comment. We agree that data from HPTN 083 indicate that NAT retrospectively detected HIV infection earlier in 8 of the 10 CAB-LA breakthrough cases with major INSTI RAMs. The distinction we intended to highlight is that there is currently no prospective evidence demonstrating that using NAT as the routine clinical testing strategy prevents the development of resistance-associated mutations. In both HPTN 083 and 084, NAT was conducted retrospectively on stored samples after a positive RDT/immunoassay result. NAT was not used in real time for clinical decision-making. In the real world, high-frequency follow-up testing, as done in studies, will likely be infeasible, and turnaround times in resource-limited settings may be lengthy. We also noted that due to low viral loads associated with early infection, several of the cases with major INSTI RAMs, were detected using a single-genome sequencing INSTI drug resistance assay.[2, 3] This research methodology is usually not available for use in clinical diagnostic laboratories and has not been validated in the clinical diagnostic setting.

We also noted that 22 false positives from NAT could lead to PrEP holds and challenges, in addition to high cost. Further, modelling evidence from van de Vijver 2025 using a high incidence setting among men who have sex with men in Thailand also indicated similar findings that the concerns about resistance are on a more individual level and not a public health, population-level issue.[8] A modelling study of CAB-LA roll-out showed that NAT added high cost to the programme while providing a small benefit in reducing resistance, and did not improve survival or overall treatment success [26].

Therefore, although NAT could have detected infection earlier in retrospective analyses, these findings do not establish that a NAT-based strategy would have altered clinical outcomes or prevented RAM emergence in practice. We have revised the Results and Discussion to make this distinction clearer.

**Changes:**

[Line 386] Of the 10 major INSTI RAMs detected in CAB users described in HPTN 083, retrospective testing of stored samples after a RDT positive result suggests that NAT could have detected the HIV infection before INSTI RAMs emerged in eight cases [15-27]. However, this has not been demonstrated in prospective studies.

[Line 399] A modelling study of CAB-LA in sub-Saharan Africa reported that NAT commodities are much more expensive ($22 United States Dollars (USD)) compared with 3^rd^ generation RDT ($4 USD), with a small benefit in reducing resistance but no improvement in survival or overall treatment success. [26].

[Line 472] A modelling study shows that NAT adds significant cost to CAB-LA rollout programs in LMICs, but provides a small benefit in reducing resistance, and does not improve survival or overall treatment success [26].

[Line 499] Our findings showed that NAT could theoretically detect early or rare breakthrough infections during LAI-PrEP use. However, because NAT was used retrospectively on stored samples in HPTN 083 and 084—not prospectively as part of real-time clinical decision-making—there is no prospective evidence that earlier NAT detection would have prevented the development of INSTI resistance-associated mutations. In the real world, high-frequency follow-up testing, as done in studies, will likely be infeasible, and turnaround times in resource-limited settings may be lengthy. As a result, the benefit of a slightly earlier diagnosis may be limited and ultimately not affect the small number of cases with resistance [26]. Based on the data from real-world implementation, despite lower sensitivity of RDTs, there is little to no absolute difference in delayed HIV detection per 1,000 tests when using RDTs among CAB-LA or LEN users at initiation and for CAB-LA users at continuation. Further, using data from HPTN 083 open-label extension [48], detecting one additional case using NAT—missed by RDT at an earlier time point—would require testing 5,305 people using NAT. This suggests that a lot of resources are needed for minimal gain, using a public health lens.

[Line 527] Further, additional modelling suggests that the total number of resistance cases remains small even in very high incidence groups, such as MSM in Thailand.[66] While more sensitive tests could reduce resistance, the impact is minimal.[26, 66] For example, at 100% uptake of CAB-LA with RDTs, 6,200 new infections could be averted, and the difference in the number of INSTI-resistant and transmitted resistance cases remains very low (INSTI resistance: RDT: 94 vs NAT: 36, transmitted resistance: RDT: 15 vs NAT: 6). Thus, while more sensitive HIV testing will prevent some INSTI resistance, given the low INSTI resistance incidence, more sensitive testing is not critical for CAB-LA rollout.[26, 66] The benefits of a programme that is able to scale and get more people on PrEP, and at a fraction of the cost and more access-driven approaches, appear beneficial. Scaling-up LAI-PrEP requires not only simplified HIV testing but also an investment in monitoring for LAI-PrEP-associated resistance within established HIV surveillance programs. This should include monitoring for both LAI-PrEP-associated major RAMs and LAI-PrEP-associated treatment failures.

**4. The authors talk about the benefits of oral self-testing, but none of the data evaluated this method of diagnosis, and the window period for oral testing is considerably longer (~12 weeks) than even RDT and certainly than lab-based and NAT testing. The reference to oral testing seems to be misplaced in this description of the literature.**

**Response:**

We thank the reviewer for this important clarification. We agree that none of the studies included in our review evaluated oral-fluid HIVST for LAI-PrEP delivery, and that oral-fluid tests have a considerably longer window period than blood-based RDTs and laboratory assays. Our review may have given the impression that oral-fluid HIVST was part of the evidence base reviewed.

Our intention was to describe HIVST in general terms, as several programmes have considered HIVST as part of differentiated testing strategies for LAI-PrEP. However, we agree that mentioning HIVST in the Results may be misleading, and decided to remove it from the Results and revised the Discussion.

**Changes:**

[Line 407] **Acceptability and impact on equity**

RDTs (compared to laboratory-based testing) could improve equitable access to LAI-PrEP by expanding service delivery options, especially for underserved populations. Limited NAT testing capacity in low-middle-income countries (LMICs) presents a barrier to LAI-PrEP implementation, with high costs and logistical demands limiting scalability.

[Line 543] We identified limited evidence on HIVST for LAI-PrEP delivery. Outcomes data on HIVST were only available for testing frequency. Novel uses of HIVST for LAI-PrEP delivery are emerging, such as in Brazil, where clients self-test before their first LAI-PrEP [1, 10]. Emerging evidence suggests HIVST, performing similarly to HIV RDTs, might be sufficiently accurate for LAI-PrEP implementation [29]. While this shows promise, and HIVST can be an option, we noted that oral-fluid HIVST or RDTs have a considerably longer window period than blood-based RDTs and laboratory assays [67, 68]. Further implementation research is needed to fully understand the role of HIVST in guiding LAI-PrEP delivery.

**5. Is the turnaround time for NAT testing really 35 days (line 367)? That seems quite long compared to what is available in clinical practice.**

**Response:**

The 35-day turnaround time cited in the Discussion was drawn from a systematic review evaluating point-of-care in infant HIV diagnosis, where the outcome measured was the total time from sample collection to result delivery to the caregiver. This figure reflects broader programme-level delays, including specimen transport across different settings and communication, not the processing time of laboratory-based tests themselves.

For LAI-PrEP, our review found that laboratory-based immunoassay turnaround times were 1-5 days and up to 7 days for NAT, which aligns with clinical practice. To avoid misinterpretation, we have revised the text to clarify the context of the 35-day figure.

**Changes:**

[Line 442] While laboratory-based test results are usually returned within a week, programme-level turnaround times can vary substantially across settings. For example, in early infant diagnosis programmes, the median time from sample collection to result delivery to the caregiver has been reported as long as 35 days and 60 days, compared with same-day results for RDTs [26, 52]. This difference could be from operational and communication delays rather than laboratory processing time, and it highlights that RDTs are more likely to consistently provide substantially faster results, linkage to care and ART initiation across diverse service settings—a pattern likely to be similar when implementing RDTs within LAI-PrEP delivery.

**6. The authors also state that RDT allows for same-day starts, a very important component. However, most guidelines do allow for screening with RDTs and then confirmation with lab-based testing, which wouldn't actually rule out same-day starts. The authors might comment on whether or not confirmatory testing is needed, rather than stating that testing other than RDTs would necessarily rule out same-day starts.**

**Response:**

We thank the reviewer for this thoughtful comment. Our intention was not to imply that confirmatory testing excludes same-day initiation, but rather to highlight the operational implications of relying solely on laboratory-based tests as the initial screening modality. To address this, we have revised the text to provide a more nuanced discussion.

**Changes:**

[Line 456] Simplified and affordable testing approaches that rely on RDTs and HIVST are important to ensuring LAI-PrEP has a public health impact. Our findings highlight the benefits of transitioning global programming to RDTs, particularly for marginalised and mobile populations or those in LMICs because of their ease of use, reduced infrastructure requirements and widespread availability [54]. In many settings, the use of an RDT as the initial screening test supports same-day PrEP initiation, consistent with WHO recommendations aimed at improving uptake and adherence among key populations. Conversely, whether confirmatory laboratory-based testing should be required prior to LAI-PrEP initiation warrants careful consideration. Confirmatory testing has clear diagnostic value, particularly for detecting early infection, but making it a mandatory prerequisite may inadvertently delay or restrict access in resource-constrained settings. This issue is evident in the US. Despite CDC recommendations, there were only 35% tested by HIV RNA within ± 14 days at CAB-LA initiation [55]. Although missed early diagnoses may have broader economic implications—including the cost of PrEP provision, delayed diagnosis, potential resistance, and onward transmission—the included studies did not provide sufficient prospective data to quantify these downstream effects. A modelling study shows that NAT adds significant cost to CAB-LA rollout programs in LMICs, but provides a small benefit in reducing resistance, and does not improve survival or overall treatment success [26]. Even high-income countries will benefit from RDT-based testing strategies, as evidence shows substantial feasibility constraints of using NAT for routine programmatic implementation [51]. This does not imply that NAT has no role in LAI-PrEP delivery. There may be settings and situations where it could be considered based on available resources. While individuals and programmes may use various PrEP options, such as oral PrEP and DVRs, using RDT-based testing strategies provides a practical and scalable alternative that supports differentiated service delivery for PrEP, ensuring timely access to PrEP continuation and linkage to confirmatory testing, particularly in resource-limited settings. Considering the growing financial constraints facing global health, and especially HIV testing and prevention programmes, simple and flexible tools are essential for future LAI-PrEP delivery.

**Minor comments:**

**1. Line 108: the authors refer to IAS guidelines -- did they intend this to be IAS-USA guidelines?**

**Changes:**

[Line 154] the International Antiviral Society United States of America (IAS-USA)

**Reviewer #2:** Thank you for your recent submission assessing long acting injectable PrEP for HIV patients. From a methodological standpoint the review is conducted using gold standard methods. A highlight being the extensive literature searching.

**Response:**

We thank the Reviewer for your acknowledgement of our work.

**1. The analysis of odds ratio data was conducted using the PM fixed effect model, which seems appropriate due to small/no events and lack of heterogeneity. However, your protocol states you planned to conduct a bivariate analysis of the sensitivity and specificity for diagnostic accuracy. This does not appear to have been conducted and no reasoning appears to be presented. It seems potentially feasible for LA-CAB with five studies being available. If the authors deemed it unfeasible, could they please add detail to state why it was not.**

**Response:**

Thank you for this thoughtful comment. Our protocol did state an intention to perform a bivariate meta-analysis of sensitivity and specificity for diagnostic accuracy. After completing data extraction, we determined that a bivariate analysis was not feasible because of insufficient data. Although five LA-CAB studies reported some diagnostic accuracy outcomes, the underlying data required for a bivariate model (i.e., paired 2×2 tables with true positives, false positives, false negatives, and true negatives from the same test at the same timepoint) were rarely available. The studies only reported partial diagnostic outcomes or reported them at different and non-comparable timepoints (e.g., retrospective NAT testing on stored samples, RDT used only at enrolment, immunoassay used only after a reactive screen). Several LA-CAB breakthrough cases were identified retrospectively with NAT applied only after an RDT-positive result, which precluded construction of a proper paired dataset required for bivariate modelling. So, we opted instead to report diagnostic outcomes descriptively and to present available predictive values (PPV, NPV) and detection timings narratively. We have now added text to the Methods sections clarifying why the planned bivariate analysis could not be conducted and noting this as a deviation from the protocol.

**Changes:**

[Line 216] Although the protocol prespecified a bivariate analysis of diagnostic accuracy, the necessary paired 2×2 data were not available across studies. Due to inconsistent testing algorithms and retrospective use of NAT in several trials, a formal bivariate meta-analysis could not be performed.

**2. Could the authors provide further information on how GRADE was conducted. Especially, where evidence was meta-analysed. Did the authors use the guidelines by Murad and colleagues (2017:** [**https://urldefense.com/v3/__https://doi.org/10.1136/ebmed-2017-110668)?__;!!NDYExDT0u85SdT4!suyz4DXKqbF9R_592p1f0Y6M5iptFx7Bo-qRozIR19qxM95wTvcVzqUljc7FlDPTPrC8qBkz17gYeA$**](https://aus01.safelinks.protection.outlook.com/?url=https%3A%2F%2Furldefense.com%2Fv3%2F__https%3A%2F%2Fdoi.org%2F10.1136%2Febmed-2017-110668)%3F__%3B!!NDYExDT0u85SdT4!suyz4DXKqbF9R_592p1f0Y6M5iptFx7Bo-qRozIR19qxM95wTvcVzqUljc7FlDPTPrC8qBkz17gYeA%24&data=05%7C02%7C%7C25887d699e154cee72ed08de296c2e9d%7Ce21c8db98aa843019657077a0415bdf1%7C0%7C0%7C638993741776843186%7CUnknown%7CTWFpbGZsb3d8eyJFbXB0eU1hcGkiOnRydWUsIlYiOiIwLjAuMDAwMCIsIlAiOiJXaW4zMiIsIkFOIjoiTWFpbCIsIldUIjoyfQ%3D%3D%7C0%7C%7C%7C&sdata=iolMFY3hFghSCvrdmEZfTurxlfaf02kfVwy5qbfKbC8%3D&reserved=0) **If not, the authors may wish to consider this methodology where they do not have a single point estimate. Additionally, if they did use this method this should be stated clearly with the appropriate reference.**

**Response:**

We were guided by an expert GRADE methodologist (Nandi Siegfried) throughout this process and followed the World Health Organisation Handbook for Guideline Development (<https://www.who.int/publications/i/item/9789241548960>) and GRADE Handbook (<https://gdt.gradepro.org/app/handbook/handbook.html>)

**Changes:**

[Line 173] This systematic review was conducted following the WHO Handbook for Guideline Development [34] and the Cochrane Handbook for Systematic Reviews of Interventions [35] and reported findings according to the PRISMA Reporting Guideline (S1 Checklist) [36].

[Line 233] GRADE methodology was used to assess the certainty of evidence (S4 Appendix) [15, 20, 21].

**3. I think the discussion, at times, is a little over positive with the findings of the review. It should emphasis that there is a lack of evidence and the quality of the evidence is low/very low. This is mentioned for some outcomes but I feel this may stretch across all outcomes given the current GRADE assessments, risk of bias, and sparsity of data.**

**Response:**

We thank the Reviewer for their suggestion to more prominently reflect the limitations of the evidence base throughout the Results and Discussion. In response, we have systematically incorporated the corresponding GRADE ratings (“low certainty” or “very low certainty”) across all outcomes in the Results section. We have also revised the Discussion to more explicitly emphasise the limited availability of evidence, the low or very low certainty across nearly all outcomes assessed, and the need for well-designed prospective studies to strengthen the evidence base for LAI-PrEP testing strategies.

**Changes:**

[Throughout the Results section] We added explicit statements of “low certainty” or “very low certainty” alongside each reported outcome to ensure consistent alignment with the GRADE assessments.

[Line 554] Several gaps require further investigation to optimise HIV testing strategies for LAI-PrEP. To date, no prospective studies have directly compared the clinical or diagnostic outcomes of different testing strategies for LAI-PrEP. We acknowledge the limited number of studies, heterogeneity in study methods, and risk-of-bias concerns. Consequently, the overall certainty of evidence remains very low or low and could be strengthened through further modelling analyses and prospective studies that provide insight into broader economic implications (e.g., the cost of PrEP provision, delayed diagnosis, potential resistance, and onward transmission), optimal testing strategies and approaches for LAI-PrEP, including for new and emerging PrEP agents.

[Line 571] Our study's main strength was that we systematically reviewed all published papers related to HIV testing in LAI-PrEP, collaborating with the WHO for information through an open call, and directly contacting authors for details. However, our assessment of performance accuracy in different testing strategies may introduce selection bias. For example, studies (e.g., HPTN 083 and 084) included in this review used NAT for pre-screening and RDT/laboratory-based immunoassay at enrolment, potentially favouring NAT’s sensitivity estimates, as we could only compare new seroconversions at the initiation and continuation of CAB-LA. The gap between pre-screening and enrolment also created the potential for undetected seroconversion. Individuals with a positive RDT/laboratory-based immunoassay/NAT were excluded from enrolment, whereas NAT was retrospectively performed on stored samples post-enrolment. This screening strategy likely explains the zero infections detected by RDT/laboratory-based immunoassay at CAB-LA initiation, compared to 13 by NAT. Importantly, there is no direct evidence confirming that pre-enrolment NAT results were not false negatives, nor can the absolute number of false negatives from NAT be determined, as NAT was only performed when RDT was positive. Additionally, there were limited data on the clinical and diagnostic outcomes for HIVST, social harms and sexual behaviours of those receiving misdiagnosis or developing resistance-associated mutations from misdiagnosis. Due to these limitations, our findings and conclusions about the comparative performance of different testing strategies for LAI-PrEP should be interpreted cautiously.

**Reviewer #3: This systematic review aims to assess the effects of different testing strategies to support long-acting injectable pre-exposure prophylaxis roll-out. The strength of the paper is the considerable manuscripts and abstracts that were reviewed. However, I major limitation is the conclusion that rapid diagnostic tests should be used despite low certainty of evidence, the evidence clearly favoring nucleic acid tests, a discussion of acute HIV infection and the detrimental effects of starting someone on LAI-PrEP when they really need treatment and the further potential transmission risks.**

**Response:**

We thank the Reviewer for your acknowledgement of our work and insightful suggestions. We have attempted to address your comments by providing detailed, point-by-point responses below.

**My main suggestions are that as follows:**

**1) Replace table 1 which discusses the main characteristics of the study with a table that lists every paper that was included in the review (n=22) with details about a) authors b) publication date c) country of study d) test used e) study design f) type of PrEP g) study population h) sample size i) outcome of interest k) main result. Include a similar section for abstracts that were included.**

**Response:**

We thank the reviewer for this helpful suggestion. The detailed information requested is provided in S5 Appendix, where we present the full list of included studies with corresponding characteristics.

In this review, we grouped evidence by study/program rather than by publication because several studies generated multiple publications, often reporting overlapping cohorts, different study periods, or related outcomes. We also incorporated additional information obtained directly from study authors and the WHO team to ensure completeness. Presenting these as individual papers in the main table would therefore create duplication and potentially confuse the interpretation of study-level implementation findings.

We would prefer to retain Table 1 in its current form because it provides a concise, high-level overview of the included studies, which we believe helps readers understand the landscape and diversity of HIV testing approaches within LAI-PrEP delivery. We have added a footnote in Table 1’s legend that detailed study-level characteristics are available in S5 Appendix, as recommended. However, we are happy to revise Table 1 further if the editors feel that a publication-based rather than study-based structure would enhance clarity.

**Changes:**

[Table 1- added a footnote] Detailed study-level characteristics can be found in S5 Appendix.

**2) In table 2, it would beneficial to the reader to see the studies from which these findings came and how each of those studies were classified using GRADE**

**Response:**

We thank the reviewer for this helpful suggestion. We agree that it is important for readers to understand where each outcome originated and how the certainty of evidence was assessed. Because our review synthesised findings at the study/program level, drawing on data from published papers, author-provided information, and submissions through the WHO open call, linking outcomes to individual publications within Table 2 would not accurately reflect how the evidence was compiled. Several studies contributed multiple data sources across different time periods, and listing individual references in the main table could inadvertently be misleading or confusing.

To improve clarity while preserving the integrity of the study-level synthesis, we have updated Table 2 to include a reference to the specific appendix where each outcome is fully described, including the contributing studies and supporting data. The detailed GRADE table is available in S4 Appendix.

We hope that these additions make Table 2 easier to navigate while maintaining transparency about how the data were collated.

**Changes:**

‘Appendix no.’ is added to each outcome in Table 2.

**3) Some discussion of the different generations of enzyme immunoassays for HIV and the window of detection is warranted as well as the importance of acute HIV infection and challenges of diagnosis with earlier generation tests. A fourth-generation assay would detect acute HIV but the earlier (1st, 2nd, 3rd) will likely miss infections. This is an important consideration as resistance can be bred if an infection person if put on monotherapy. Here a discussion of repeat testing if using older generation and a risk assessment to understand recent exposures is important.**

**Response:**

We thank the Reviewer for this suggestion and have now incorporated a discussion of the different generations of immunoassays for HIV and window periods for detection of primary HIV infection. We agree that this is an important consideration for HIV PrEP usage due to the risk of primary infection during initiation of a single antiretroviral agent, which will effectively comprise monotherapy.

**Changes:**

[Line 488] Evidence from HIV-1 seroconversion panels shows the median detection window of 5.9 days for NAT and antigen/antibody (Ag/Ab) laboratory immunoassays, 7.4 days for 4^th^-generation (Ag/Ab) RDTs and 14.9 days for 3^rd^-generation (IgM-sensitive) RDTs. Given the expected window periods of various HIV detection methods and known attenuation of serological responses in the setting of PrEP, it may be pertinent for programmes to consider repeat testing at 2-4 weeks after LAI-PrEP initiation if 3^rd^-generation RDTs are being used and for individuals with recent high-risk exposures. Historically, delayed diagnosis has been a concern due to the high viral loads observed in early infection in PrEP-naive individuals [11, 12]. Given the lower viral loads observed in early LAI-PrEP infections, the transmission risk associated with these infections is likely to be lower than for PrEP-naive individuals [13].

[Line 527] Further, additional modelling suggests that the total number of resistance cases remains small even in very high incidence groups, such as MSM in Thailand.[66] While more sensitive tests could reduce resistance, the impact is minimal.[26, 66] For example, at 100% uptake of CAB-LA with RDTs, 6,200 new infections could be averted, and the difference in the number of INSTI-resistant and transmitted resistance cases remains very low (INSTI resistance: RDT: 94 vs NAT: 36, transmitted resistance: RDT: 15 vs NAT: 6). Thus, while more sensitive HIV testing will prevent some INSTI resistance, given the low INSTI resistance incidence, more sensitive testing is not critical for CAB-LA rollout.[26, 66] The benefits of a programme that is able to scale and get more people on PrEP, and at a fraction of the cost and more access-driven approaches, appear beneficial. Scaling-up LAI-PrEP requires not only simplified HIV testing but also an investment in monitoring for LAI-PrEP-associated resistance within established HIV surveillance programs. This should include monitoring for both LAI-PrEP-associated major RAMs and LAI-PrEP-associated treatment failures.

**4) Please describe in more detail the approach to the systematic review using PRISMA and include supplemental tables as well as the checklist. For example, please include a table of inclusion and exclusion criteria. See Liberati A, Altman DG, Tetzlaff J, et al. The PRISMA statement for reporting systematic reviews and meta-analyses of studies that evaluate health care interventions: explanation and elaboration. PLoS Med2009;6:e1000100. doi:10.1371/journal.pmed.1000100 pmid:19621070**

**Response:**

We have reviewed the relevant documents, submitted the PRISMA Checklist and PRISMA Abstract Checklist in the S1 Checklist file, and provided all additional documents, such as inclusion and exclusion criteria, risk of bias assessment, and GRADE assessment tables, in the Appendix. PRISMA Flowchart can be found in Figure 1.

**5) The forest plot clearly shows that the data favor NAT. In a high-risk population, it is even more important to ensure a client's HIV negative status before starting PrEP. For the general population and HIV screening programs, the diagnostic accuracy might be compatible but is that true for high risk populations? Aren't their special considerations for testing persons at high risk of HIV acquisition? See:** [**https://urldefense.com/v3/__https://www.sciencedirect.com/science/article/pii/S1386653212000467?via*3Dihub__;JQ!!NDYExDT0u85SdT4!suyz4DXKqbF9R_592p1f0Y6M5iptFx7Bo-qRozIR19qxM95wTvcVzqUljc7FlDPTPrC8qBnZVXIK5g$**](https://aus01.safelinks.protection.outlook.com/?url=https%3A%2F%2Furldefense.com%2Fv3%2F__https%3A%2F%2Fwww.sciencedirect.com%2Fscience%2Farticle%2Fpii%2FS1386653212000467%3Fvia*3Dihub__%3BJQ!!NDYExDT0u85SdT4!suyz4DXKqbF9R_592p1f0Y6M5iptFx7Bo-qRozIR19qxM95wTvcVzqUljc7FlDPTPrC8qBnZVXIK5g%24&data=05%7C02%7C%7C25887d699e154cee72ed08de296c2e9d%7Ce21c8db98aa843019657077a0415bdf1%7C0%7C0%7C638993741776858770%7CUnknown%7CTWFpbGZsb3d8eyJFbXB0eU1hcGkiOnRydWUsIlYiOiIwLjAuMDAwMCIsIlAiOiJXaW4zMiIsIkFOIjoiTWFpbCIsIldUIjoyfQ%3D%3D%7C0%7C%7C%7C&sdata=uUmlywUOTPpLKFUDOmKINhDmWJJm7x7ezfGUJ4BRlXc%3D&reserved=0)

**Response:**

We thank the reviewer for this important comment. The studies included in the forest plot were conducted exclusively among populations at elevated risk of HIV acquisition (e.g., men who have sex with men, transgender women, and adolescent girls and young women in Sub-Saharan Africa). Accordingly, the findings of our meta-analysis already reflect the performance of HIV testing strategies within high-risk groups.

We also note that the study referenced by the reviewer used an older-generation HIV RDT that is no longer in use and was designed primarily to differentiate HIV-1 from HIV-2 rather than function as a screening test. Nonetheless, we acknowledge that even current-generation RDTs are less sensitive than NAT for identifying very recent HIV infection and have added this point in the Discussion.

Additionally, our review indicates that the absolute number of infections missed by RDTs is extremely small. For example, in the HPTN 083 open-label extension, identifying one missed infection among MSM, trans and gender-diverse people with RDT would require testing 5,305 individuals using NAT. From a public health and resource-allocation perspective, this represents a substantial investment for a modest gain in early detection. Another important consideration is RAMs. Although NAT retrospectively detected HIV infection earlier in 8 of the 10 CAB-LA breakthrough cases with major RAMs, there is currently no prospective evidence that implementing NAT as the routine screening strategy would prevent RAM development and virological failure with INSTI-based antiretroviral regimens. In both HPTN 083 and 084, NAT was conducted retrospectively on stored samples following a positive RDT. In the real world, high-frequency follow-up testing, as done in studies, will likely be infeasible, and turnaround times in resource-limited settings may be lengthy. We also noted that due to low viral loads associated with early infection, several of the cases with major INSTI RAMs, were detected using a single-genome sequencing INSTI drug resistance assay.[2, 3] This research methodology is usually not available for use in clinical diagnostic laboratories and has not been validated in the clinical diagnostic setting.

That said, Smith et al indicated RDTs were sufficient for resource-limited settings as part of the CAB-LA introduction, and NAT added high cost to the programme while providing a small benefit in reducing resistance, and did not improve survival or overall treatment success (<https://www.thelancet.com/journals/lanhiv/article/PIIS2352-3018(22)00365-4/fulltext>). Further, modelling evidence from van de Vijver 2025 (<https://www.abstract-archive.org/Abstract/Share/91175>) using a high incidence setting among men who have sex with men in Thailand also indicated similar findings that the concerns about resistance are on a more individual level and not a public health, population-level issue.

We agree that NAT’s diagnostic advantages should be recognised, and we have revised the Discussion to clarify these considerations while balancing them with programme feasibility and real-world resource constraints.

**Changes:**

[Line 461] In many settings, the use of an RDT as the initial screening test supports same-day PrEP initiation, consistent with WHO recommendations aimed at improving uptake and adherence among key populations. Conversely, whether confirmatory laboratory-based testing should be required prior to LAI-PrEP initiation warrants careful consideration. Confirmatory testing has clear diagnostic value, particularly for detecting early infection, but making it a mandatory prerequisite may inadvertently delay or restrict access in resource-constrained settings. This issue is evident in the US. Despite CDC recommendations, there were only 35% tested by HIV RNA within ± 14 days at CAB-LA initiation [55]. Although missed early diagnoses may have broader economic implications—including the cost of PrEP provision, delayed diagnosis, potential resistance, and onward transmission—the included studies did not provide sufficient prospective data to quantify these downstream effects. A modelling study shows that NAT adds significant cost to CAB-LA rollout programs in LMICs, but provides a small benefit in reducing resistance, and does not improve survival or overall treatment success [26]. Even high-income countries will benefit from RDT-based testing strategies, as evidence shows substantial feasibility constraints of using NAT for routine programmatic implementation [51]. This does not imply that NAT has no role in LAI-PrEP delivery. There may be settings and situations where it could be considered based on available resources.

[Line 499] Our findings showed that NAT could theoretically detect early or rare breakthrough infections during LAI-PrEP use. However, because NAT was used retrospectively on stored samples in HPTN 083 and 084—not prospectively as part of real-time clinical decision-making—there is no prospective evidence that earlier NAT detection would have prevented the development of INSTI resistance-associated mutations. In the real world, high-frequency follow-up testing, as done in studies, will likely be infeasible, and turnaround times in resource-limited settings may be lengthy. As a result, the benefit of a slightly earlier diagnosis may be limited and ultimately not affect the small number of cases with resistance [26]. Based on the data from real-world implementation, despite lower sensitivity of RDTs, there is little to no absolute difference in delayed HIV detection per 1,000 tests when using RDTs among CAB-LA or LEN users at initiation and for CAB-LA users at continuation. Further, using data from HPTN 083 open-label extension [48], detecting one additional case using NAT—missed by RDT at an earlier time point—would require testing 5,305 people using NAT. This suggests that a lot of resources are needed for minimal gain, using a public health lens.

[Line 527] Further, additional modelling suggests that the total number of resistance cases remains small even in very high incidence groups, such as MSM in Thailand.[66] While more sensitive tests could reduce resistance, the impact is minimal.[26, 66] For example, at 100% uptake of CAB-LA with RDTs, 6,200 new infections could be averted, and the difference in the number of INSTI-resistant and transmitted resistance cases remains very low (INSTI resistance: RDT: 94 vs NAT: 36, transmitted resistance: RDT: 15 vs NAT: 6). Thus, while more sensitive HIV testing will prevent some INSTI resistance, given the low INSTI resistance incidence, more sensitive testing is not critical for CAB-LA rollout.[26, 66] The benefits of a programme that is able to scale and get more people on PrEP, and at a fraction of the cost and more access-driven approaches, appear beneficial. Scaling-up LAI-PrEP requires not only simplified HIV testing but also an investment in monitoring for LAI-PrEP-associated resistance within established HIV surveillance programs. This should include monitoring for both LAI-PrEP-associated major RAMs and LAI-PrEP-associated treatment failures.

[Line 592] In conclusion, delayed HIV diagnosis and breakthrough infections were rare and absolute differences in detection between different HIV testing strategies were minimal. Although NAT may offer high sensitivity and early detection—and remains important in selected cases—this did not translate into meaningful clinical or programmatic improvements for LAI-PrEP delivery. In contrast, simpler RDT-based strategies were low-cost, scalable and feasible to implement across a range of settings. As LAI-PrEP continues to scale globally, testing approaches should prioritise flexibility, accessibility, and affordability—factors that are critical for achieving population-level HIV prevention impact.

**6) How will being on PrEP affect our ability to make an HIV diagnosis and does that also warrant consideration in choosing which test to use in PrEP programs for initiation and follow-up? See:** [**https://urldefense.com/v3/__https://pmc.ncbi.nlm.nih.gov/articles/PMC6918508/__;!!NDYExDT0u85SdT4!suyz4DXKqbF9R_592p1f0Y6M5iptFx7Bo-qRozIR19qxM95wTvcVzqUljc7FlDPTPrC8qBnaQUGGqw$**](https://urldefense.com/v3/__https://pmc.ncbi.nlm.nih.gov/articles/PMC6918508/__;!!NDYExDT0u85SdT4!suyz4DXKqbF9R_592p1f0Y6M5iptFx7Bo-qRozIR19qxM95wTvcVzqUljc7FlDPTPrC8qBnaQUGGqw$)**. An algorithm might be an appropriate approach for PrEP program especially if using RDTs. Recommendations for repeat testing might be needed and evaluation for resistance if a patient is infected and missed by RDT and started on PrEP OR they seroconvert on PrEP. The latter would be extremely rare with LAI-PrEP.**

**Response:**

We thank the Reviewer for this important comment. We clarified the distinct clinical considerations for HIV testing strategies for LAI-PrEP in the Introduction.

*“[Line 145] LAI-PrEP, however, may introduce distinct clinical considerations for HIV testing services.[28] Unlike oral PrEP, where HIV protection ceases shortly after stopping medication, LAI-PrEP results in prolonged drug exposure, with subtherapeutic concentrations persisting for weeks to months following the last injection.[38] It is during this period of suboptimal drug levels that individuals who have an undetected HIV infection are at an increased risk of acquiring drug resistance. To address this concern, early policies, including those from WHO, were uncertain whether testing using HIV RDTs and HIVST alone would be sufficient for public health programmes to deliver LAI-PrEP. Current policies from the U.S. Centers for Disease Control and Prevention (CDC) and the International Antiviral Society United States of America (IAS-USA) continue to advise laboratory-based testing and the use of nucleic acid test (NAT) or RNA testing as part of LAI-PrEP delivery.[39, 40]”*

We fully agree with the Reviewer that a tailored HIV testing algorithm is needed for LAI-PrEP, and we have strengthened the Discussion to reflect this. We have now acknowledged the importance of tailored approaches for LAI-PrEP in selected situations where confirmatory testing is needed.

**Changes:**

[Line 461] In many settings, the use of an RDT as the initial screening test supports same-day PrEP initiation, consistent with WHO recommendations aimed at improving uptake and adherence among key populations. Conversely, whether confirmatory laboratory-based testing should be required prior to LAI-PrEP initiation warrants careful consideration. Confirmatory testing has clear diagnostic value, particularly for detecting early infection, but making it a mandatory prerequisite may inadvertently delay or restrict access in resource-constrained settings. This issue is evident in the US. Despite CDC recommendations, there were only 35% tested by HIV RNA within ± 14 days at CAB-LA initiation [55]. Although missed early diagnoses may have broader economic implications—including the cost of PrEP provision, delayed diagnosis, potential resistance, and onward transmission—the included studies did not provide sufficient prospective data to quantify these downstream effects. A modelling study shows that NAT adds significant cost to CAB-LA rollout programs in LMICs, but provides a small benefit in reducing resistance, and does not improve survival or overall treatment success [26]. Even high-income countries will benefit from RDT-based testing strategies, as evidence shows substantial feasibility constraints of using NAT for routine programmatic implementation [51]. This does not imply that NAT has no role in LAI-PrEP delivery. There may be settings and situations where it could be considered based on available resources.

[Line 488] Evidence from HIV-1 seroconversion panels shows the median detection window of 5.9 days for NAT and antigen/antibody (Ag/Ab) laboratory immunoassays, 7.4 days for 4^th^-generation (Ag/Ab) RDTs and 14.9 days for 3^rd^-generation (IgM-sensitive) RDTs. Given the expected window periods of various HIV detection methods and known attenuation of serological responses in the setting of PrEP, it may be pertinent for programmes to consider repeat testing at 2-4 weeks after LAI-PrEP initiation if 3^rd^ generation RDTs are being used and for individuals with recent high-risk exposures. Historically, delayed diagnosis has been a concern due to the high viral loads observed in early infection in PrEP-naive individuals [60, 61]. Given the lower viral loads observed in early LAI-PrEP infections, the transmission risk associated with these infections is likely to be lower than for PrEP-naive individuals [62].

**7) The cost discussion is interesting but putting it into the context of using an RDT and missing a diagnosis - the cost of PrEP, delayed diagnosis, potential resistance, potential further transmission - should be considered too.**

**Response:**

Thank you for this important observation. We agree that the full economic implications of missed early diagnoses—including the cost of PrEP provision, delayed HIV diagnosis, potential emergence of resistance, and potential onward transmission—are critical considerations for programmes. Our review, however, was limited by the data available in the included studies: none provided sufficient prospective evidence to quantify these downstream costs or to compare them meaningfully across testing strategies. Most instances of delayed detection or resistance were identified retrospectively, making it impossible to model real-world cost consequences with confidence. We have added text to the Discussion to clarify that the current evidence does not allow reliable estimation of these economic effects. We also highlight the need for future modelling work that incorporates both programmatic costs and the potential clinical and transmission-related consequences of missed early infections.

That said, modelling data on the cost of using different testing strategies and the impact of resistance has been considered. We refer to Smith et al, which indicated RDTs were sufficient for resource-limited settings as part of the CAB-LA introduction: <https://www.thelancet.com/journals/lanhiv/article/PIIS2352-3018(22)00365-4/fulltext>. They reported that NAT commodities are much more expensive ($22 United States Dollars (USD)) compared with 3^rd^ generation RDT ($4 USD), with a small benefit in reducing resistance but no improvement in survival or overall treatment success. [26].

Our analysis also showed no absolute difference in delayed detections per 1000 tests between different testing strategies, indicating the economic value of the earlier detection for such few cases is likely minimal in terms of savings. We also reported that to detect one additional HIV case, missed by a rapid test, using NAT requires testing 5,305 people with estimated costs of $46,684 - $451,456 [48]. When considering the high costs and limited feasibility of NAT, the overall public health impact of these rare delayed diagnoses is likely small.

**Changes:**

[Line 394] Compared to NAT, the cost of implementing RDT-based testing strategies may be lower *(Low certainty).* A modelling study of CAB-LA in sub-Saharan Africa reported that NAT commodities are much more expensive ($22 United States Dollars (USD)) compared with 3^rd^ generation RDT ($4 USD), with a small benefit in reducing resistance but no improvement in survival or overall treatment success. [26]. Other studies confirm that the cost of NAT is significantly more than that of RDT or laboratory-based immunoassays (Appendix 12) [21-25]. We calculated, based on the reported data, that to detect one additional HIV case, missed by a rapid test, using NAT requires testing 5,305 people with estimated costs of $46,684 - $451,456 [48].

[Line 465] Confirmatory testing has clear diagnostic value, particularly for detecting early infection, but making it a mandatory prerequisite may inadvertently delay or restrict access in resource-constrained settings. This issue is evident in the US. Despite CDC recommendations, there were only 35% tested by HIV RNA within ± 14 days at CAB-LA initiation [55]. Although missed early diagnoses may have broader economic implications—including the cost of PrEP provision, delayed diagnosis, potential resistance, and onward transmission—the included studies did not provide sufficient prospective data to quantify these downstream effects. A modelling study shows that NAT adds significant cost to CAB-LA rollout programs in LMICs, but provides a small benefit in reducing resistance, and does not improve survival or overall treatment success [26].

[Line 511] Further, using data from HPTN 083 open-label extension [48], detecting one additional case using NAT—missed by RDT at an earlier time point—would require testing 5,305 people using NAT. This suggests that a lot of resources are needed for minimal gain, using a public health lens.

[Line 556] We acknowledge the limited number of studies, heterogeneity in study methods, and risk-of-bias concerns. Consequently, the overall certainty of evidence remains very low or low and could be strengthened through further modelling analyses and prospective studies that provide insight into broader economic implications (e.g., the cost of PrEP provision, delayed diagnosis, potential resistance, and onward transmission), optimal testing strategies and approaches for LAI-PrEP, including for new and emerging PrEP agents.

**General editorial requests:**

*	We ask every co-author listed on the manuscript to fill in a contributing author statement, making sure to declare all competing interests. If any of the co-authors have not filled in the statement, we will remind them to do so when the paper is revised. If all statements are not completed in a timely fashion this could hold up the re-review process. If new competing interests are declared later in the revision process, this may also hold up the submission. Should there be a problem getting one of your co-authors to fill in a statement we will be in contact. Please do not add or remove authors without first discussing this with the handling editor. You can see our competing interests policy here: [https://urldefense.com/v3/__http://journals.plos.org/plosmedicine/s/competing-interests__;!!NDYExDT0u85SdT4!suyz4DXKqbF9R_592p1f0Y6M5iptFx7Bo-qRozIR19qxM95wTvcVzqUljc7FlDPTPrC8qBmGW5sB3w$](https://aus01.safelinks.protection.outlook.com/?url=https%3A%2F%2Furldefense.com%2Fv3%2F__http%3A%2F%2Fjournals.plos.org%2Fplosmedicine%2Fs%2Fcompeting-interests__%3B!!NDYExDT0u85SdT4!suyz4DXKqbF9R_592p1f0Y6M5iptFx7Bo-qRozIR19qxM95wTvcVzqUljc7FlDPTPrC8qBmGW5sB3w%24&data=05%7C02%7C%7C25887d699e154cee72ed08de296c2e9d%7Ce21c8db98aa843019657077a0415bdf1%7C0%7C0%7C638993741776889903%7CUnknown%7CTWFpbGZsb3d8eyJFbXB0eU1hcGkiOnRydWUsIlYiOiIwLjAuMDAwMCIsIlAiOiJXaW4zMiIsIkFOIjoiTWFpbCIsIldUIjoyfQ%3D%3D%7C0%7C%7C%7C&sdata=dm1UXOk9N5yYmVHQmbOVvwrV36GCRm8zDAqSavz4wjk%3D&reserved=0).

**Response:** Please inform us if any co-author has not submitted the statement.

*	Please upload any figures associated with your paper as individual TIF or EPS files with 300dpi resolution at resubmission; please read our figure guidelines for more information on our requirements: [https://urldefense.com/v3/__http://journals.plos.org/plosmedicine/s/figures__;!!NDYExDT0u85SdT4!suyz4DXKqbF9R_592p1f0Y6M5iptFx7Bo-qRozIR19qxM95wTvcVzqUljc7FlDPTPrC8qBkF1Z77AA$](https://aus01.safelinks.protection.outlook.com/?url=https%3A%2F%2Furldefense.com%2Fv3%2F__http%3A%2F%2Fjournals.plos.org%2Fplosmedicine%2Fs%2Ffigures__%3B!!NDYExDT0u85SdT4!suyz4DXKqbF9R_592p1f0Y6M5iptFx7Bo-qRozIR19qxM95wTvcVzqUljc7FlDPTPrC8qBkF1Z77AA%24&data=05%7C02%7C%7C25887d699e154cee72ed08de296c2e9d%7Ce21c8db98aa843019657077a0415bdf1%7C0%7C0%7C638993741776905419%7CUnknown%7CTWFpbGZsb3d8eyJFbXB0eU1hcGkiOnRydWUsIlYiOiIwLjAuMDAwMCIsIlAiOiJXaW4zMiIsIkFOIjoiTWFpbCIsIldUIjoyfQ%3D%3D%7C0%7C%7C%7C&sdata=XvVDVPTO4FgM8yhVtWTb0TjZAlN7cndI9tyFrIiPYJE%3D&reserved=0). While revising your submission, we strongly recommend that you use PLOS's NAAS tool ([https://urldefense.com/v3/__https://ngplosjournals.pagemajik.ai/artanalysis__;!!NDYExDT0u85SdT4!suyz4DXKqbF9R_592p1f0Y6M5iptFx7Bo-qRozIR19qxM95wTvcVzqUljc7FlDPTPrC8qBm5hKzi4Q$](https://aus01.safelinks.protection.outlook.com/?url=https%3A%2F%2Furldefense.com%2Fv3%2F__https%3A%2F%2Fngplosjournals.pagemajik.ai%2Fartanalysis__%3B!!NDYExDT0u85SdT4!suyz4DXKqbF9R_592p1f0Y6M5iptFx7Bo-qRozIR19qxM95wTvcVzqUljc7FlDPTPrC8qBm5hKzi4Q%24&data=05%7C02%7C%7C25887d699e154cee72ed08de296c2e9d%7Ce21c8db98aa843019657077a0415bdf1%7C0%7C0%7C638993741776920680%7CUnknown%7CTWFpbGZsb3d8eyJFbXB0eU1hcGkiOnRydWUsIlYiOiIwLjAuMDAwMCIsIlAiOiJXaW4zMiIsIkFOIjoiTWFpbCIsIldUIjoyfQ%3D%3D%7C0%7C%7C%7C&sdata=LNv4cmTE4v072IRielGB0Vk%2FNI9Jg9qahKY7gNZJNvs%3D&reserved=0)) to test your figure files. NAAS can convert your figure files to the TIFF file type and meet basic requirements (such as print size, resolution), or provide you with a report on issues that do not meet our requirements and that NAAS cannot fix.

After uploading your figures to PLOS's NAAS tool - [https://urldefense.com/v3/__https://ngplosjournals.pagemajik.ai/artanalysis__;!!NDYExDT0u85SdT4!suyz4DXKqbF9R_592p1f0Y6M5iptFx7Bo-qRozIR19qxM95wTvcVzqUljc7FlDPTPrC8qBm5hKzi4Q$](https://aus01.safelinks.protection.outlook.com/?url=https%3A%2F%2Furldefense.com%2Fv3%2F__https%3A%2F%2Fngplosjournals.pagemajik.ai%2Fartanalysis__%3B!!NDYExDT0u85SdT4!suyz4DXKqbF9R_592p1f0Y6M5iptFx7Bo-qRozIR19qxM95wTvcVzqUljc7FlDPTPrC8qBm5hKzi4Q%24&data=05%7C02%7C%7C25887d699e154cee72ed08de296c2e9d%7Ce21c8db98aa843019657077a0415bdf1%7C0%7C0%7C638993741776936510%7CUnknown%7CTWFpbGZsb3d8eyJFbXB0eU1hcGkiOnRydWUsIlYiOiIwLjAuMDAwMCIsIlAiOiJXaW4zMiIsIkFOIjoiTWFpbCIsIldUIjoyfQ%3D%3D%7C0%7C%7C%7C&sdata=lnsps7ETKnkPwl0mFEQcbjZXisRSXFzMw%2BW4XeOXJJY%3D&reserved=0), NAAS will process the files provided and display the results in the "Uploaded Files" section of the page as the processing is complete.

If the uploaded figures meet our requirements (or NAAS is able to fix the files to meet our requirements), the figure will be marked as "fixed" above. If NAAS is unable to fix the files, a red "failed" label will appear above.

When NAAS has confirmed that the figure files meet our requirements, please download the file via the download option, and include these NAAS processed figure files when submitting your revised manuscript.

**Response:** We submitted all figures as NAAS processed figure files.

*	Please ensure that the paper adheres to the PLOS Data Availability Policy (see [https://urldefense.com/v3/__http://journals.plos.org/plosmedicine/s/data-availability__;!!NDYExDT0u85SdT4!suyz4DXKqbF9R_592p1f0Y6M5iptFx7Bo-qRozIR19qxM95wTvcVzqUljc7FlDPTPrC8qBlZUPs7AA$](https://aus01.safelinks.protection.outlook.com/?url=https%3A%2F%2Furldefense.com%2Fv3%2F__http%3A%2F%2Fjournals.plos.org%2Fplosmedicine%2Fs%2Fdata-availability__%3B!!NDYExDT0u85SdT4!suyz4DXKqbF9R_592p1f0Y6M5iptFx7Bo-qRozIR19qxM95wTvcVzqUljc7FlDPTPrC8qBlZUPs7AA%24&data=05%7C02%7C%7C25887d699e154cee72ed08de296c2e9d%7Ce21c8db98aa843019657077a0415bdf1%7C0%7C0%7C638993741776951702%7CUnknown%7CTWFpbGZsb3d8eyJFbXB0eU1hcGkiOnRydWUsIlYiOiIwLjAuMDAwMCIsIlAiOiJXaW4zMiIsIkFOIjoiTWFpbCIsIldUIjoyfQ%3D%3D%7C0%7C%7C%7C&sdata=XUhBu1on%2FDwO%2BwycEURTAmfhPF65Zn45fIa76IafOuo%3D&reserved=0)), which requires that all data underlying the study's findings be provided in a repository or as Supporting Information. For data residing with a third party, authors are required to provide instructions with contact information (web or email address) for obtaining the data. Please note that a study author cannot be the contact person for the data. PLOS journals do not allow statements supported by "data not shown" or "unpublished results." For such statements, authors must provide supporting data or cite public sources that include it.

**Response:** We confirmed that all the data included in the manuscript and the Appendix allow for reanalysis and reproducibility.

*	We expect all researchers with submissions to PLOS in which author-generated code underpins the findings in the manuscript to make all author-generated code available without restrictions upon publication of the work. In cases where code is central to the manuscript, we may require the code to be made available as a condition of publication. Authors are responsible for ensuring that the code is reusable and well documented. Please make any custom code available, either as part of your data deposition or as a supplementary file. Please add a sentence to your data availability statement regarding any code used in the study, e.g. "The code used in the analysis is available from Github [URL] and archived in Zenodo [DOI link]" Please review our guidelines at [https://urldefense.com/v3/__https://journals.plos.org/plosmedicine/s/materials-software-and-code-sharing__;!!NDYExDT0u85SdT4!suyz4DXKqbF9R_592p1f0Y6M5iptFx7Bo-qRozIR19qxM95wTvcVzqUljc7FlDPTPrC8qBmioh4cxw$](https://aus01.safelinks.protection.outlook.com/?url=https%3A%2F%2Furldefense.com%2Fv3%2F__https%3A%2F%2Fjournals.plos.org%2Fplosmedicine%2Fs%2Fmaterials-software-and-code-sharing__%3B!!NDYExDT0u85SdT4!suyz4DXKqbF9R_592p1f0Y6M5iptFx7Bo-qRozIR19qxM95wTvcVzqUljc7FlDPTPrC8qBmioh4cxw%24&data=05%7C02%7C%7C25887d699e154cee72ed08de296c2e9d%7Ce21c8db98aa843019657077a0415bdf1%7C0%7C0%7C638993741776967200%7CUnknown%7CTWFpbGZsb3d8eyJFbXB0eU1hcGkiOnRydWUsIlYiOiIwLjAuMDAwMCIsIlAiOiJXaW4zMiIsIkFOIjoiTWFpbCIsIldUIjoyfQ%3D%3D%7C0%7C%7C%7C&sdata=4EJ%2FDxBqYosc3AgQ0q4FcO13M%2Bbl%2FipBFq9LU6Y510Q%3D&reserved=0) and ensure that your code is shared in a way that follows best practice and facilitates reproducibility and reuse. Because Github depositions can be readily changed or deleted, we encourage you to make a permanent DOI'd copy (e.g. in Zenodo) and provide the URL.

**Response:** Not applicable.

*	Please confirm that all authors have declared any COIs.

**Response:** We have confirmed with all authors.

*	Please note that we require the inclusion of funders' websites (URLs).

**Changes:** [Line 236] **Financial disclosure statement**

This systematic review is commissioned by WHO (who.int). JJO is funded by the Australian National Health Medical Research Council Investigator Grant (GNT1193955) (www.nhmrc.gov.au).

*	Please ensure that the study is reported according to the appropriate guideline and include the completed checklist as Supporting Information. When completing the checklist, please use section and paragraph numbers, rather than page numbers. Please add the following statement, or similar, to the Methods: "This study is reported as per PRISMA guideline (S1 Checklist)."

**Changes:** [Line 173] This systematic review was conducted following the WHO Handbook for Guideline Development[34] and the Cochrane Handbook for Systematic Reviews of Interventions[35] and reported findings according to the PRISMA Reporting Guideline (S1 Checklist).[36]

**FORMATTING – GENERAL**

*	Abstract: Please structure your abstract using the PLOS Medicine headings (Background, Methods and Findings, Conclusions). Please combine the Methods and Findings sections into one section.

**Response:** The current abstract adheres to the journal's format.

*	At this stage, we ask that you include a short, non-technical Author Summary of your research to make findings accessible to a wide audience that includes both scientists and non-scientists. The Author Summary should immediately follow the Abstract in your revised manuscript. This text is subject to editorial change and should be distinct from the scientific abstract. Ideally each sub-heading should contain 2-3 single sentence, concise bullet points containing the most salient points from your study. In the final bullet point of 'What Do These Findings Mean?', please include the main limitations of the study in non-technical language. Please see our author guidelines for more information: [https://urldefense.com/v3/__https://journals.plos.org/plosmedicine/s/revising-your-manuscript*loc-author-summary__;Iw!!NDYExDT0u85SdT4!suyz4DXKqbF9R_592p1f0Y6M5iptFx7Bo-qRozIR19qxM95wTvcVzqUljc7FlDPTPrC8qBkAOocifw$](https://aus01.safelinks.protection.outlook.com/?url=https%3A%2F%2Furldefense.com%2Fv3%2F__https%3A%2F%2Fjournals.plos.org%2Fplosmedicine%2Fs%2Frevising-your-manuscript*loc-author-summary__%3BIw!!NDYExDT0u85SdT4!suyz4DXKqbF9R_592p1f0Y6M5iptFx7Bo-qRozIR19qxM95wTvcVzqUljc7FlDPTPrC8qBkAOocifw%24&data=05%7C02%7C%7C25887d699e154cee72ed08de296c2e9d%7Ce21c8db98aa843019657077a0415bdf1%7C0%7C0%7C638993741776982374%7CUnknown%7CTWFpbGZsb3d8eyJFbXB0eU1hcGkiOnRydWUsIlYiOiIwLjAuMDAwMCIsIlAiOiJXaW4zMiIsIkFOIjoiTWFpbCIsIldUIjoyfQ%3D%3D%7C0%7C%7C%7C&sdata=n1U0QrEbn2INczTOgsIHvcD3L94BsKjxL0UoMrdcbxc%3D&reserved=0).

**Changes:** [Line 82]

**Author summary**

**Why was this study done?**

- Long-acting injectable pre-exposure prophylaxis (LAI-PrEP) is expanding rapidly, but it was unclear whether rapid diagnostic tests (RDTs), laboratory-based tests, or nucleic acid testing (NAT) are the most appropriate HIV testing strategies for safe, effective and scalable delivery.
- Early HIV infections during LAI-PrEP can occur with lower viral loads, raising concerns about delayed diagnosis and the potential development of drug resistance.
- Policymakers require timely evidence on the accuracy, safety, and feasibility of HIV testing approaches to guide emerging global recommendations for LAI-PrEP rollout.

**What did the researchers do and find?**

- We conducted a systematic review of HIV testing strategies, including RDTs, laboratory-based immunoassays, and NAT, and assessed their effects on clinical, diagnostic, and resource-use outcomes for LAI-PrEP delivery.
- Twenty-two studies from 15 countries were included, comprising 13 observational cohorts and 7 non-comparator studies.
- RDTs provided faster results, quicker treatment initiation, and lower testing costs, while NAT occasionally produced false-positive results that led to unnecessary delays or PrEP discontinuation.
- NAT might have identified HIV before resistance emerged in eight cases, but no prospective evidence demonstrated that NAT as part of real-time clinical decision-making would have prevented resistance.
- RDTs and laboratory tests detected HIV slightly later than NAT, but this had no significant impact at the population level.

**What do these findings mean?**

- RDT-based testing approaches appear safe, feasible, and practical for supporting LAI-PrEP scale-up, though the overall certainty of evidence is low and additional data are needed.
- NAT may detect a small number of infections earlier, but its routine use faces substantial feasibility challenges, and detecting one additional early infection would require testing more than 5,300 people at high cost.
- These findings suggest that RDTs can serve as the base testing strategy for LAI-PrEP programmes, while acknowledging the need for continued research, ongoing surveillance, and the selective use of NAT in specific clinical situations.

*	Please express the main results with 95% CIs as well as p values. When reporting p values please report as p<0.001 and where higher as the exact p value p=0.002, for example. Throughout, suggest reporting statistical information as follows to improve clarity for the reader "22% (95% CI [13%,28%]; p</=)". Please be sure to define all numerical values at first use.

**Changes:** [Line 61] HIV detection rates were similar across strategies (9/8171 (RDT) vs 14/8171 (NAT) (Odds ratio (OR) 0.66 (95% confidence interval: 0.29–1.50; P=0.87)), with no difference in adverse events.

[Line 321] RDTs may make little to no difference in the detection of new HIV infections (9/8171) compared to NAT-based strategies (14/8171), with an odds ratio of 0.66 (95% CI: 0.29–1.50; P=0.87) and an absolute difference of 1 fewer infection per 1000 tests (range: 1 fewer to 1 more).

*	Please include page numbers and line numbers in the manuscript file. Use continuous line numbers (do not restart the numbering on each page).

**Response:** We have included page numbers and line numbers in the manuscript file.

*	Please cite the reference numbers in square brackets. Citations should precede punctuation.

**Response:** We have updated the reference numbers throughout the manuscript.

**FIGURES AND TABLES**

*	Please provide titles and legends for all figures and tables (including those in Supporting Information files).

**Response:** We have updated our manuscript and the appendix files accordingly.

*	Please define all abbreviations used in each figure/table (including those in Supporting Information files).

**Response:** We have updated our manuscript and the appendix files accordingly.

*	Please consider avoiding the use of red and green in order to make your figure more accessible to those with color blindness.

**Response:** We do not use red and green in our manuscript.

**SUPPLEMENTARY MATERIAL**

*	Please note that supplementary material will be posted as supplied by the authors. Therefore, please amend it according to the relevant comments outlined here.

**Response:** We have updated our appendix file accordingly.

*	Please cite your Supporting Information as outlined here: [https://urldefense.com/v3/__https://journals.plos.org/plosmedicine/s/supporting-information__;!!NDYExDT0u85SdT4!suyz4DXKqbF9R_592p1f0Y6M5iptFx7Bo-qRozIR19qxM95wTvcVzqUljc7FlDPTPrC8qBk-oS7WZw$](https://aus01.safelinks.protection.outlook.com/?url=https%3A%2F%2Furldefense.com%2Fv3%2F__https%3A%2F%2Fjournals.plos.org%2Fplosmedicine%2Fs%2Fsupporting-information__%3B!!NDYExDT0u85SdT4!suyz4DXKqbF9R_592p1f0Y6M5iptFx7Bo-qRozIR19qxM95wTvcVzqUljc7FlDPTPrC8qBk-oS7WZw%24&data=05%7C02%7C%7C25887d699e154cee72ed08de296c2e9d%7Ce21c8db98aa843019657077a0415bdf1%7C0%7C0%7C638993741776998632%7CUnknown%7CTWFpbGZsb3d8eyJFbXB0eU1hcGkiOnRydWUsIlYiOiIwLjAuMDAwMCIsIlAiOiJXaW4zMiIsIkFOIjoiTWFpbCIsIldUIjoyfQ%3D%3D%7C0%7C%7C%7C&sdata=fxeKGL5sVgKvCUsiQDhJiejKsroo7K1bL9I2a7saNbs%3D&reserved=0)

**Response:** We have updated our manuscript and appendix files according to the guideline.

**REFERENCES**

*	PLOS uses the numbered citation (citation-sequence) method and first six authors, et al.

**Response:** We have updated our citation accordingly.

*	Please ensure that journal name abbreviations match those found in the National Center for Biotechnology Information (NCBI) databases ([https://urldefense.com/v3/__http://www.ncbi.nlm.nih.gov/nlmcatalog/journals__;!!NDYExDT0u85SdT4!suyz4DXKqbF9R_592p1f0Y6M5iptFx7Bo-qRozIR19qxM95wTvcVzqUljc7FlDPTPrC8qBmziykrPw$](https://aus01.safelinks.protection.outlook.com/?url=https%3A%2F%2Furldefense.com%2Fv3%2F__http%3A%2F%2Fwww.ncbi.nlm.nih.gov%2Fnlmcatalog%2Fjournals__%3B!!NDYExDT0u85SdT4!suyz4DXKqbF9R_592p1f0Y6M5iptFx7Bo-qRozIR19qxM95wTvcVzqUljc7FlDPTPrC8qBmziykrPw%24&data=05%7C02%7C%7C25887d699e154cee72ed08de296c2e9d%7Ce21c8db98aa843019657077a0415bdf1%7C0%7C0%7C638993741777014090%7CUnknown%7CTWFpbGZsb3d8eyJFbXB0eU1hcGkiOnRydWUsIlYiOiIwLjAuMDAwMCIsIlAiOiJXaW4zMiIsIkFOIjoiTWFpbCIsIldUIjoyfQ%3D%3D%7C0%7C%7C%7C&sdata=8wpaO%2FcNnVzBbznAq90TsZMRAutoLprBM6OKQ%2FyTYfU%3D&reserved=0)), and are appropriately formatted and capitalised.

**Response:** We have updated our references accordingly.

*	Where website addresses are cited, please include the complete URL and specify the date of access (e.g. [accessed: 12/06/2023]).

**Response:** We have updated our references accordingly.

*	Please also see [https://urldefense.com/v3/__https://journals.plos.org/plosmedicine/s/submission-guidelines*loc-references__;Iw!!NDYExDT0u85SdT4!suyz4DXKqbF9R_592p1f0Y6M5iptFx7Bo-qRozIR19qxM95wTvcVzqUljc7FlDPTPrC8qBkFFlRy5w$](https://aus01.safelinks.protection.outlook.com/?url=https%3A%2F%2Furldefense.com%2Fv3%2F__https%3A%2F%2Fjournals.plos.org%2Fplosmedicine%2Fs%2Fsubmission-guidelines*loc-references__%3BIw!!NDYExDT0u85SdT4!suyz4DXKqbF9R_592p1f0Y6M5iptFx7Bo-qRozIR19qxM95wTvcVzqUljc7FlDPTPrC8qBkFFlRy5w%24&data=05%7C02%7C%7C25887d699e154cee72ed08de296c2e9d%7Ce21c8db98aa843019657077a0415bdf1%7C0%7C0%7C638993741777029405%7CUnknown%7CTWFpbGZsb3d8eyJFbXB0eU1hcGkiOnRydWUsIlYiOiIwLjAuMDAwMCIsIlAiOiJXaW4zMiIsIkFOIjoiTWFpbCIsIldUIjoyfQ%3D%3D%7C0%7C%7C%7C&sdata=Zr4Z1aVsSx8xwtQCue4Y1mP1r1V6NvP%2Benw%2FPXWgI48%3D&reserved=0) for further details on reference formatting.

**Response:** We have updated our references accordingly.

**SYSTEMATIC REVIEWS & META-ANALYSES**

*	Please report your SR/MA according to the PRISMA guidelines provided at the EQUATOR site. [https://urldefense.com/v3/__http://www.equator-network.org/reporting-guidelines/prisma/__;!!NDYExDT0u85SdT4!suyz4DXKqbF9R_592p1f0Y6M5iptFx7Bo-qRozIR19qxM95wTvcVzqUljc7FlDPTPrC8qBnbTTG7Rw$](https://aus01.safelinks.protection.outlook.com/?url=https%3A%2F%2Furldefense.com%2Fv3%2F__http%3A%2F%2Fwww.equator-network.org%2Freporting-guidelines%2Fprisma%2F__%3B!!NDYExDT0u85SdT4!suyz4DXKqbF9R_592p1f0Y6M5iptFx7Bo-qRozIR19qxM95wTvcVzqUljc7FlDPTPrC8qBnbTTG7Rw%24&data=05%7C02%7C%7C25887d699e154cee72ed08de296c2e9d%7Ce21c8db98aa843019657077a0415bdf1%7C0%7C0%7C638993741777045078%7CUnknown%7CTWFpbGZsb3d8eyJFbXB0eU1hcGkiOnRydWUsIlYiOiIwLjAuMDAwMCIsIlAiOiJXaW4zMiIsIkFOIjoiTWFpbCIsIldUIjoyfQ%3D%3D%7C0%7C%7C%7C&sdata=dC61xKUfNcZ%2FZH7ZlTtiRpDRKc1ltWBrbvty6mRzbx0%3D&reserved=0). Please provide the completed PRISMA checklist as Supporting Information. When completing the checklist, please use section and paragraph numbers, rather than page numbers. Please add the following statement, or similar, to the Methods: "This study is reported as per the Preferred Reporting Items for Systematic Reviews and Meta-Analyses (PRISMA) guideline (S1 Checklist)."

**Response:** We have included the statement and submitted S1 Checklist as Supporting Information.

*	Abstract: Please report your abstract according to PRISMA for abstracts ([https://urldefense.com/v3/__https://doi.org/10.1371/journal.pmed.1001419__;!!NDYExDT0u85SdT4!suyz4DXKqbF9R_592p1f0Y6M5iptFx7Bo-qRozIR19qxM95wTvcVzqUljc7FlDPTPrC8qBnHfWSVYQ$](https://aus01.safelinks.protection.outlook.com/?url=https%3A%2F%2Furldefense.com%2Fv3%2F__https%3A%2F%2Fdoi.org%2F10.1371%2Fjournal.pmed.1001419__%3B!!NDYExDT0u85SdT4!suyz4DXKqbF9R_592p1f0Y6M5iptFx7Bo-qRozIR19qxM95wTvcVzqUljc7FlDPTPrC8qBnHfWSVYQ%24&data=05%7C02%7C%7C25887d699e154cee72ed08de296c2e9d%7Ce21c8db98aa843019657077a0415bdf1%7C0%7C0%7C638993741777060533%7CUnknown%7CTWFpbGZsb3d8eyJFbXB0eU1hcGkiOnRydWUsIlYiOiIwLjAuMDAwMCIsIlAiOiJXaW4zMiIsIkFOIjoiTWFpbCIsIldUIjoyfQ%3D%3D%7C0%7C%7C%7C&sdata=Xn1nSRtxCIqY1sWeBfAw6PL8kTB8nFgKSsE6O9NWZvc%3D&reserved=0)) following the PLOS Medicine abstract structure (Background, Methods and Findings, Conclusions). Please ensure you provide dates of search, data sources, number of studies included, types of study designs included, eligibility criteria, and synthesis/appraisal methods.

**Response:** We have updated the abstract and included PRISMA for abstracts in the S1 Checklist file as Supporting Information.

*	Please note that we expect searches to be updated to within 6 months of the time of submission.

**Response:** Our systematic review was conducted to inform the development of the new WHO guidelines on HIV testing strategies for LAI-PrEP (ISBN: 978-92-4-011160-8). The primary database searches were conducted in October 2024, and, in parallel, we incorporated all additional studies identified by the WHO evidence synthesis team through their continuous surveillance process. This resulted in the inclusion of studies published up to April 2025.

As a result, the evidence base used in this review reflects literature identified within six months of submission and includes all studies available to the WHO guideline development process.

*	Please provide a/amend your detailed Financial Disclosure statement. This is published with the article. It must therefore be completed in full sentences and contain the exact wording you wish to be published.

**Changes:** [Line 236] **Financial disclosure statement**

This systematic review is commissioned by WHO (who.int). JJO is funded by the Australian National Health Medical Research Council Investigator Grant (GNT1193955) (www.nhmrc.gov.au).

The funders had no role in study design, data collection and analysis, decision to publish, or preparation of the manuscript. WHO members (CCJ, CBDS, MR, HLI, HAS, MP, BM, CL, HP, LC, PA) received a salary from WHO and participated in data collection, data interpretation and writing of the report.

**Further comments from the editor**

1. State what role the funders took in the study. If the funders had no role in your study, please state: “The funders had no role in study design, data collection and analysis, decision to publish, or preparation of the manuscript.”

**Changes:** [Line 236] **Financial disclosure statement**

This systematic review is commissioned by WHO (who.int). JJO is funded by the Australian National Health Medical Research Council Investigator Grant (GNT1193955) (www.nhmrc.gov.au).

The funders had no role in study design, data collection and analysis, decision to publish, or preparation of the manuscript. WHO members (CCJ, CBDS, MR, HLI, HAS, MP, BM, CL, HP, LC, PA) received a salary from WHO and participated in data collection, data interpretation and writing of the report.

2. If any authors received a salary from any of your funders, please state which authors and which funders.

**Changes:** [Line 236] **Financial disclosure statement**

This systematic review is commissioned by WHO (who.int). JJO is funded by the Australian National Health Medical Research Council Investigator Grant (GNT1193955) (www.nhmrc.gov.au).

The funders had no role in study design, data collection and analysis, decision to publish, or preparation of the manuscript. WHO members (CCJ, CBDS, MR, HLI, HAS, MP, BM, CL, HP, LC, PA) received a salary from WHO and participated in data collection, data interpretation and writing of the report.

2) We ask that a manuscript source file is provided at Revision. Please upload your manuscript file as a .doc, .docx, .rtf or .tex.

**Response:** We upload our manuscript in .docx file.

3) Please provide separate figure files in .tif or .eps format.

For more information about figure files please see our guidelines:

[https://urldefense.com/v3/__https://journals.plos.org/plosmedicine/s/figures__;!!NDYExDT0u85SdT4!vQtMwfzTWANTcZ6G71L4mReyIw5HYAY7m7abgy1QJZjpjiowbyTel9bZCCCCHKCWd_9JmWBoL5c85A$](https://aus01.safelinks.protection.outlook.com/?url=https%3A%2F%2Furldefense.com%2Fv3%2F__https%3A%2F%2Fjournals.plos.org%2Fplosmedicine%2Fs%2Ffigures__%3B!!NDYExDT0u85SdT4!vQtMwfzTWANTcZ6G71L4mReyIw5HYAY7m7abgy1QJZjpjiowbyTel9bZCCCCHKCWd_9JmWBoL5c85A%24&data=05%7C02%7C%7C9bc8fde310f64b0ab24b08de2b3b6b7d%7Ce21c8db98aa843019657077a0415bdf1%7C0%7C0%7C638995731357972495%7CUnknown%7CTWFpbGZsb3d8eyJFbXB0eU1hcGkiOnRydWUsIlYiOiIwLjAuMDAwMCIsIlAiOiJXaW4zMiIsIkFOIjoiTWFpbCIsIldUIjoyfQ%3D%3D%7C0%7C%7C%7C&sdata=hqKW3J01%2FKJV%2BVCsv51CrKInEF7zHbEihHLUp7X0kl8%3D&reserved=0)

[https://urldefense.com/v3/__https://journals.plos.org/plosmedicine/s/figures*loc-file-requirements__;Iw!!NDYExDT0u85SdT4!vQtMwfzTWANTcZ6G71L4mReyIw5HYAY7m7abgy1QJZjpjiowbyTel9bZCCCCHKCWd_9JmWDWyO-DFQ$](https://aus01.safelinks.protection.outlook.com/?url=https%3A%2F%2Furldefense.com%2Fv3%2F__https%3A%2F%2Fjournals.plos.org%2Fplosmedicine%2Fs%2Ffigures*loc-file-requirements__%3BIw!!NDYExDT0u85SdT4!vQtMwfzTWANTcZ6G71L4mReyIw5HYAY7m7abgy1QJZjpjiowbyTel9bZCCCCHKCWd_9JmWDWyO-DFQ%24&data=05%7C02%7C%7C9bc8fde310f64b0ab24b08de2b3b6b7d%7Ce21c8db98aa843019657077a0415bdf1%7C0%7C0%7C638995731357990090%7CUnknown%7CTWFpbGZsb3d8eyJFbXB0eU1hcGkiOnRydWUsIlYiOiIwLjAuMDAwMCIsIlAiOiJXaW4zMiIsIkFOIjoiTWFpbCIsIldUIjoyfQ%3D%3D%7C0%7C%7C%7C&sdata=eBKaDLZqxD1zYodsk%2Bd3esSfobKc%2FsqeMf7CfFqjQaQ%3D&reserved=0)

**Response:** We upload a separate figure file in .tif format.

1) Please amend your detailed Financial Disclosure statement. This is published with the article. It must therefore be completed in full sentences and contain the exact wording you wish to be published.

-State the initials, alongside each funding source, of each author to receive each grant. For example: "This work was supported by the National Institutes of Health (### to AM; ### to CJ) and the National Science Foundation (### to AM)."

-State what role the funders took in the study. If the funders had no role in your study, please state: “The funders had no role in study design, data collection and analysis, decision to publish, or preparation of the manuscript.”

-If any authors received a salary from any of your funders, please state which authors and which funders."

**Change:** [Line 236] **Financial disclosure statement**

This systematic review is commissioned by WHO (who.int). JJO is funded by the Australian National Health Medical Research Council Investigator Grant (GNT1193955) (www.nhmrc.gov.au).

The funders had no role in study design, data collection and analysis, decision to publish, or preparation of the manuscript. WHO members (CCJ, CBDS, MR, HLI, HAS, MP, BM, CL, HP, LC, PA) received a salary from WHO and participated in data collection, data interpretation and writing of the report.

2) Please ensure that all Figure files have corresponding citations and legends within the manuscript.

Currently, Figure [3] in your submission file inventory does not have an in-text citation. If the figure is no longer to be included as part of the submission, please remove it from the file inventory

**Response:** We only have Figure 1 and Figure 2. However, Figure 2 consists of two images.

3) We have noticed that you have uploaded Supporting Information files, but you have not included a list of legends. Please add a full list of legends for your Supporting Information files before or after the references list.

# Change: [Line 904] Supporting information

**Appendix:** S1 Appendix. Search strategy protocol, S2 Appendix. Definition of outcomes, S3 Appendix. Risk of bias assessment, S4 Appendix. Certainty of evidence (GRADE), S5 Appendix. Characteristics and testing strategies of each program, S6 Appendix. Time to linkage to care, S7 Appendix. PrEP holds or discontinuation from false positive, S8 Appendix. HIV positivity, S9 Appendix. Diagnostic accuracy and performance, S10 Appendix. Turnaround time for results, S11 Appendix. Resistance-associated mutations, and S12 Appendix. Costs of HIV tests (USD 2024).

**S1 Checklist:** PRISMA 2020 Checklist and PRISMA 2020 for Abstract Checklist.

1.	Van de Vijver D, Van Kampen J, Wongsa A, Janamnuaysook R, Mesplede T, Matrajt L, Jordan MR, Phanuphak N, Dimitrov D. Evaluating HIV testing strategies to reduce drug resistance during CAB-LA rollout in Thailand: a modelling study. Presented at: 13th IAS Conference on HIV Science (IAS 2025); Abstract C3.2025.

2.	Halvas EK, Joseph KW, Brandt LD, Guo S, Sobolewski MD, Jacobs JL, et al. HIV-1 viremia not suppressible by antiretroviral therapy can originate from large T cell clones producing infectious virus. J Clin Invest. 2020;130(11):5847-57. doi: 10.1172/jci138099. PubMed PMID: 33016926; PubMed Central PMCID: PMCPMC7598056.

3.	Palmer S, Kearney M, Maldarelli F, Halvas EK, Bixby CJ, Bazmi H, et al. Multiple, linked human immunodeficiency virus type 1 drug resistance mutations in treatment-experienced patients are missed by standard genotype analysis. J Clin Microbiol. 2005;43(1):406-13. doi: 10.1128/jcm.43.1.406-413.2005. PubMed PMID: 15635002; PubMed Central PMCID: PMCPMC540111.

4.	Cox SN, Wu L, Wittenauer R, Clark S, Roberts DA, Nwogu IB, et al. Impact of HIV self-testing for oral pre-exposure prophylaxis scale-up on drug resistance and HIV outcomes in western Kenya: a modelling study. Lancet HIV. 2024;11(3):e167-e75. Epub 20240129. doi: 10.1016/s2352-3018(23)00268-0. PubMed PMID: 38301668; PubMed Central PMCID: PMCPMC10896737.

5.	Smith J, Bansi-Matharu L, Cambiano V, Dimitrov D, Bershteyn A, van de Vijver D, et al. Predicted effects of the introduction of long-acting injectable cabotegravir pre-exposure prophylaxis in sub-Saharan Africa: a modelling study. Lancet HIV. 2023;10(4):e254-e65. Epub 2023/01/16. doi: 10.1016/S2352-3018(22)00365-4. PubMed PMID: 36642087; PubMed Central PMCID: PMCPMC10065903.

6.	Baron J, Tchelidze T, LaBrot B, Yao J, Mayer KH, Kuritzkes D, et al. High Rates of Missed HIV Testing Among Oral PrEP Users in the United States From 2018-2021: A National Assessment on Compliance With HIV Testing Recommendations of the CDC PrEP Guidelines. Open Forum Infect Dis. 2024;11(5):ofae254. Epub 20240523. doi: 10.1093/ofid/ofae254. PubMed PMID: 38798900; PubMed Central PMCID: PMCPMC11127479.

7.	Marzinke MA, Fogel JM, Wang Z, Piwowar-Manning E, Kofron R, Moser A, et al. Extended Analysis of HIV Infection in Cisgender Men and Transgender Women Who Have Sex with Men Receiving Injectable Cabotegravir for HIV Prevention: HPTN 083. Antimicrob Agents Chemother. 2023;67(4). doi: <https://dx.doi.org/10.1128/aac.00053-23>. PubMed PMID: 2024279292.

8.	Vijver Dvd, Kampen Jv, Wongsa A, Janamnuaysook R, Mesplede T, Matrajt L, et al. OAC0205 Evaluating HIV testing strategies to reduce drug resistance during CAB-LA rollout in Thailand. A modelling study. International AIDS Society 2025; 13-17 July 2025; Kigali, Rwanda & Virtual2025.

9.	Lorenzetti L, Dinh N, van der Straten A, Fonner V, Ridgeway K, Rodolph M, et al. Systematic review of the values and preferences regarding the use of injectable pre-exposure prophylaxis to prevent HIV acquisition. J Int AIDS Soc. 2023;26(S2):e26107. doi: <https://doi.org/10.1002/jia2.26107>.

10.	Fajardo E, Lastrucci C, Jolivet P, DiChiara M, Baptista da Silva C, Msimanga B, et al. Should antigen-antibody rapid diagnostic tests be used to detect acute HIV infection? A systematic review and meta-analysis of diagnostic performance. medRxiv. 2025:2025.10.14.25338004. doi: 10.1101/2025.10.14.25338004.

11.	Smith MK, Rutstein SE, Powers KA, Fidler S, Miller WC, Eron JJ, Jr., et al. The detection and management of early HIV infection: a clinical and public health emergency. J Acquir Immune Defic Syndr. 2013;63 Suppl 2(0 2):S187-99. doi: 10.1097/QAI.0b013e31829871e0. PubMed PMID: 23764635; PubMed Central PMCID: PMCPMC4015137.

12.	Fiebig EW, Wright DJ, Rawal BD, Garrett PE, Schumacher RT, Peddada L, et al. Dynamics of HIV viremia and antibody seroconversion in plasma donors: implications for diagnosis and staging of primary HIV infection. AIDS. 2003;17(13):1871-9. doi: 10.1097/00002030-200309050-00005. PubMed PMID: 12960819.

13.	Broyles LN, Luo R, Boeras D, Vojnov L. The risk of sexual transmission of HIV in individuals with low-level HIV viraemia: a systematic review. Lancet. 2023;402(10400):464-71. Epub 20230722. doi: 10.1016/s0140-6736(23)00877-2. PubMed PMID: 37490935; PubMed Central PMCID: PMCPMC10415671.

14.	le Roux SM, Odayar J, Sutcliffe CG, Salvatore PP, de Broucker G, Dowdy D, et al. Cost-effectiveness of point-of-care versus centralised, laboratory-based nucleic acid testing for diagnosis of HIV in infants: a systematic review of modelling studies. Lancet HIV. 2023;10(5):e320-e31. doi: 10.1016/s2352-3018(23)00029-2. PubMed PMID: 37149292; PubMed Central PMCID: PMCPMC10175481.

15.	Parikh UM, Altamirano J, Safa H, Hazra A, Shukla P, Hedberg T, et al. Early Virologic Success on ART following Breakthrough Infection on CAB-LA PrEP. HIVR4P2024; Lima, Peru.

16.	Koss CA, Gandhi M, Halvas EK, Okochi H, Chu C, Glidden DV, et al. First Case of HIV Seroconversion With Integrase Resistance Mutations on Long-Acting Cabotegravir for Prevention in Routine Care. Open forum infectious diseases. 2024;11(9):ofae468. doi: <https://dx.doi.org/10.1093/ofid/ofae468>.

17.	Delany-Moretlwe S, Hughes JP, Bock P, Ouma SG, Hunidzarira P, Kalonji D, et al. Cabotegravir for the prevention of HIV-1 in women: results from HPTN 084, a phase 3, randomised clinical trial. Lancet. 2022;399(10337):1779-89. doi: <https://dx.doi.org/10.1016/S0140-6736%2822%2900538-4>. PubMed PMID: 2017986913.

18.	Eshleman SH, Fogel JM, Piwowar-Manning E, Chau G, Cummings V, Agyei Y, et al. Characterization of Human Immunodeficiency Virus (HIV) Infections in Women Who Received Injectable Cabotegravir or Tenofovir Disoproxil Fumarate/Emtricitabine for HIV Prevention: HPTN 084. The Journal of infectious diseases. 2022;225(10):1741-9. doi: <https://dx.doi.org/10.1093/infdis/jiab576>.

19.	Eshleman SH, Fogel JM, Halvas EK, Piwowar-Manning E, Marzinke MA, Kofron R, et al. HIV RNA Screening Reduces Integrase Strand Transfer Inhibitor Resistance Risk in Persons Receiving Long-Acting Cabotegravir for HIV Prevention. The Journal of infectious diseases. 2022;226(12):2170-80. doi: <https://dx.doi.org/10.1093/infdis/jiac415>.

20.	Fogel JM, Piwowar-Manning E, Moser A, Hill T, Ahmed S, Cummings V, et al. Evaluation of Xpert point-of-care assays for detection of HIV infection in persons using long-acting cabotegravir for pre-exposure prophylaxis. Microbiology spectrum. 2024:e0030724. doi: <https://dx.doi.org/10.1128/spectrum.00307-24>.

21.	Jamieson L, Johnson LF, Nichols BE, Delany-Moretlwe S, Hosseinipour MC, Russell C, et al. Relative cost-effectiveness of long-acting injectable cabotegravir versus oral pre-exposure prophylaxis in South Africa based on the HPTN 083 and HPTN 084 trials: a modelled economic evaluation and threshold analysis. The lancet HIV. 2022;9(12):e857-e67. doi: <https://dx.doi.org/10.1016/S2352-3018(22)00251-X>.

22.	Landovitz RJ, Donnell D, Clement ME, Hanscom B, Cottle L, Coelho L, et al. Cabotegravir for HIV Prevention in Cisgender Men and Transgender Women. The New England journal of medicine. 2021;385(7):595-608. doi: <https://dx.doi.org/10.1056/NEJMoa2101016>.

23.	Landovitz RJ, Hanscom BS, Clement ME, Tran HV, Kallas EG, Magnus M, et al. Efficacy and safety of long-acting cabotegravir compared with daily oral tenofovir disoproxil fumarate plus emtricitabine to prevent HIV infection in cisgender men and transgender women who have sex with men 1 year after study unblinding: a secondary analysis of the phase 2b and 3 HPTN 083 randomised controlled trial. The Lancet HIV. 2023;10(12):e767-e78. doi: <https://dx.doi.org/10.1016/S2352-3018%2823%2900261-8>. PubMed PMID: 2028335393.

24.	Marzinke MA, Fogel JM, Wang Z, Piwowar-Manning E, Kofron R, Moser A, et al. Extended Analysis of HIV Infection in Cisgender Men and Transgender Women Who Have Sex with Men Receiving Injectable Cabotegravir for HIV Prevention: HPTN 083. Antimicrobial agents and chemotherapy. 2023;67(4):e0005323. doi: <https://dx.doi.org/10.1128/aac.00053-23>.

25.	Marzinke MA, Grinsztejn B, Fogel JM, Piwowar-Manning E, Li M, Weng L, et al. Characterization of Human Immunodeficiency Virus (HIV) Infection in Cisgender Men and Transgender Women Who Have Sex With Men Receiving Injectable Cabotegravir for HIV Prevention: HPTN 083. The Journal of infectious diseases. 2021;224(9):1581-92. doi: <https://dx.doi.org/10.1093/infdis/jiab152>.

26.	Neilan AM, Landovitz RJ, Le MH, Grinsztejn B, Freedberg KA, McCauley M, et al. Cost-Effectiveness of Long-Acting Injectable HIV Preexposure Prophylaxis in the United States A Cost-Effectiveness Analysis. Annals of Internal Medicine. 2022;175(4):479-89. doi: <https://dx.doi.org/10.7326/M21-1548>. PubMed PMID: 2017979852.

27.	O'Brien P, Campbell K, Anderson SJ, Cornic L, Davis A, Schroeder M. EE281 Cost-Effectiveness of Every Two Month Cabotegravir Long-Acting (CAB-LA) for Pre-Exposure Prophylaxis (PREP) Compared with Daily Oral Tenofovir Disoproxil Fumarate (TDF)/ Emtricitabine (FTC) As Prep to Prevent HIV-1 in the UK. Value in Health. 2024;27(6 Supplement):S109. doi: <https://dx.doi.org/10.1016/j.jval.2024.03.580>. PubMed PMID: 2032788449.

28.	World Health Organization. Consolidated Guidelines on HIV Testing Services: 5Cs: Consent, Confidentiality, Counselling, Correct Results and Connection 2015 2015 [cited 2025 31 March]. Available from: <https://www.ncbi.nlm.nih.gov/books/NBK316024/>.

29.	World Health Organization. Consolidated Guideline on Differentiated HIV Testing Services Geneva, Switzerland: World Health Organization; [cited 2025 31 March]. Available from: <https://www.who.int/publications/i/item/9789240096394>.

30.	World Health Organization survey on HIV testing for CAB-LA PrEP programs/research (2024).

31.	Stevens DR, Vrana CJ, Dlin RE, Korte JE. A Global Review of HIV Self-testing: Themes and Implications. AIDS Behav. 2018;22(2):497-512. doi: 10.1007/s10461-017-1707-8.

32.	Pant Pai N, Balram B, Shivkumar S, Martinez-Cajas JL, Claessens C, Lambert G, et al. Head-to-head comparison of accuracy of a rapid point-of-care HIV test with oral versus whole-blood specimens: a systematic review and meta-analysis. Lancet Infect Dis. 2012;12(5):373-80. Epub 20120124. doi: 10.1016/s1473-3099(11)70368-1. PubMed PMID: 22277215.

33.	Heidt B, Siqueira WF, Eersels K, Diliën H, van Grinsven B, Fujiwara RT, et al. Point of Care Diagnostics in Resource-Limited Settings: A Review of the Present and Future of PoC in Its Most Needed Environment. Biosensors. 2020;10(10):133. PubMed PMID: doi:10.3390/bios10100133.

34.	World Health Organization. WHO handbook for guideline development Geneva: World Health Organization; 2014 [updated 2014; cited 2025 31 March]. 2nd:[Available from: <https://iris.who.int/handle/10665/145714>.

35.	Higgins J, Thomas J, Chandler J, Cumpston M, Li T, Page M, et al. Cochrane Handbook for Systematic Reviews of Interventions version 6.5.

36.	Preferred Reporting Items for Systematic reviews and Meta-Analyses (PRISMA) [cited 2025 31 March]. Available from: <https://www.prisma-statement.org/>.

37.	Guyatt GH, Oxman AD, Vist GE, Kunz R, Falck-Ytter Y, Alonso-Coello P, et al. GRADE: an emerging consensus on rating quality of evidence and strength of recommendations. BMJ. 2008;336(7650):924-6. doi: 10.1136/bmj.39489.470347.AD. PubMed PMID: 18436948; PubMed Central PMCID: PMCPMC2335261.

38.	Han K, Patel P, McCallister S, Rinehart AR, Gandhi Y, Spreen W, et al. Long-acting cabotegravir pharmacokinetics with and without oral lead-in for HIV PrEP. Antimicrob Agents Chemother. 2024;68(6):e0147523. Epub 20240506. doi: 10.1128/aac.01475-23. PubMed PMID: 38709006; PubMed Central PMCID: PMCPMC11620484.

39.	U.S. Centre of Disease Control and Prevention. HIV Nexus: CDC Resources for Clinicians [cited 2025 31 March]. Available from: <https://www.cdc.gov/hivnexus/hcp/prep/index.html>.

40.	Gandhi RT, Landovitz RJ, Sax PE, Smith DM, Springer SA, Günthard HF, et al. Antiretroviral Drugs for Treatment and Prevention of HIV in Adults: 2024 Recommendations of the International Antiviral Society–USA Panel. JAMA. 2025;333(7):609-28. doi: 10.1001/jama.2024.24543.
