# Supplementary material for: Rapid diagnostic tests, laboratory-based immunoassay and nucleic acid testing strategies for long-acting injectable pre-exposure prophylaxis: A systematic review and meta-analysis
Source: PLoS Med. 2026 Apr 16;23(4):e1005030. doi: 10.1371/journal.pmed.1005030 (PMC13102303; doi:10.1371/journal.pmed.1005030)
Supplement: Attachment — Submitted filename: Response to Reviewers and Editors 20260318.docx [file pmed.1005030.s017.docx]

Dear Dr. Ong,

Thank you very much for re-submitting your manuscript "Balancing Accuracy, Accessibility, and Cost: HIV Testing Strategies for Long-Acting Injectable Pre-Exposure Prophylaxis: A Systematic Review of Rapid, Laboratory-Based Immunoassay and Nucleic Acid Testing Strategies" (PMEDICINE-D-25-02056R2) for review by PLOS Medicine.

I have discussed the paper with my colleagues and the academic editor and it was also seen again by 2 reviewers. I am pleased to say that provided the remaining editorial and production issues are dealt with we are planning to accept the paper for publication in the journal.

The remaining issues that need to be addressed are listed at the end of this email. Any accompanying reviewer attachments can be seen via the link below. Please take these into account before resubmitting your manuscript:

***Please note while forming your response, if your article is accepted, you may have the opportunity to make the peer review history publicly available. The record will include editor decision letters (with reviews) and your responses to reviewer comments. If eligible, we will contact you to opt in or out.***

In revising the manuscript for further consideration here, please ensure you address the specific points made by each reviewer and the editors. In your rebuttal letter you should indicate your response to the reviewers' and editors' comments and the changes you have made in the manuscript. Please submit a clean version of the paper as the main article file. A version with changes marked must also be uploaded as a marked up manuscript file.

Please also check the guidelines for revised papers at <https://urldefense.com/v3/__http://journals.plos.org/plosmedicine/s/revising-your-manuscript__;!!NDYExDT0u85SdT4!tVchh_T5GqMhi4AMEjcar1ezl9ObdAywPl1EcYeTmjIuXZcnewmLuNMOkXTLlfGR25fCxId10Gzqwg$> for any that apply to your paper. If you haven't already, we ask that you provide a short, non-technical Author Summary of your research to make findings accessible to a wide audience that includes both scientists and non-scientists. The Author Summary should immediately follow the Abstract in your revised manuscript. This text is subject to editorial change and should be distinct from the scientific abstract.

In addition to these revisions, you may need to complete some formatting changes, which you will receive in a follow up email. A member of our team will be in touch with a set of requests shortly. If you do not receive a separate email within a few days, please assume that checks have been completed, and no additional changes are required.

We expect to receive your revised manuscript within 1 week. Please email us ([plosmedicine@plos.org](mailto:plosmedicine@plos.org)) if you have any questions or concerns.

We ask every co-author listed on the manuscript to fill in a contributing author statement. If any of the co-authors have not filled in the statement, we will remind them to do so when the paper is revised. If all statements are not completed in a timely fashion this could hold up the re-review process. Should there be a problem getting one of your co-authors to fill in a statement we will be in contact. YOU MUST NOT ADD OR REMOVE AUTHORS UNLESS YOU HAVE ALERTED THE EDITOR HANDLING THE MANUSCRIPT TO THE CHANGE AND THEY SPECIFICALLY HAVE AGREED TO IT.

Please ensure that the paper adheres to the PLOS Data Availability Policy (see [https://urldefense.com/v3/__http://journals.plos.org/plosmedicine/s/data-availability__;!!NDYExDT0u85SdT4!tVchh_T5GqMhi4AMEjcar1ezl9ObdAywPl1EcYeTmjIuXZcnewmLuNMOkXTLlfGR25fCxIfn4StlxA$](https://aus01.safelinks.protection.outlook.com/?url=https%3A%2F%2Furldefense.com%2Fv3%2F__http%3A%2F%2Fjournals.plos.org%2Fplosmedicine%2Fs%2Fdata-availability__%3B!!NDYExDT0u85SdT4!tVchh_T5GqMhi4AMEjcar1ezl9ObdAywPl1EcYeTmjIuXZcnewmLuNMOkXTLlfGR25fCxIfn4StlxA%24&data=05%7C02%7C%7C9da7ae2f2e82442c18bd08de7c0522d4%7Ce21c8db98aa843019657077a0415bdf1%7C0%7C0%7C639084558763831620%7CUnknown%7CTWFpbGZsb3d8eyJFbXB0eU1hcGkiOnRydWUsIlYiOiIwLjAuMDAwMCIsIlAiOiJXaW4zMiIsIkFOIjoiTWFpbCIsIldUIjoyfQ%3D%3D%7C0%7C%7C%7C&sdata=jMcMh2dEOQ8WhfEerH5aQp7Ca3nD0MiRfuMjv2ZmmH0%3D&reserved=0)), which requires that all data underlying the study's findings be provided in a repository or as Supporting Information. For data residing with a third party, authors are required to provide instructions with contact information for obtaining the data. PLOS journals do not allow statements supported by "data not shown" or "unpublished results." For such statements, authors must provide supporting data or cite public sources that include it.

To enhance the reproducibility of your results, we recommend that you deposit your laboratory protocols in protocols.io, where a protocol can be assigned its own identifier (DOI) such that it can be cited independently in the future. Additionally, PLOS ONE offers an option to publish peer-reviewed clinical study protocols. Read more information on sharing protocols at [https://urldefense.com/v3/__https://plos.org/protocols?utm_medium=editorial-email&utm_source=authorletters&utm_campaign=protocols__;!!NDYExDT0u85SdT4!tVchh_T5GqMhi4AMEjcar1ezl9ObdAywPl1EcYeTmjIuXZcnewmLuNMOkXTLlfGR25fCxIe1DAL4gQ$](https://aus01.safelinks.protection.outlook.com/?url=https%3A%2F%2Furldefense.com%2Fv3%2F__https%3A%2F%2Fplos.org%2Fprotocols%3Futm_medium%3Deditorial-email%26utm_source%3Dauthorletters%26utm_campaign%3Dprotocols__%3B!!NDYExDT0u85SdT4!tVchh_T5GqMhi4AMEjcar1ezl9ObdAywPl1EcYeTmjIuXZcnewmLuNMOkXTLlfGR25fCxIe1DAL4gQ%24&data=05%7C02%7C%7C9da7ae2f2e82442c18bd08de7c0522d4%7Ce21c8db98aa843019657077a0415bdf1%7C0%7C0%7C639084558763850959%7CUnknown%7CTWFpbGZsb3d8eyJFbXB0eU1hcGkiOnRydWUsIlYiOiIwLjAuMDAwMCIsIlAiOiJXaW4zMiIsIkFOIjoiTWFpbCIsIldUIjoyfQ%3D%3D%7C0%7C%7C%7C&sdata=ho1BQKW%2BxV%2Bl8x9z2MW25tzxgBblsOzDxotI5z%2B4k6o%3D&reserved=0)

Please review your reference list to ensure that it is complete and correct. If you have cited papers that have been retracted, please include the rationale for doing so in the manuscript text, or remove these references and replace them with relevant current references. Any changes to the reference list should be mentioned in the rebuttal letter that accompanies your revised manuscript.

Please note, when your manuscript is accepted, an uncorrected proof of your manuscript will be published online ahead of the final version, unless you've already opted out via the online submission form. If, for any reason, you do not want an earlier version of your manuscript published online or are unsure if you have already indicated as such, please let the journal staff know immediately at [plosmedicine@plos.org](mailto:plosmedicine@plos.org).

If you have any questions in the meantime, please contact me or the journal staff on [plosmedicine@plos.org](mailto:plosmedicine@plos.org).

We look forward to receiving the revised manuscript by Mar 12 2026 11:59PM.

Sincerely,

Alison Farrell, Ph.D.

Senior Editor

PLOS Medicine

plosmedicine.org

**Response:** We thank the Senior Editor for the opportunity to revise and improve our manuscript. We have provided a point-by-point response to all comments, with corresponding revisions and line numbers indicated in the rebuttal letter. Both clean and tracked-changes versions of the revised manuscript have been submitted. The Author Summary is included in the manuscript, the supporting data are provided in the Supplementary Materials, and the reference list has been checked for completeness and accuracy.

Best regards,

Prof. Ong and Dr Tieosapjaroen,

on behalf of the authors.

------------------------------------------------------------

Requests from Editors:

*** Please review your text for claims of novelty or primacy (e.g. 'for the first time') and remove this language. In addition, please check that any use of statistical terms (such as trend or significant) are supported by the data, and if not please remove them.**

**Response:** We thank the Editors for your comment. We have reviewed the manuscript and found no claims of novelty, and the use of statistical terms is supported by the data.

*** In the last sentence of the Abstract Methods and Findings section, please describe the main limitation(s) of the study's methodology.**

**Change:** [Line 77] We noted that our assessment of performance accuracy in different testing strategies may introduce selection bias.

*** In the author summary, in the final bullet point of 'What Do These Findings Mean?', please include the main limitations of the study in non-technical language.**

**Change:** [Line 125] We noted that our evaluation of the accuracy of different testing strategies may have introduced bias in the selection of participants.

*** Please provide titles and legends for all figures and tables (including those in Supporting Information files). Please define all acronyms used in each figure or table in its corresponding legend**.

**Response:** Thank you for this comment. We provided titles and legends for all figures and tables. We have added acronyms to all tables and figures.

**Change:** [Line 313] HPTN= HIV Prevention Trials Network,

[Line 377] Chi² = Cochran’s chi-squared statistic for heterogeneity, CI=Confidence interval, df = Degrees of freedom, I² = Higgins’ I-squared statistic for heterogeneity, LAI-PrEP=Long-acting injectable pre-exposure prophylaxis, M-H=Mantel–Haenszel method, NAT = Nucleic acid test, P = P-value, RDT = Rapid diagnostic test, Tau² = Tau-squared, Z = Z-statistic for the overall pooled effect.

[Table S10] Ag/Ab=antigen/antibody; CAB-LA=Long-acting cabotegravir; HIVST=HIV self-testing; Gen=Generation; NI=No information available; N=no; PrEP=pre-exposure prophylaxis; RDT=rapid diagnostic test; WHO=World Health Organization; Y=Yes

[Table S11] Gen=Generation,

[Table S13] RNA=ribonucleic acid

[Table S14] LAI-PrEP = long-acting injectable pre-exposure prophylaxis, PrEP=pre-exposure prophylaxis,

[Table S15] Gen=Generation,

[Table S16] RDT=Rapid diagnostic test, RNA=Ribonucleic acid

[Table S17] Ab=Antibody, Ag=Antigen

[Table S19] RAM: resistance-associated mutation; LAI-PrEP: long-acting injectable pre-exposure prophylaxis; TDF/FTC: Tenofovir disoproxil fumarate/emtricitabine

*** Please ensure that where relevant figures include 95% CIs.**

**Response:** They are included.

*** Please ensure that all abbreviations are defined at first use throughout the text.**

**Response:** We confirmed and changed ‘odd ratio’ to ‘OR’ in Line 318 and Line 342.

*** Please confirm that all numbers presented in the abstract are present and identical to numbers presented in the main manuscript text.**

**Response:** We have reviewed and confirmed that the numbers in the abstract and main manuscript are identical.

*** Please confirm that your title complies with PLOS Medicine's style. Your title must be nondeclarative and not a question. It should begin with main concept if possible. "Effect of" should be used only if causality can be inferred, i.e., for an RCT. Please place the study design ("A randomized controlled trial," "A retrospective study," "A modelling study," etc.) in the subtitle (ie, after a colon). We suggest: “Laboratory-Based Immunoassay and Nucleic Acid HIV Testing Strategies for Long-Acting Injectable Pre-Exposure Prophylaxis: A Systematic Review**

**Response:** We thank the Editors for this comment. We have changed the title to ‘Rapid Diagnostic Tests, Laboratory-Based Immunoassay and Nucleic Acid Testing Strategies for Long-Acting Injectable Pre-Exposure Prophylaxis: A Systematic Review’.

*** It appears that one or more study authors is affiliated with one or more of the agencies that funded the study (WHO). Thus, the statement “The funders had no role in study design, data collection and analysis, decision to publish, or preparation of the manuscript” does not apply. Please revise the Financial Disclosure accordingly, as in "[Author name] is [author's role] at [funding agency]. The funders had no other role in study design…..”**

**Please provide the funder URL(s)**

**Response:** Thank you for this comment. We have revised the Financial Disclosure statement to clarify the relationship between the authors and the WHO.

The coauthors (CCJ, CBDS, MR, HLI, HAS, MP, BM, CL, HP, LC, PA) are affiliated with WHO; however, the resources supporting this work were derived from external donor funding provided to WHO to support the development of WHO guidelines. This systematic review contributed to the evidence base informing that process but was conducted independently by the authors. The external donors had no role in the study design, data collection, analysis, interpretation, decision to publish, or preparation of the manuscript.

The Financial Disclosure statement has been updated accordingly, and the relevant funder URL has been added.

**Change:** [Line 247]

This work was supported through resources provided to WHO **(**<https://www.who.int>) by external donors to support WHO guideline development activities. The external donors had no role in the study design, data collection and analysis, decision to publish, or preparation of the manuscript. CCJ, CBDS, MR, HLI, HAS, MP, BM, CL, HP, LC, PA, who are affiliated with and receive a salary from the WHO, participated in data collection, data interpretation and writing of the report. JJO is funded by the Australian National Health Medical Research Council Investigator Grant (GNT1193955) (www.nhmrc.gov.au).

**Please indicate what LAI-PrEP is for in the first sentence of the Background (i.e. prevention of HIV acquisition)**

**Change:** [Line 39] **Background:** Long-acting injectable pre-exposure prophylaxis (LAI-PrEP) is a highly effective biomedical intervention for the prevention of HIV acquisition. There is strong interest among communities and policymakers for LAI-PrEP scale-up, accelerating the demand for clear guidance on testing approaches that balance accuracy with scalability.

**Please clarify in the Abstract and Methods that you included studies to April 2025—based on the rebuttal information**

**Change:** [Line 52] **Methods and findings:** We searched databases and retrieved studies up to April 8, 2025,

[Line 193] This resulted in the inclusion of studies published up to 8 April 2025.

Line 52: please remove period after methodology.

**Change:** [Line 58] Certainty of evidence was determined using the GRADE methodology (Prospero: CRD42024605562).

Please explain in the Abstract what the 8171 and 2483 denominators are.

**Change:** [Line 65] Among 8171 LAI-PrEP users in four randomised controlled trials, HIV detection rates were similar across strategies (9/8171 (RDT) vs 14/8171 (NAT) (Odds ratio (OR) 0.66 (95% confidence interval: 0.29–1.50; P=0.87)), with no difference in adverse events.

[Line 73] In the HPTN 083 trial, NAT use was occasionally associated with false-positive results, leading to unnecessary PrEP holds or discontinuation (7/2483).

**Line 70: change ‘ensuring’ to ‘which can ensure’**

**Change:** [Line 76] RDT-based testing strategies have comparable accuracy to laboratory-based strategies and are more accessible and scalable, which can ensure that testing does not become a barrier to accessing or continuing LAI-PrEP.

**Line 73: delete ‘,with self-testing as an option’, as per reviewer comment.**

**Change:** [Line 82] As countries expand access to LAI-PrEP amid increasingly constrained resources, adoption of new WHO guidance supporting the use of RDTs can enable simpler, more affordable, and user-centred HIV testing approaches.

**In the Abstract, a clearer explanation of why testing might differ for LAI vs oral PrEP is needed in the Background.**

**Change:** [Line 43] Unlike oral pre-exposure prophylaxis, LAI-PrEP may overcome adherence challenges, such as difficulty with frequent clinic visits. However, LAI-PrEP results in prolonged subtherapeutic drug levels after discontinuation, which can increase the risk of drug resistance among those who have an undetected HIV infection.

**Please clarify definition for HIVST ( used to define both the test and self-testing but defined as self-testing)**

**Change:** [Line 134] HIV self-testing (HIVST), a process in which individuals collect their own specimen, perform the test, and interpret the result themselves,

**Line 82: what is meant by ‘early HIV infections’?**

**Change:** [Line 94] Early or recently acquired HIV infections may not be detected by some testing strategies during LAI-PrEP because of lower viral loads, raising concerns about delayed diagnosis and the potential development of drug resistance.

**Author summary: please clarify the distinction between a lab-based test and a NAT. Suggest to use laboratory-based immunoassays (if correct) throughout to avoid confusion.**

**Response:** We have changed laboratory-based tests to laboratory-based immunoassay tests.

**Change:** [Line 89] Long-acting injectable pre-exposure prophylaxis (LAI-PrEP) is expanding rapidly, but it was unclear whether rapid diagnostic tests (RDTs), laboratory-based immunoassay tests (which detect HIV antibodies or antigen), or nucleic acid testing (NAT) (which detects HIV RNA) are the most appropriate HIV testing strategies for safe, effective and scalable delivery.

**This statement, line 154, “This review extends beyond comparing the analytical accuracy of HIV tests” is not necessary in either the Abstract or the Introduction.**

**Change:** [Line 47] This systematic review evaluates how different HIV testing strategies, including rapid diagnostic tests (RDTs), laboratory-based immunoassays and nucleic acid testing (NAT), affect clinical utility and programme delivery.

[Line 171] This review evaluates how different testing approaches, including RDTs, HIVST, laboratory-based immunoassays and NAT techniques, affect clinical outcomes and programme delivery for LAI-PrEP.

**Persons not funders should be included in the Acknowledgments.**

**Change:** [Line 619] We acknowledge the participants of the WHO open call for their contribution of data.

**Please provide a clean version of the Supplementary material**

**Response:** We have submitted the clean and tracked change version of the Supplementary material.

**Please be advised that in general we require that systematic review searches are updated to within 6 months of final submission. In this case we feel it would be worth performing the search and adding a sentence indicating the results (i.e. have there been additional relevant studies since April 2025 or not?) for full transparency for readers.**

**Change:** [Line 590] Lastly, prior to final submission of this paper, we identified seven additional publications [60-66]. Four were full-text reports of studies already included in this review [61-64], two reported behavioural and psychosocial factors among LAI-PrEP users [60, 65] and one abstract about the impact of rapid HIV testing and self-testing on LEN effectiveness and resistance.[66]

**Please make sure the manuscript metadata matches the manuscript.**

**Response:** We have checked and confirmed that the manuscript metadata matched the manuscript.

**"* Please report your SR/MA according to the PRISMA guidelines provided at the EQUATOR site.**

[**https://urldefense.com/v3/__http://www.equator-network.org/reporting-guidelines/prisma/__;!!NDYExDT0u85SdT4!tVchh_T5GqMhi4AMEjcar1ezl9ObdAywPl1EcYeTmjIuXZcnewmLuNMOkXTLlfGR25fCxIfUcx7ZNw$**](https://aus01.safelinks.protection.outlook.com/?url=https%3A%2F%2Furldefense.com%2Fv3%2F__http%3A%2F%2Fwww.equator-network.org%2Freporting-guidelines%2Fprisma%2F__%3B!!NDYExDT0u85SdT4!tVchh_T5GqMhi4AMEjcar1ezl9ObdAywPl1EcYeTmjIuXZcnewmLuNMOkXTLlfGR25fCxIfUcx7ZNw%24&data=05%7C02%7C%7C9da7ae2f2e82442c18bd08de7c0522d4%7Ce21c8db98aa843019657077a0415bdf1%7C0%7C0%7C639084558763868759%7CUnknown%7CTWFpbGZsb3d8eyJFbXB0eU1hcGkiOnRydWUsIlYiOiIwLjAuMDAwMCIsIlAiOiJXaW4zMiIsIkFOIjoiTWFpbCIsIldUIjoyfQ%3D%3D%7C0%7C%7C%7C&sdata=Hr2hMVFQ2mzPgf1jMVOZS6WdiZ47fV1XhyPrRg0Af3o%3D&reserved=0)

**When completing the PRSIMA checklist, please use section and paragraph numbers, rather than page numbers (page numbers are presently used).**

**Response:** Thank you for this comment. This systematic review and meta-analysis was conducted and reported in accordance with the PRISMA 2020 guidelines. We have updated the PRISMA 2020 Checklist to include section and paragraph numbers.

Please add the following statement, or similar, to the Methods: ""This study is reported as per the Preferred Reporting Items for Systematic Reviews and Meta-Analyses (PRISMA) guideline (S1 Checklist)."""

**Change:** [Line 182] This study is reported as per the Preferred Reporting Items for Systematic Reviews and Meta-Analyses (PRISMA) guideline (S1 Checklist) [7].

*** Please provide the beginning and end dates of your search.**

**Change:** [Line 188] We searched OVID Medline, OVID Embase, CINAHL, and Cochrane Central Register of Controlled Trials for studies published from inception to October 18, 2024. We also searched electronic conference abstracts from the International AIDS Society (AIDS), the Conference on Retroviruses and Opportunistic Infections (CROI), and the HIV Research for Prevention (HIVR4P) for all available abstracts. Additionally, relevant studies were retrieved from the WHO research team and the WHO open call. This resulted in the inclusion of studies published up to 8 April 2025.

*** Please report your abstract according to PRISMA for abstracts, following the PLOS Medicine abstract structure (Background, Methods and Findings, Conclusions)** [**https://urldefense.com/v3/__http://www.plosmedicine.org/article/info:doi/10.1371/journal.pmed.1001419__;!!NDYExDT0u85SdT4!tVchh_T5GqMhi4AMEjcar1ezl9ObdAywPl1EcYeTmjIuXZcnewmLuNMOkXTLlfGR25fCxId7HAvThg$**](https://aus01.safelinks.protection.outlook.com/?url=https%3A%2F%2Furldefense.com%2Fv3%2F__http%3A%2F%2Fwww.plosmedicine.org%2Farticle%2Finfo%3Adoi%2F10.1371%2Fjournal.pmed.1001419__%3B!!NDYExDT0u85SdT4!tVchh_T5GqMhi4AMEjcar1ezl9ObdAywPl1EcYeTmjIuXZcnewmLuNMOkXTLlfGR25fCxId7HAvThg%24&data=05%7C02%7C%7C9da7ae2f2e82442c18bd08de7c0522d4%7Ce21c8db98aa843019657077a0415bdf1%7C0%7C0%7C639084558763886184%7CUnknown%7CTWFpbGZsb3d8eyJFbXB0eU1hcGkiOnRydWUsIlYiOiIwLjAuMDAwMCIsIlAiOiJXaW4zMiIsIkFOIjoiTWFpbCIsIldUIjoyfQ%3D%3D%7C0%7C%7C%7C&sdata=LixySj280pvy7x%2B3JZgD%2FlxKeHYl3XtLk7rFolHTIdY%3D&reserved=0) .

**Response:** Thank you for this comment. The abstract has been structured according to the PLOS Medicine format (Background, Methods and Findings, Conclusions) and reported in line with the PRISMA 2020 for Abstracts guidelines. The completed PRISMA 2020 Abstract Checklist was included in the submission (page 5 of the S1 Checklist file). We have also revised the abstract to explicitly report the method used to assess risk of bias.

**Change:** [Line 59] Risk Of Bias In Non-randomised Studies of Interventions, Version 2 (ROBINS-I V2) assessment tool was used to assess bias for non-randomised comparative studies.

**Comments from Reviewers:**

**Reviewer #1: The authors have responded thoroughly and persuasively to the majority of comments by the reviewers. However, a few issues remain:**

**1. The authors still insert in the conclusions of the abstract that self-testing should be considered, when their review (and their data) do not support this recommendation.**

**Response:** We thank the Reviewer for this comment. We have removed reference to self-testing in the abstract.

**Change:** [Line 82] As countries expand access to LAI-PrEP amid increasingly constrained resources, adoption of new WHO guidance supporting the use of RDTs can enable simpler, more affordable, and user-centred HIV testing approaches.

**2. Lines 141-143: The issue isn't so much about breakthrough infections when the levels of the LAI are low, but rather giving people monotherapy who have undetected infection at inception of LAI.**

**Response:** Thank you for this comment. We have revised the paragraph to emphasise the key concern of monotherapy in individuals with undiagnosed HIV infection.

**Change:** [Line 154] LAI-PrEP, however, may introduce distinct clinical considerations for HIV testing services [8]. A key concern is the potential initiation of LAI-PrEP in individuals with undiagnosed HIV infection, which may result in functional monotherapy and increase the risk of developing drug resistance. Unlike oral PrEP, where HIV protection ceases shortly after stopping medication, LAI-PrEP results in prolonged drug exposure, with subtherapeutic concentrations persisting for weeks to months following the last injection [9]. To address this concern, early policies, including those from WHO, were uncertain whether testing using HIV RDTs and HIVST alone would be sufficient for public health programmes to deliver LAI-PrEP. Current policies from the U.S. Centers for Disease Control and Prevention (CDC) and the International Antiviral Society United States of America (IAS-USA) continue to advise laboratory-based immunoassay testing and the use of nucleic acid test (NAT) or RNA testing as part of LAI-PrEP delivery [10, 11].

**3. Throughout, there is mostly discussion of NAT vs. RDT. What about confirmatory lab-based Ag/Ab testing that is not mandatory? What is the cost of that testing, and hwo would that affect the cost calculations (lines 389-390 and Table 2)?**

**Response:** The cost estimates reported in Table 2 ($22 for NAT vs. $4 for RDT) were derived from a modelling study conducted in Sub-Saharan Africa (Smith et al., 2023). We provide the full set of cost comparisons, including those for confirmatory laboratory-based Ag/Ab testing, in **S12 Appendix**. As shown in that table, the cost estimates for laboratory-based Ag/Ab testing are less robust and more variable across sources. Therefore, we were unable to make additional statements regarding the average cost of this testing in the manuscript.

**Reviewer #3: Thank you for addressing my comments so thoroughly. I do still think that the tables in the Supplemental information - namely S4 and S5 should be included in the main paper. However, I defer to the editors as they might have a preference given the size of the tables. These data are important to share so perhaps links to the supplement could be included when they are referenced in the main paper.**

**Response:** Dear Editors, in this review, we grouped evidence by study/program rather than by publication because several studies generated multiple publications, often reporting overlapping cohorts, different study periods, or related outcomes. We also incorporated additional information obtained directly from study authors and the WHO team to ensure completeness. Presenting these as individual papers in the main table would therefore create duplication and potentially confuse the interpretation of study-level implementation findings. Therefore, in the footnote in Table 1’s legend, we refer to S5 Appendix, where readers can find the detailed study-level characteristics.

1.	Del Duca G, Moschese D, Maresca M, Fusetti C, Esvan R, Cossu MV, et al. Mental health, stigma and risk perception among early adopters of HIV PrEP with LA Cabotegravir in Italy: a multicenter cross-sectional and implementation study. Int J Infect Dis. 2026;163:108273. Epub 20251201. doi: 10.1016/j.ijid.2025.108273. PubMed PMID: 41338462.

2.	Cox S, Andreatta K, Hendricks MR, Li J, Kintu A, Brown LB, et al. Resistance Analyses of Lenacapavir, Emtricitabine/Tenofovir Alafenamide and Emtricitabine/Tenofovir Disoproxil Fumarate in the PURPOSE 1 and 2 Studies. J Infect Dis. 2026;233(1):e203–e11. doi: 10.1093/infdis/jiaf533. PubMed PMID: 41130593; PubMed Central PMCID: PMCPMC12811884.

3.	Zhu W, Delaney K, Huang YA, Patel RR, Kourtis AP, Hoover KW. Real-world HIV RNA and Antigen/Antibody Testing Among People Who Use Long-acting Injectable Preexposure Prophylaxis. Clin Infect Dis. 2025;81(4):e102–e7. doi: 10.1093/cid/ciaf173. PubMed PMID: 40176250; PubMed Central PMCID: PMCPMC12344593.

4.	Kusemererwa S, Nayiga B, Lebina L, Bekker LG, Philips A, Matharu LB, et al. Implementing oral (event-driven and daily) and long-acting pre-exposure prophylaxis in mobile men in sub-Saharan Africa: a phase 3b, open-label, hybrid type 2 implementation and effectiveness trial (MOBILE MEN). Trials. 2025;26(1):486. Epub 20251110. doi: 10.1186/s13063-025-09138-5. PubMed PMID: 41214706; PubMed Central PMCID: PMCPMC12604281.

5.	Phillips AN, Hickey MD, Shade SB, Kabami J, Ayieko J, Revill P, et al. Dynamic choice HIV prevention with long-acting injectable cabotegravir pre-exposure prophylaxis in east, central, southern, and west Africa: a cost-effectiveness modelling analysis. Lancet HIV. 2025;12(10):e701–e11. Epub 20250911. doi: 10.1016/s2352-3018(25)00169-9. PubMed PMID: 40946710; PubMed Central PMCID: PMCPMC12854106.

6.	Rossotti R, Cavazza G, Caruso E, Baiguera C, Gennaro ED, Bana NB, et al. Patterns and Correlates of Initiation of Long-Acting Injectable Cabotegravir for Preexposure Prophylaxis: A Case-Control Analysis. Sex Transm Dis. 2026;53(3):183–6. Epub 20251104. doi: 10.1097/olq.0000000000002272. PubMed PMID: 41186399.

7.	Preferred Reporting Items for Systematic reviews and Meta-Analyses (PRISMA) [cited 2025 31 March]. Available from: <https://www.prisma-statement.org/>.

8.	World Health Organization. Consolidated guidelines on HIV testing services: 5Cs: Consent, Confidentiality, Counselling, Correct Results and Connection 2015 2015 [cited 2025 31 March]. Available from: <https://www.ncbi.nlm.nih.gov/books/NBK316024/>.

9.	Han K, Patel P, McCallister S, Rinehart AR, Gandhi Y, Spreen W, et al. Long-acting cabotegravir pharmacokinetics with and without oral lead-in for HIV PrEP. Antimicrob Agents Chemother. 2024;68(6):e0147523. Epub 20240506. doi: 10.1128/aac.01475-23. PubMed PMID: 38709006; PubMed Central PMCID: PMCPMC11620484.

10.	U.S. Centre of Disease Control and Prevention. HIV Nexus: CDC Resources for Clinicians [cited 2025 31 March]. Available from: <https://www.cdc.gov/hivnexus/hcp/prep/index.html>.

11.	Gandhi RT, Landovitz RJ, Sax PE, Smith DM, Springer SA, Günthard HF, et al. Antiretroviral drugs for treatment and prevention of HIV in adults: 2024 recommendations of the international antiviral society–USA panel. JAMA. 2025;333(7):609–28. doi: 10.1001/jama.2024.24543.
